# Supplementary figures and images for: The PRC2.1 subcomplex opposes G1 progression through regulation of CCND1 and CCND2 (part 1 of 2)
Source: eLife. 2025 Feb 4;13:RP97577. doi: 10.7554/eLife.97577 (PMC11793871; doi:10.7554/eLife.97577)

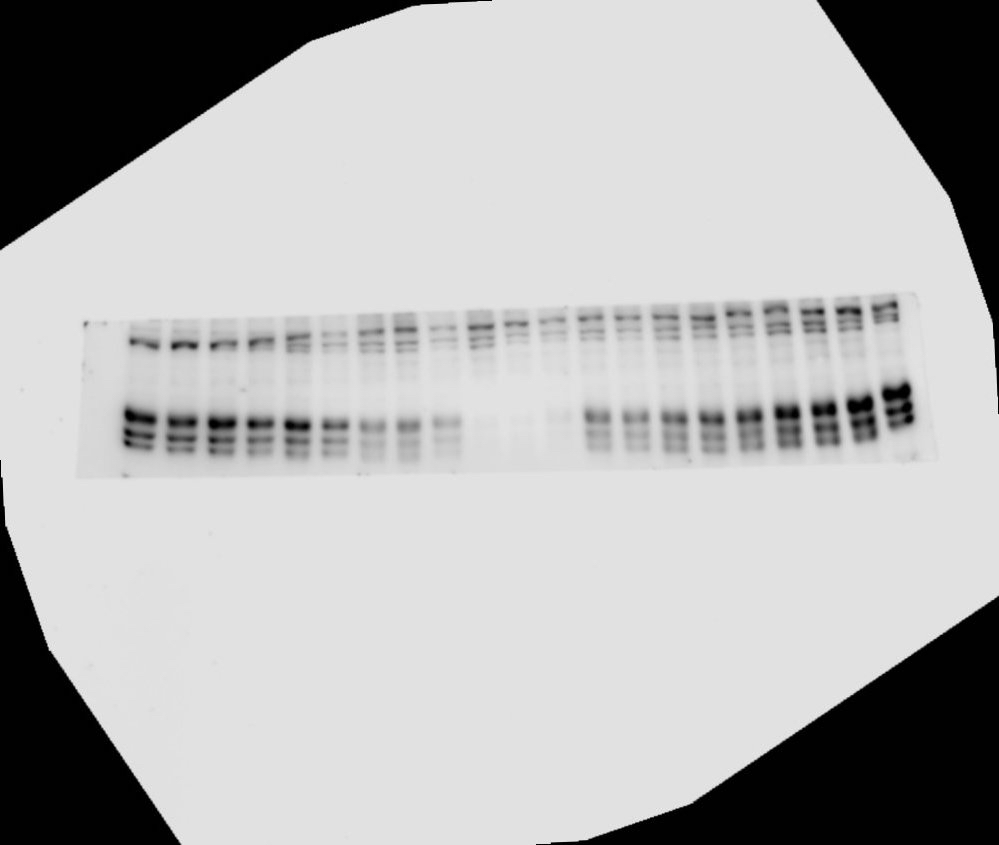

Supplement: Figure 4—figure supplement 1—source data 1. [file elife-97577-fig4-figsupp1-data1.zip › FigureS2B_SourceData1/FigS2B_EED.jpeg]

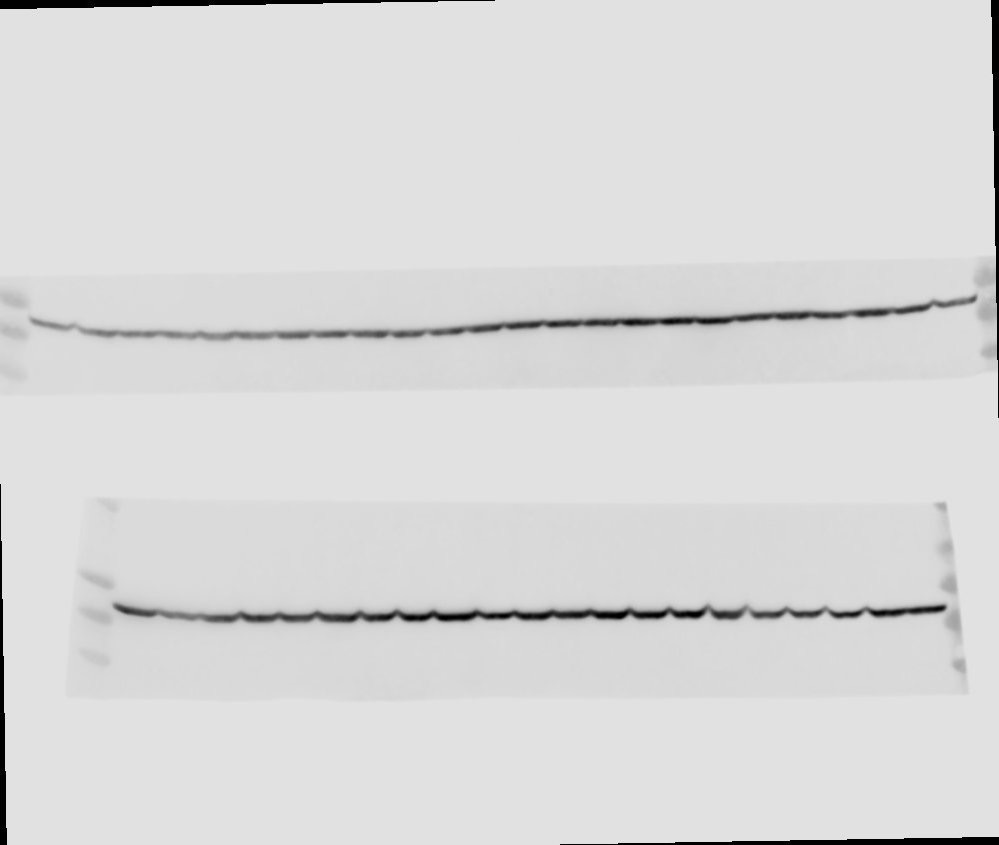

Supplement: Figure 4—figure supplement 1—source data 1. [file elife-97577-fig4-figsupp1-data1.zip › FigureS2B_SourceData1/FigS2B_H3.jpeg]

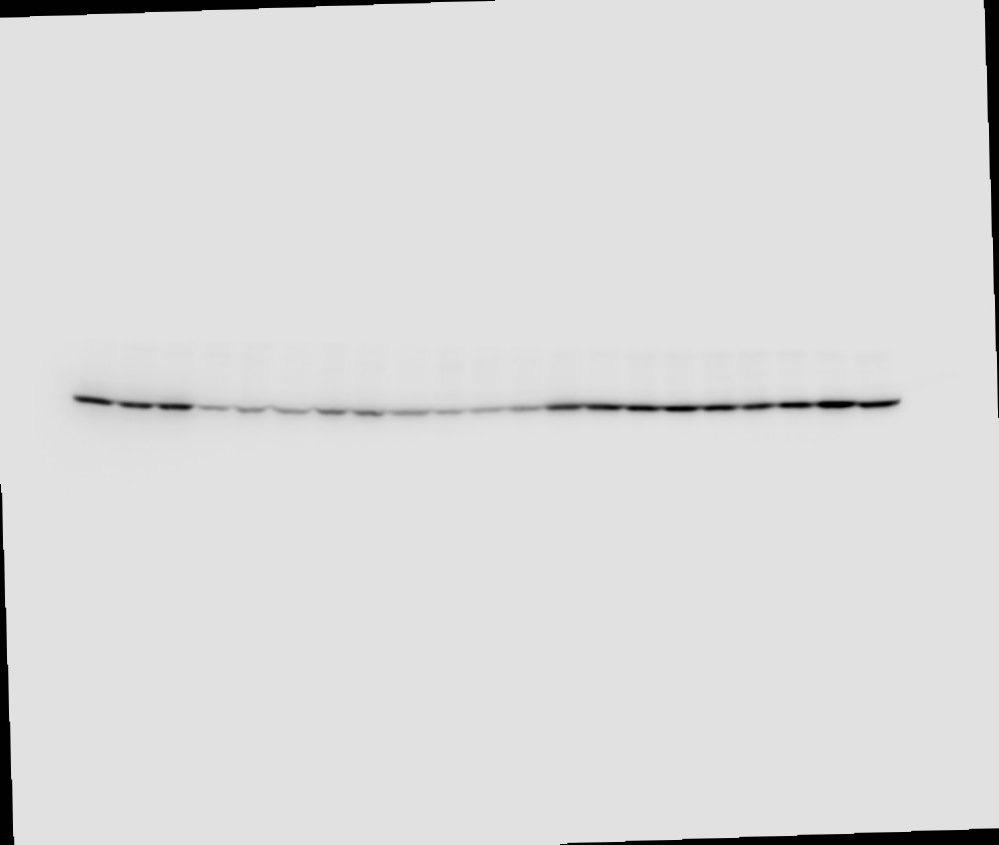

Supplement: Figure 4—figure supplement 1—source data 1. [file elife-97577-fig4-figsupp1-data1.zip › FigureS2B_SourceData1/FigS2B_H3K27me3.jpeg]

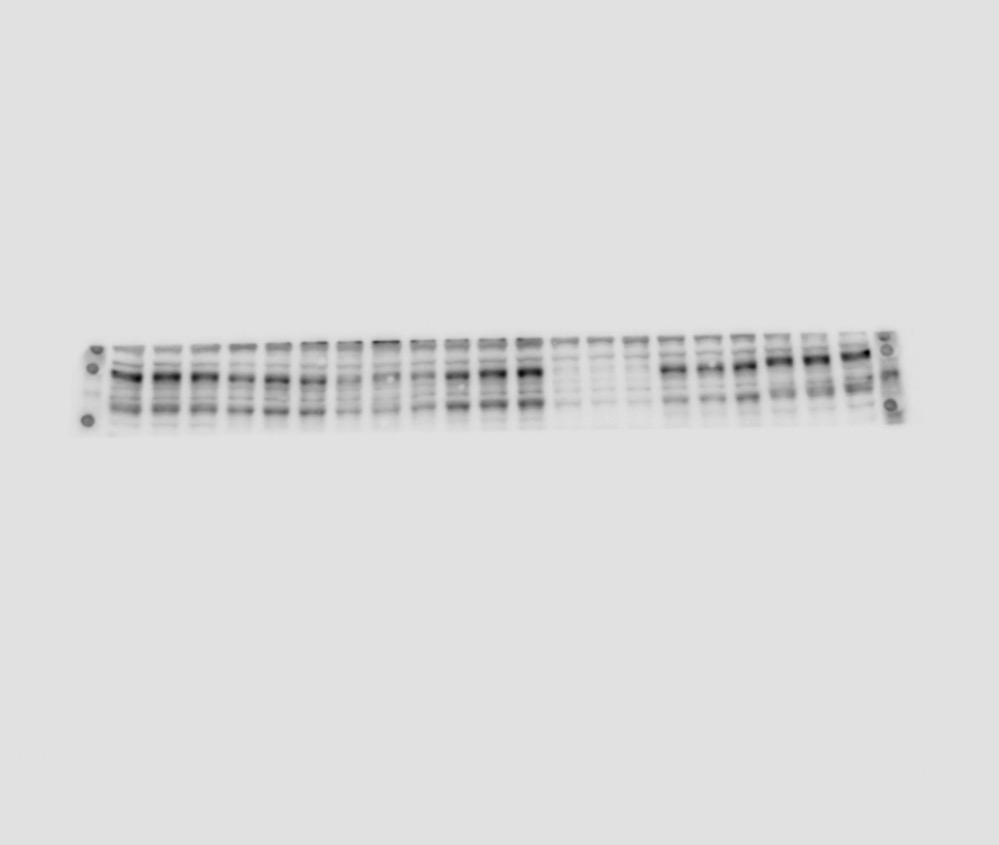

Supplement: Figure 4—figure supplement 1—source data 1. [file elife-97577-fig4-figsupp1-data1.zip › FigureS2B_SourceData1/FigS2B_MTF2.jpeg]

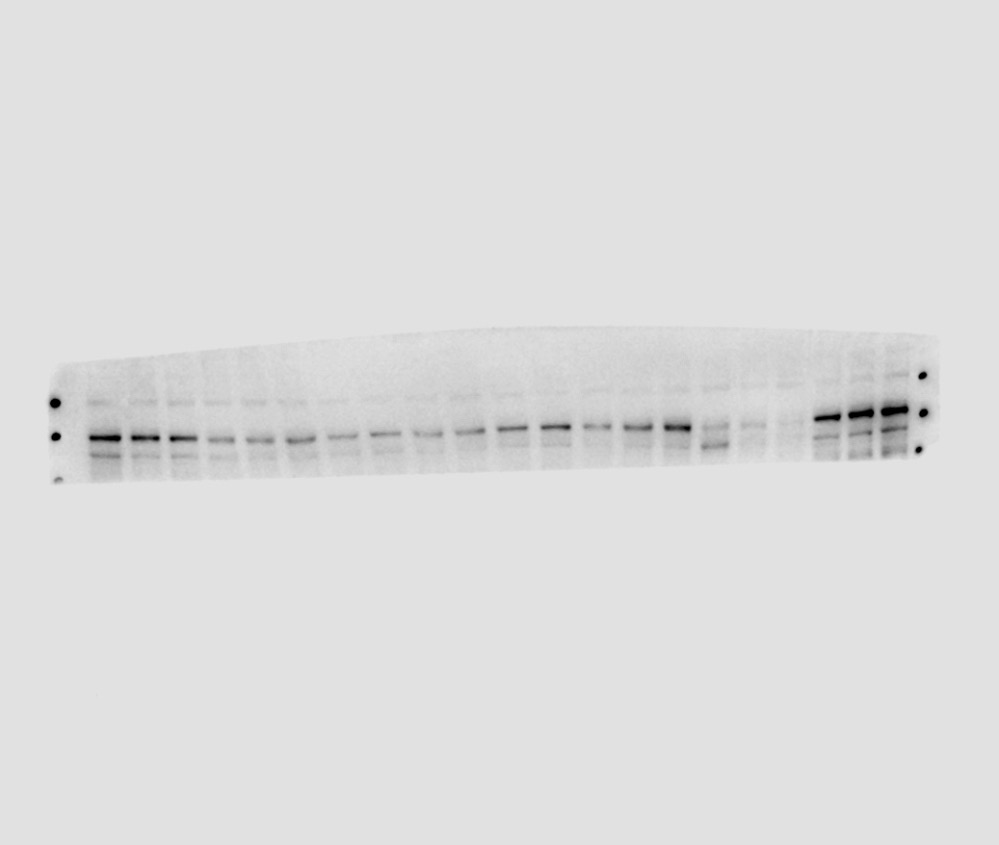

Supplement: Figure 4—figure supplement 1—source data 1. [file elife-97577-fig4-figsupp1-data1.zip › FigureS2B_SourceData1/FigS2B_JARID2.jpeg]

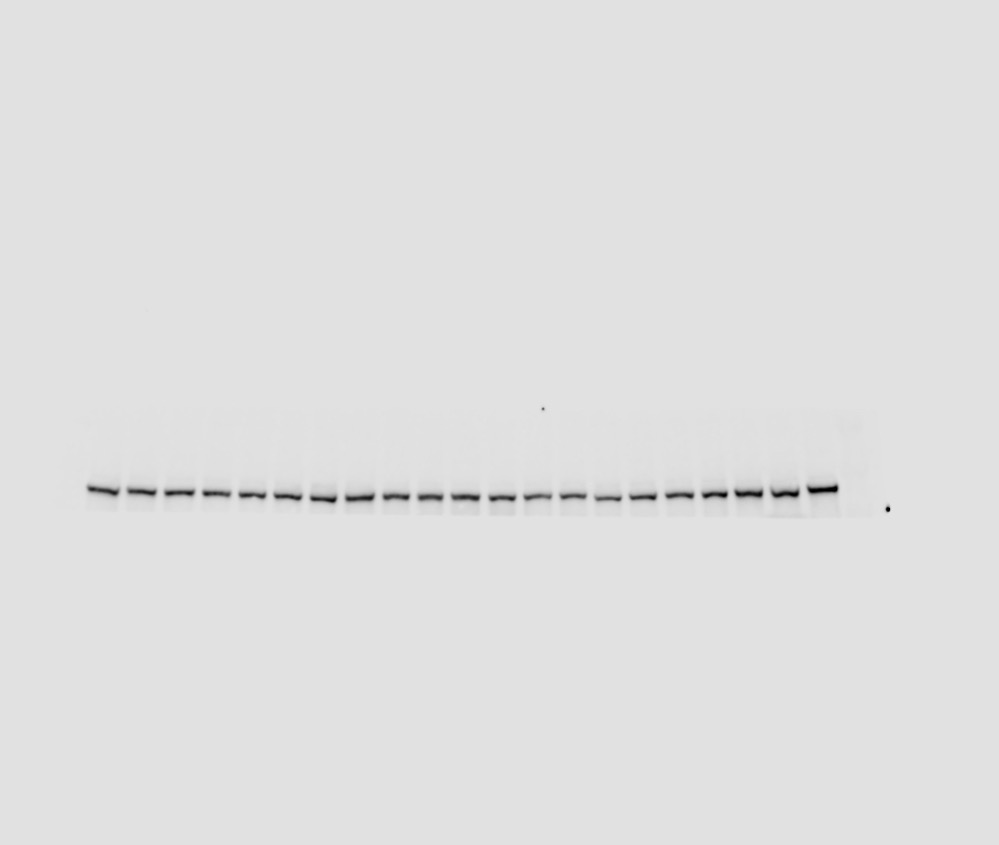

Supplement: Figure 4—figure supplement 1—source data 1. [file elife-97577-fig4-figsupp1-data1.zip › FigureS2B_SourceData1/FigS2B_Vinc.jpeg]

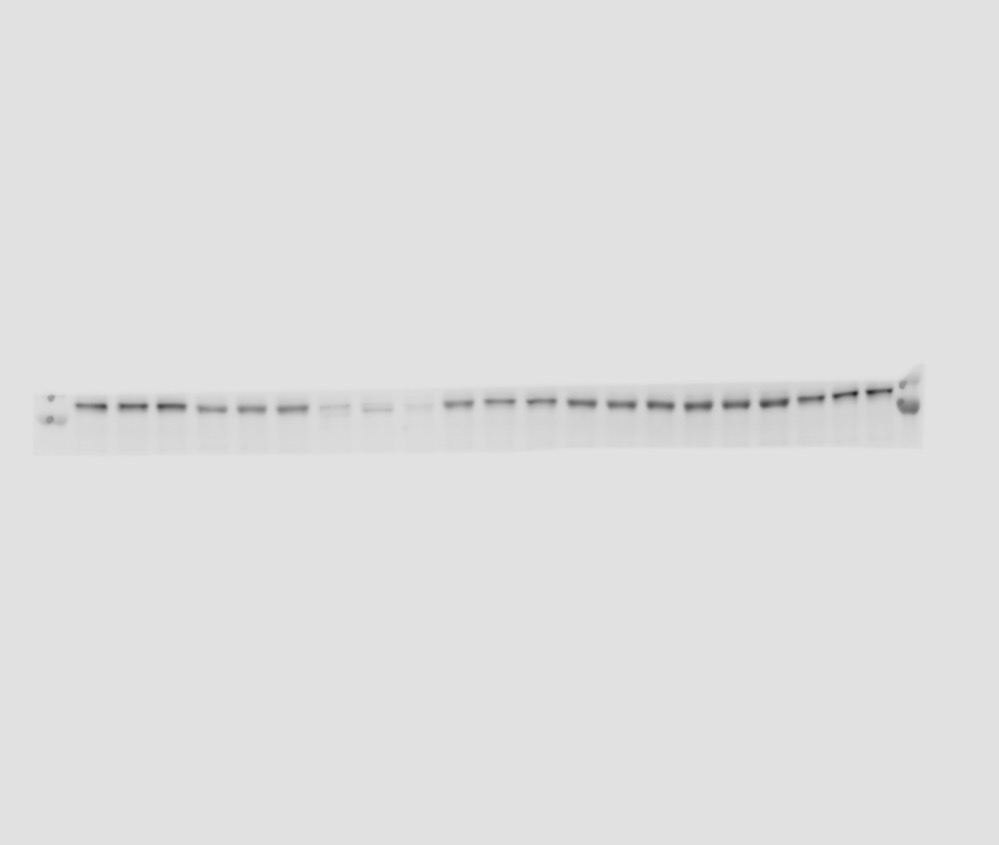

Supplement: Figure 4—figure supplement 1—source data 1. [file elife-97577-fig4-figsupp1-data1.zip › FigureS2B_SourceData1/FigS2B_SUZ12.jpeg]

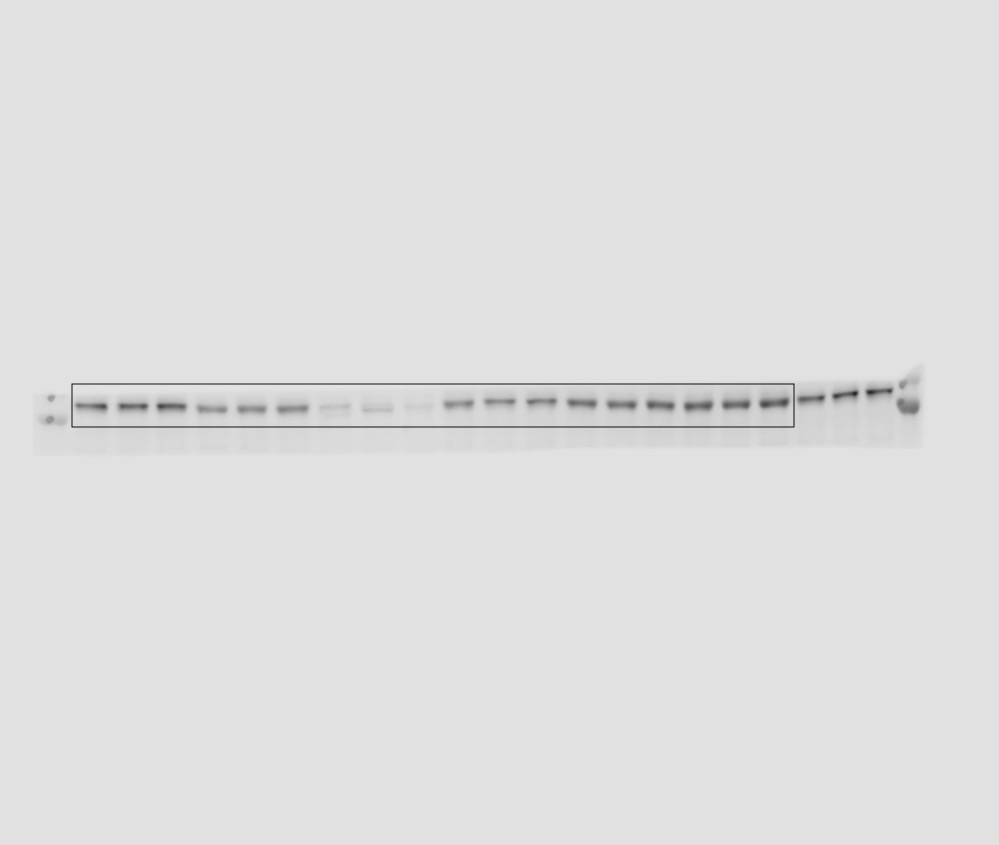

Supplement: Figure 4—figure supplement 1—source data 2. [file elife-97577-fig4-figsupp1-data2.zip › FigureS2B_SourceData2/FigS2B_Box_SUZ12.jpeg]

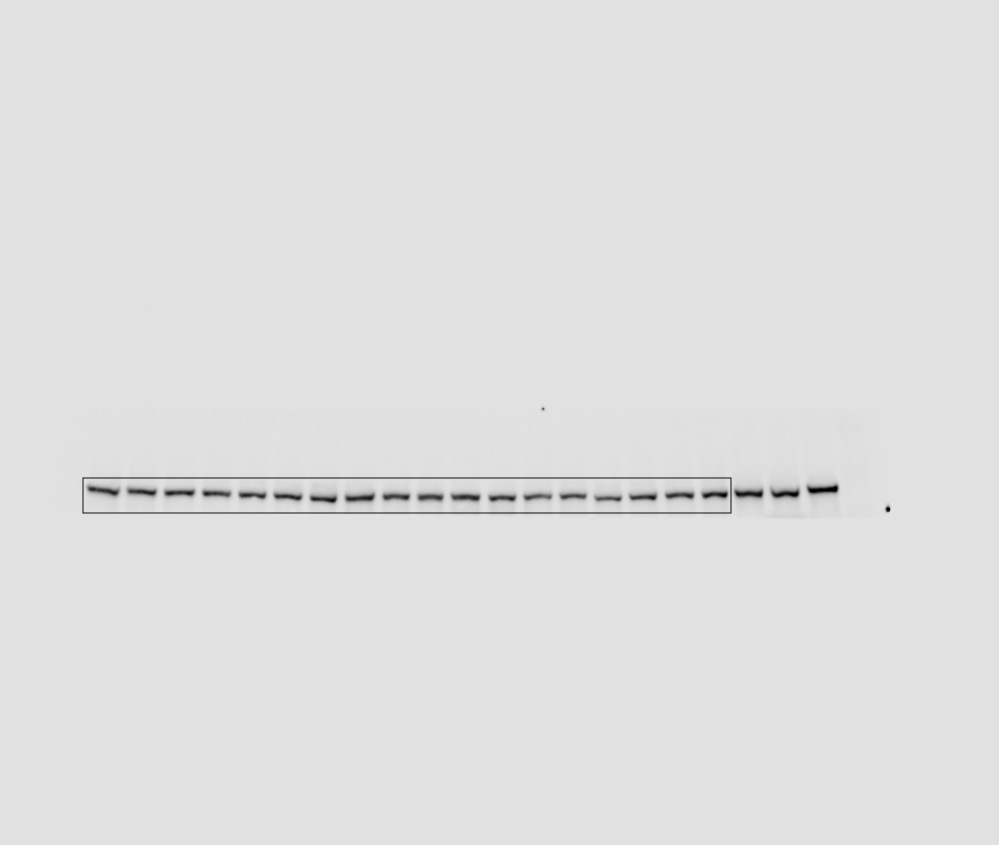

Supplement: Figure 4—figure supplement 1—source data 2. [file elife-97577-fig4-figsupp1-data2.zip › FigureS2B_SourceData2/FigS2B_Box_Vinc.jpeg]

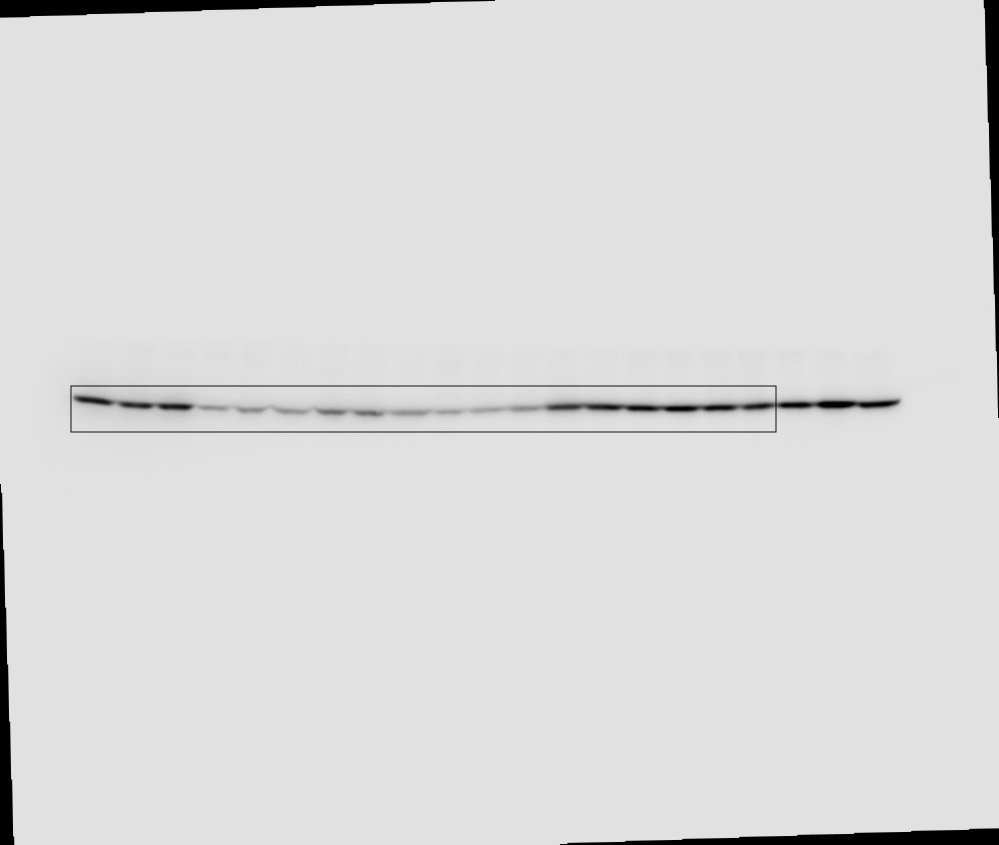

Supplement: Figure 4—figure supplement 1—source data 2. [file elife-97577-fig4-figsupp1-data2.zip › FigureS2B_SourceData2/FigS2B_Box_H3K27me3.jpeg]

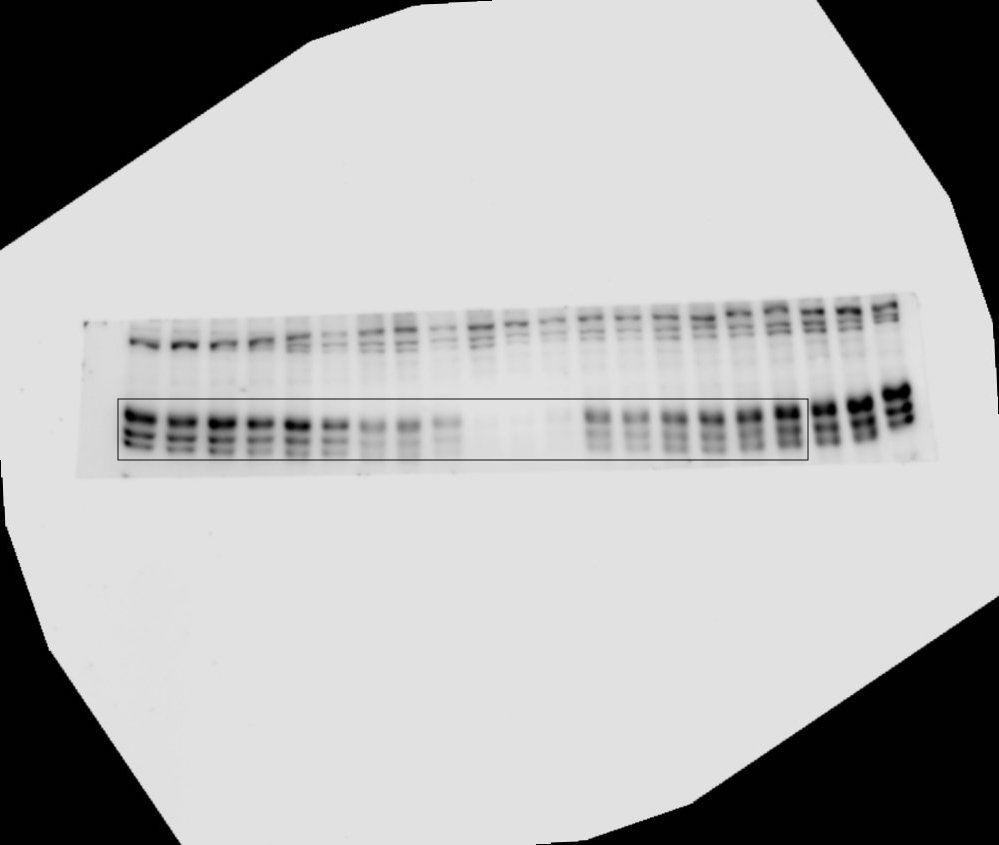

Supplement: Figure 4—figure supplement 1—source data 2. [file elife-97577-fig4-figsupp1-data2.zip › FigureS2B_SourceData2/FigS2B_Box_EED.jpeg]

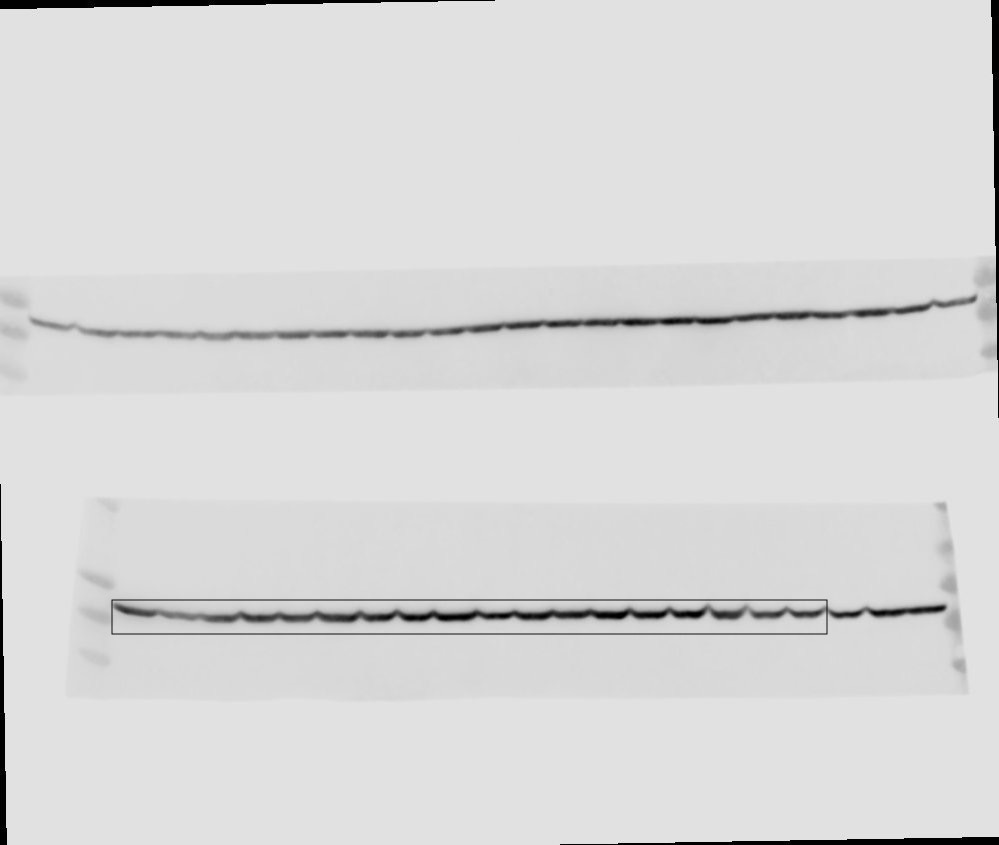

Supplement: Figure 4—figure supplement 1—source data 2. [file elife-97577-fig4-figsupp1-data2.zip › FigureS2B_SourceData2/FigS2B_Box_H3.jpeg]

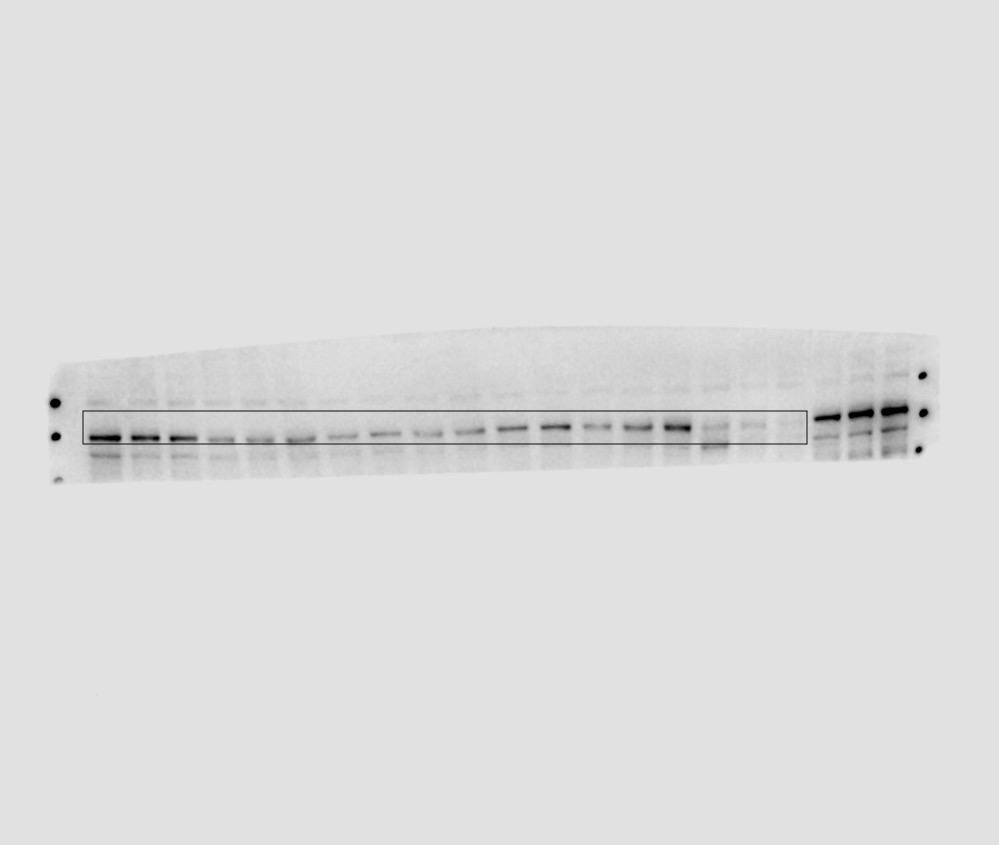

Supplement: Figure 4—figure supplement 1—source data 2. [file elife-97577-fig4-figsupp1-data2.zip › FigureS2B_SourceData2/FigS2B_Box_JARID2.jpeg]

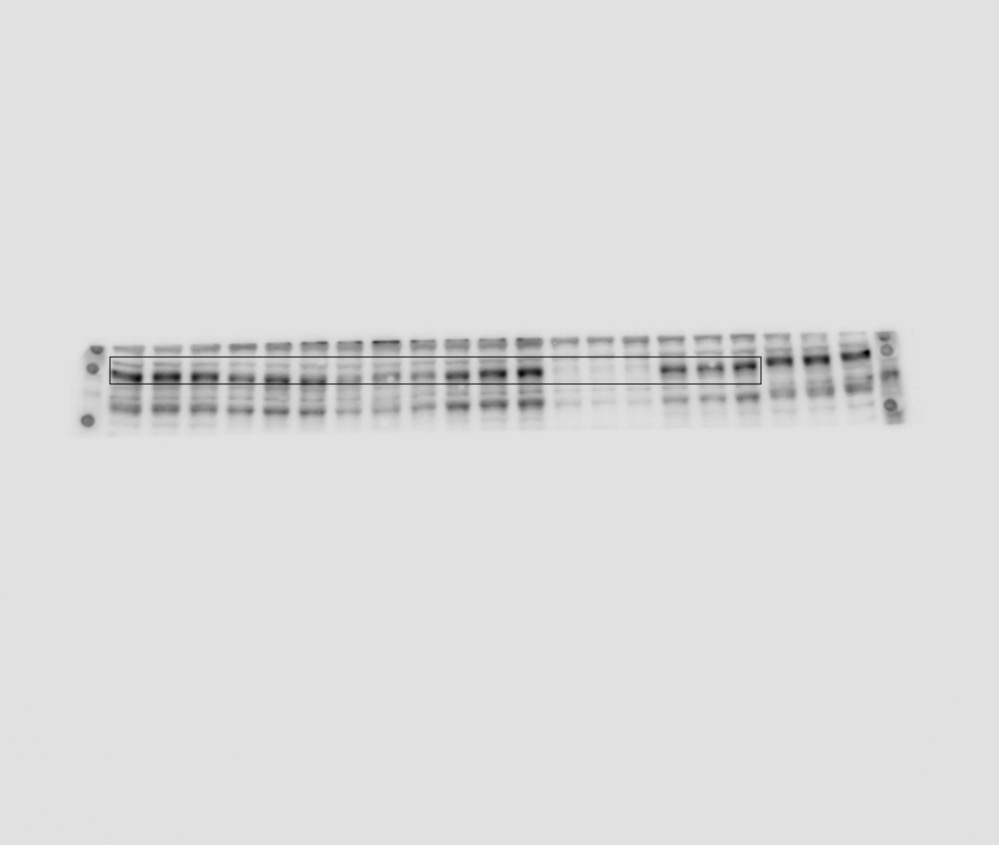

Supplement: Figure 4—figure supplement 1—source data 2. [file elife-97577-fig4-figsupp1-data2.zip › FigureS2B_SourceData2/FigS2B_Box_MTF2.jpeg]

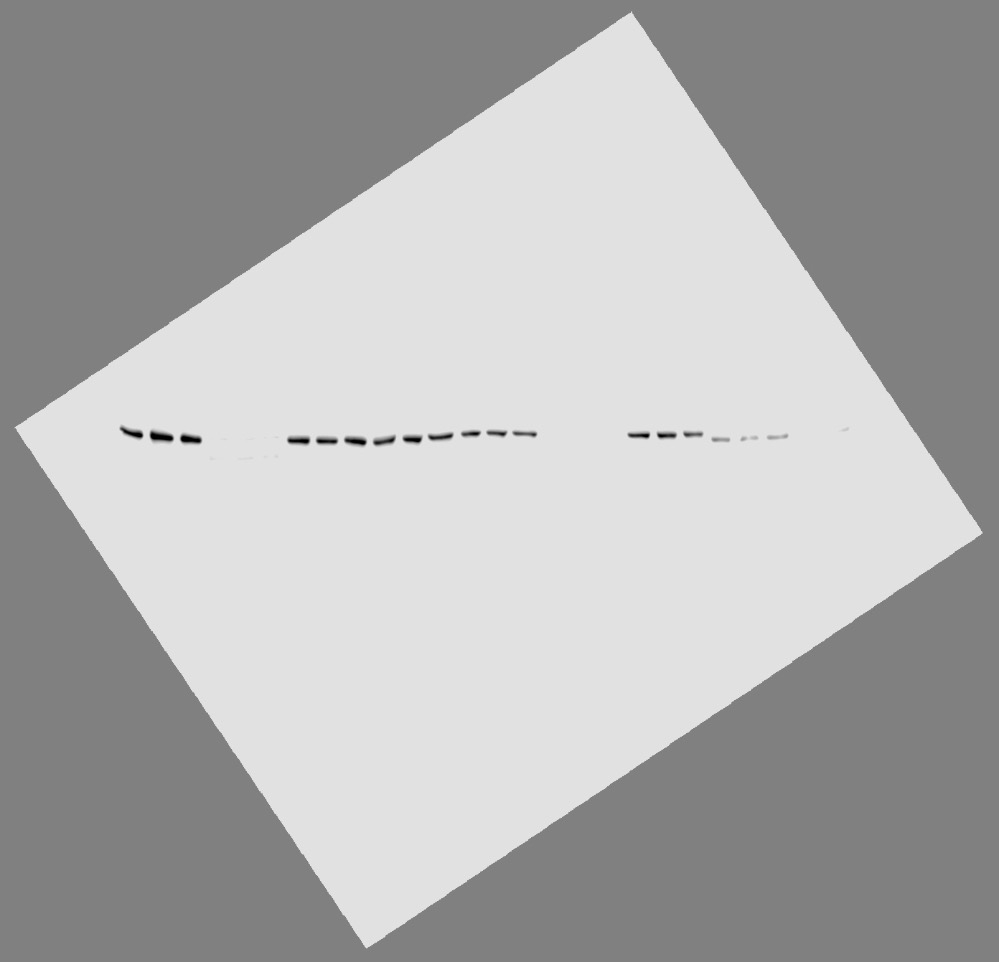

Supplement: Figure 4—figure supplement 3—source data 1. [file elife-97577-fig4-figsupp3-data1.zip › FigureS2D_SourceData1/FigS2D_SUZ12.jpeg]

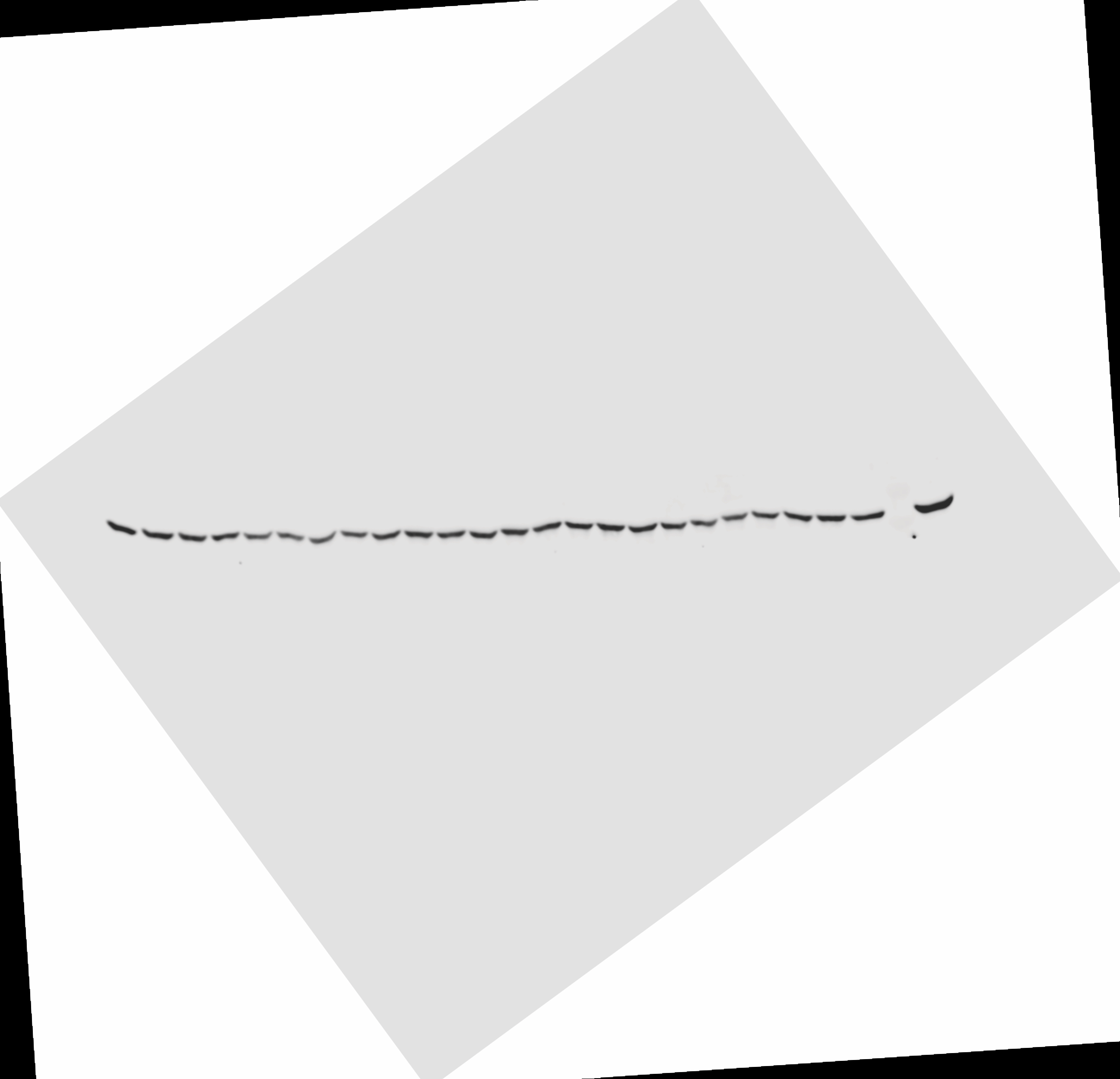

Supplement: Figure 4—figure supplement 3—source data 1. [file elife-97577-fig4-figsupp3-data1.zip › FigureS2D_SourceData1/FigS2D_Actin.jpeg]

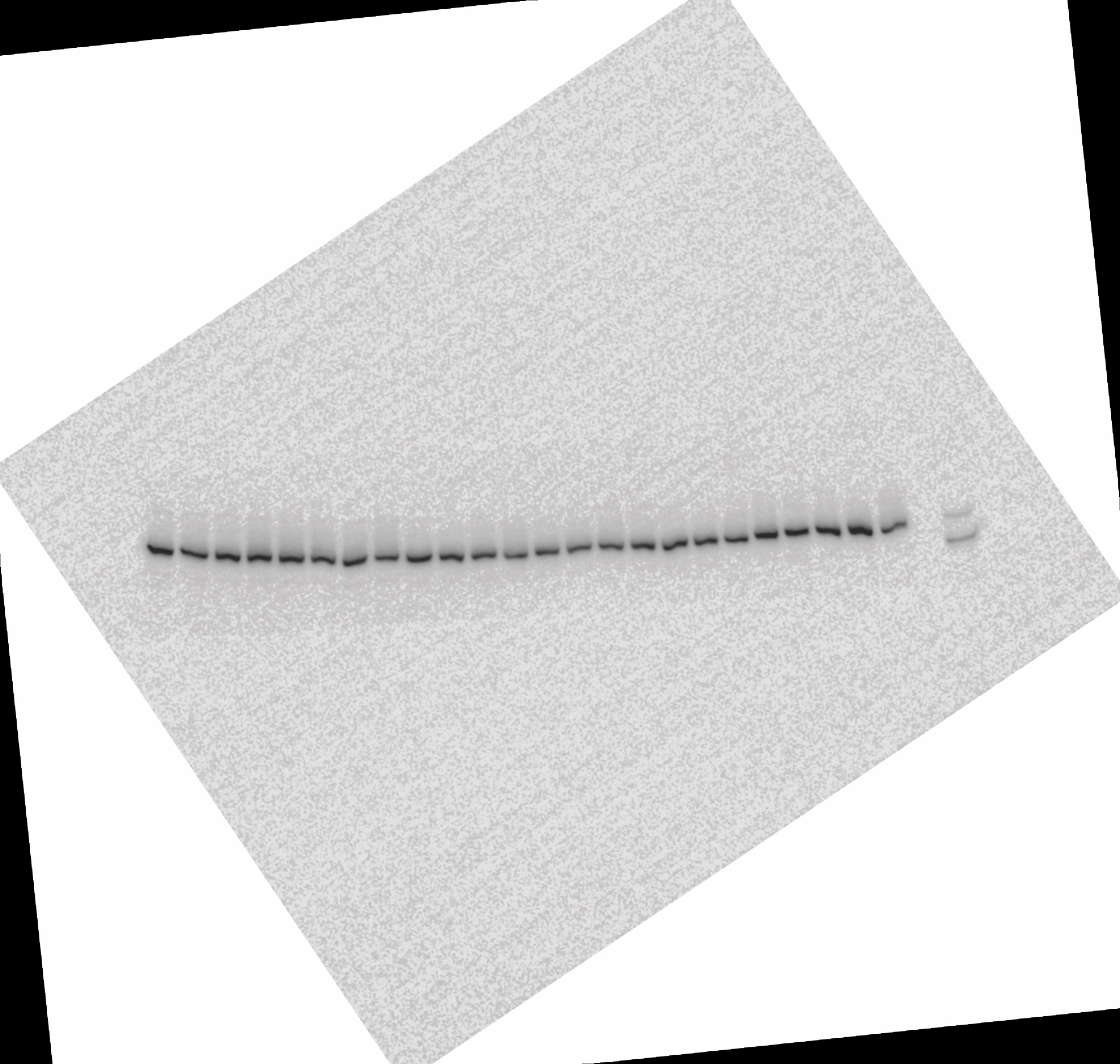

Supplement: Figure 4—figure supplement 3—source data 1. [file elife-97577-fig4-figsupp3-data1.zip › FigureS2D_SourceData1/FigS2D_PARP.jpeg]

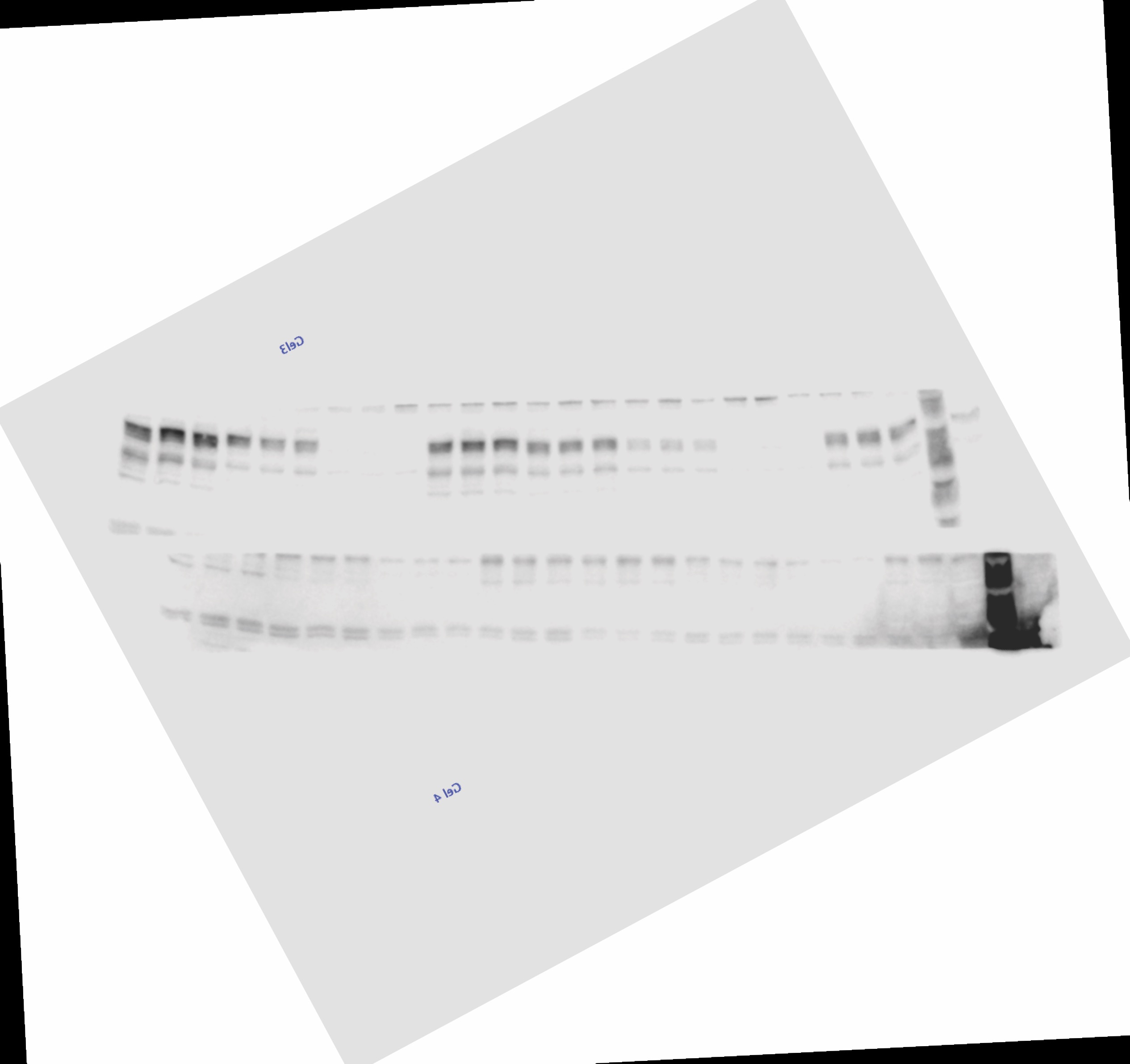

Supplement: Figure 4—figure supplement 3—source data 1. [file elife-97577-fig4-figsupp3-data1.zip › FigureS2D_SourceData1/FigS2D_MTF2.jpeg]

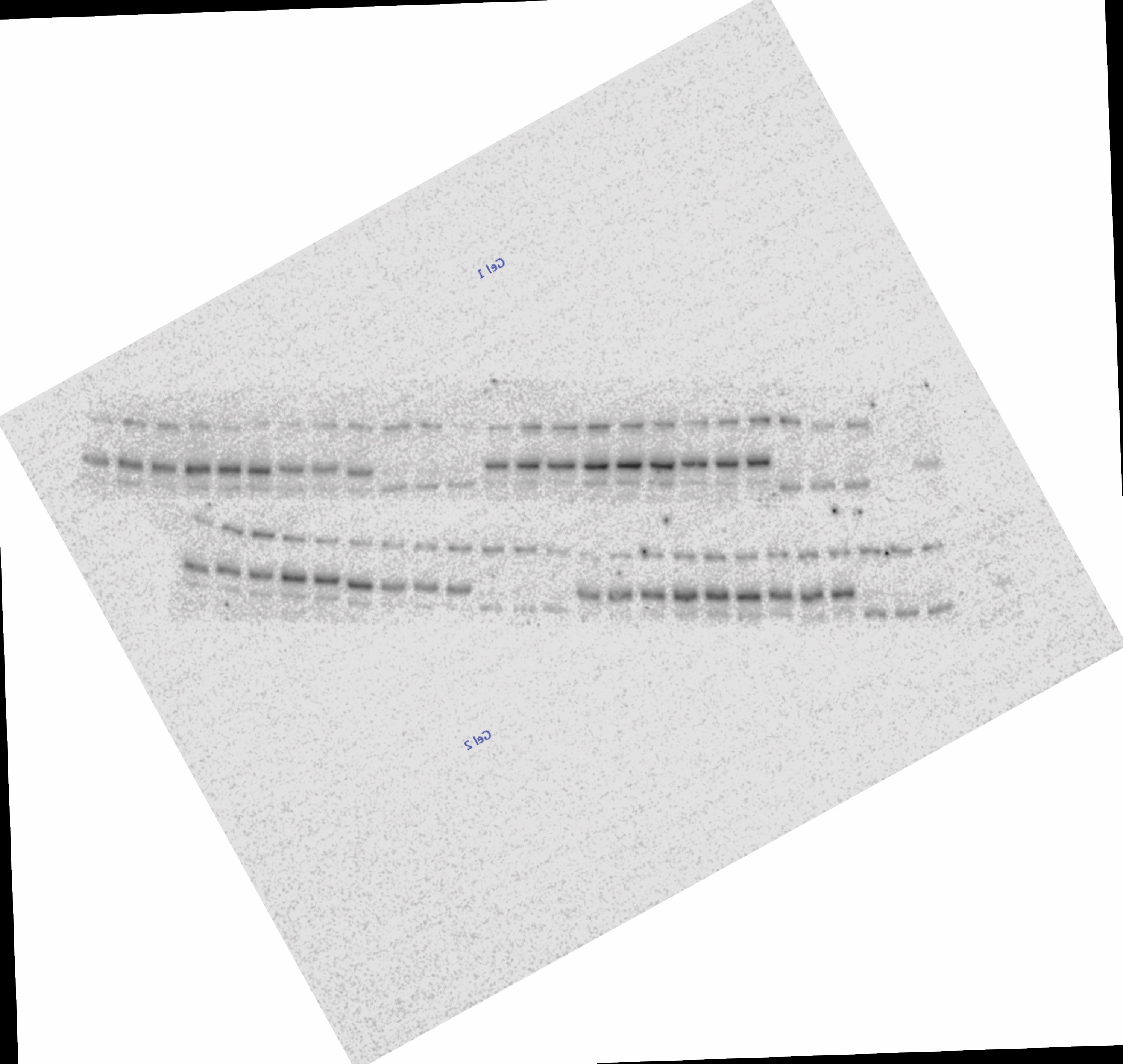

Supplement: Figure 4—figure supplement 3—source data 1. [file elife-97577-fig4-figsupp3-data1.zip › FigureS2D_SourceData1/FigS2D_JARID2.jpeg]

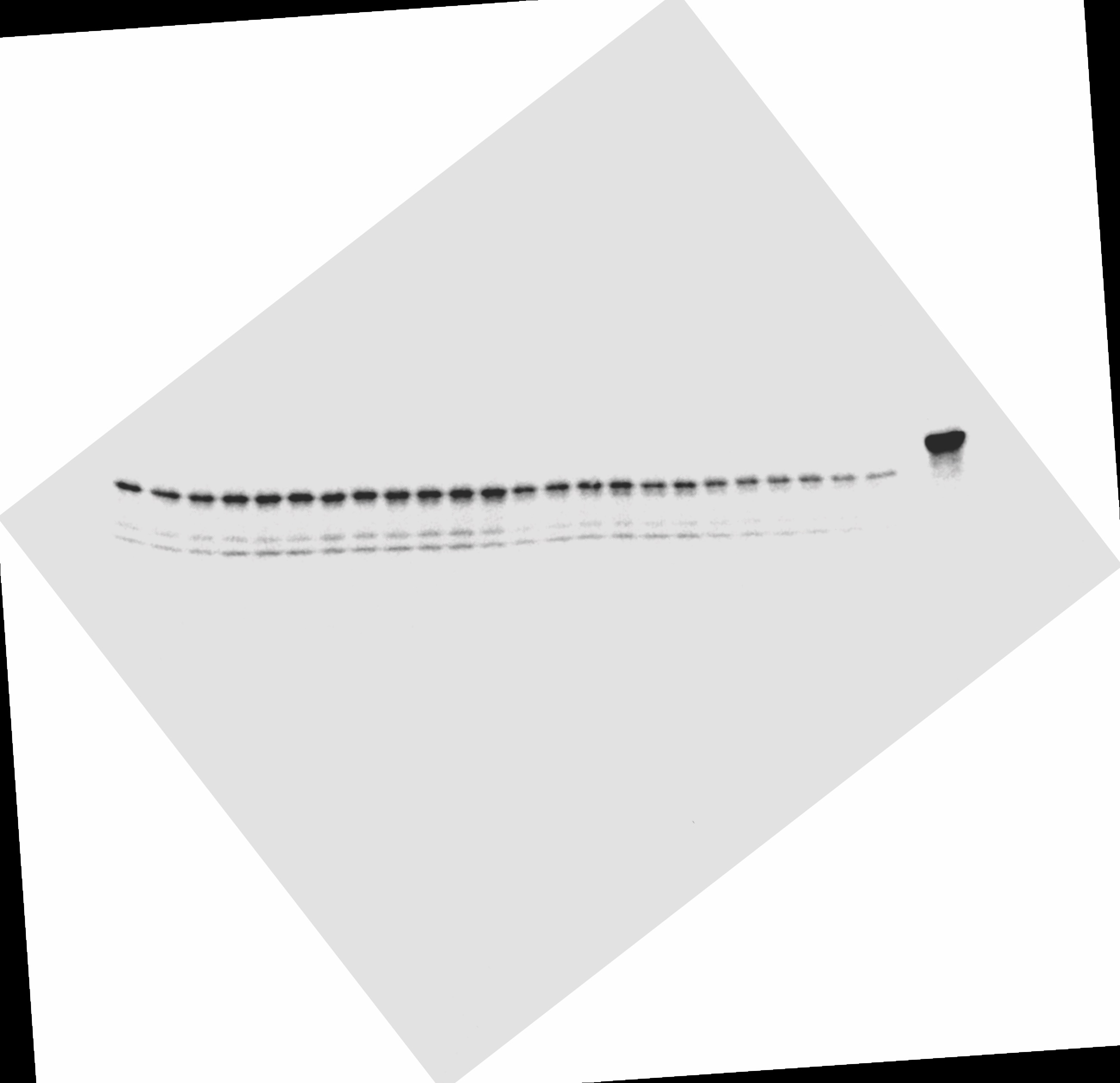

Supplement: Figure 4—figure supplement 3—source data 1. [file elife-97577-fig4-figsupp3-data1.zip › FigureS2D_SourceData1/FigS2D_BIM.jpeg]

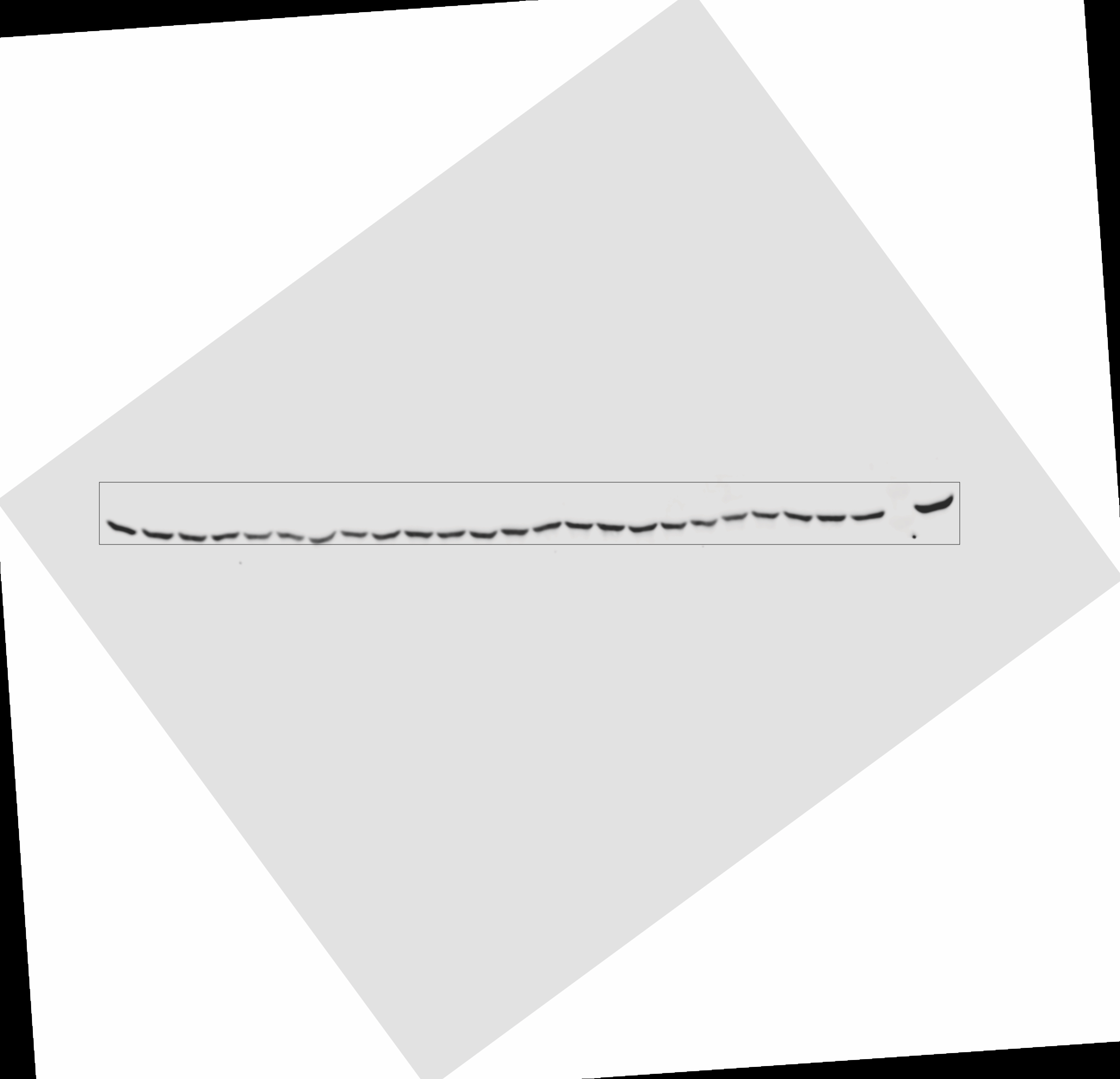

Supplement: Figure 4—figure supplement 3—source data 2. [file elife-97577-fig4-figsupp3-data2.zip › FigureS2D_SourceData2/FigS2D_Box_Actin.jpeg]

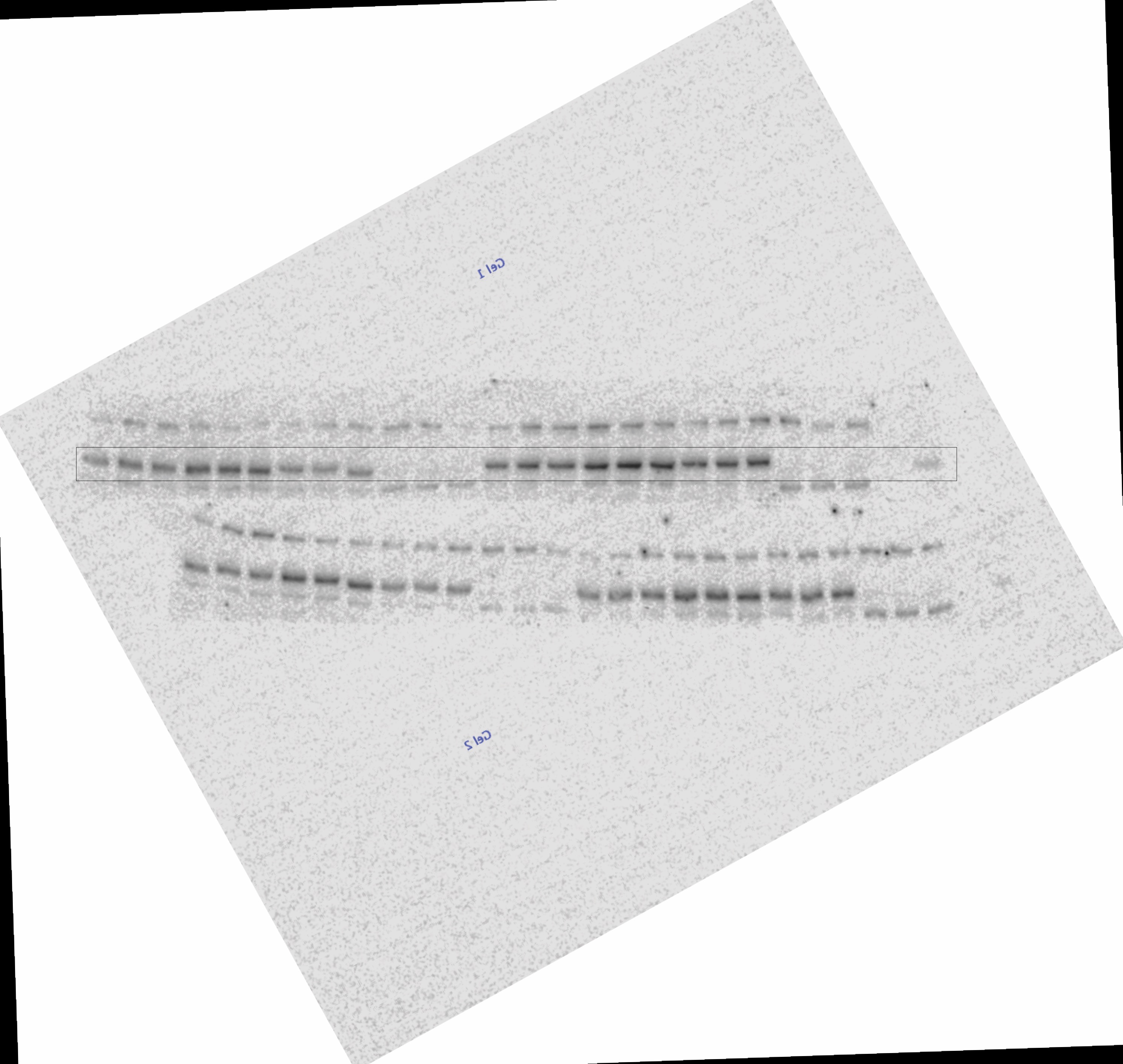

Supplement: Figure 4—figure supplement 3—source data 2. [file elife-97577-fig4-figsupp3-data2.zip › FigureS2D_SourceData2/FigS2D_Box_JARID2.jpeg]

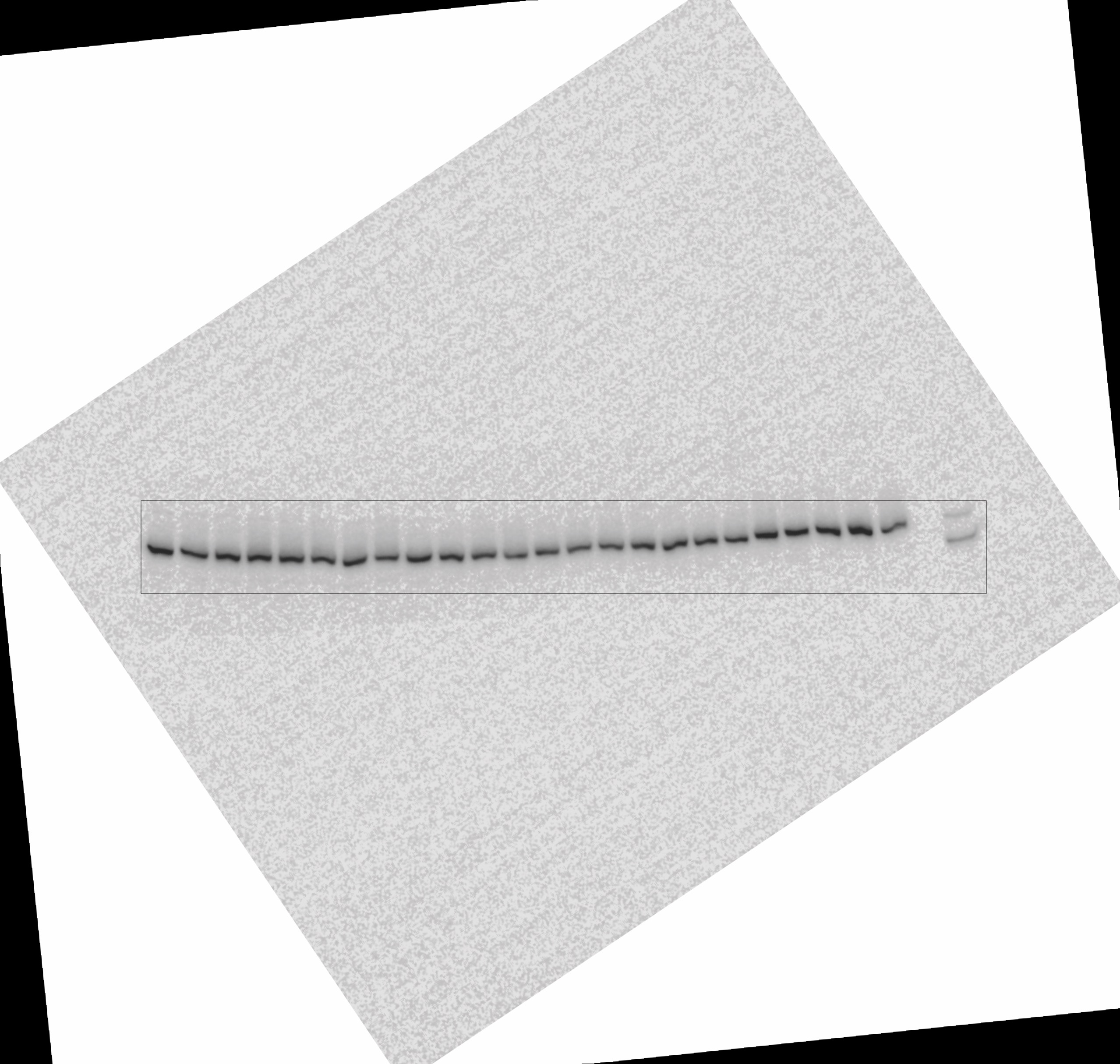

Supplement: Figure 4—figure supplement 3—source data 2. [file elife-97577-fig4-figsupp3-data2.zip › FigureS2D_SourceData2/FigS2D_Box_PARP.jpeg]

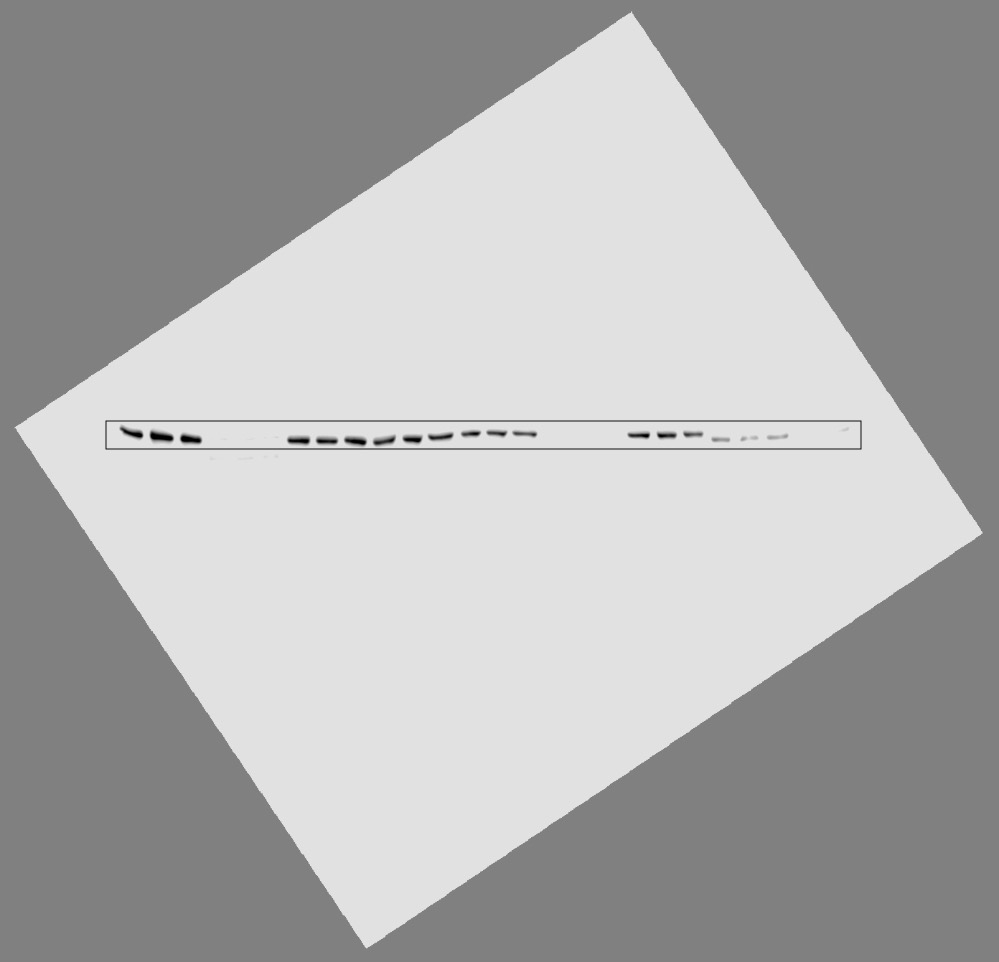

Supplement: Figure 4—figure supplement 3—source data 2. [file elife-97577-fig4-figsupp3-data2.zip › FigureS2D_SourceData2/FigS2D_Box_SUZ12.jpeg]

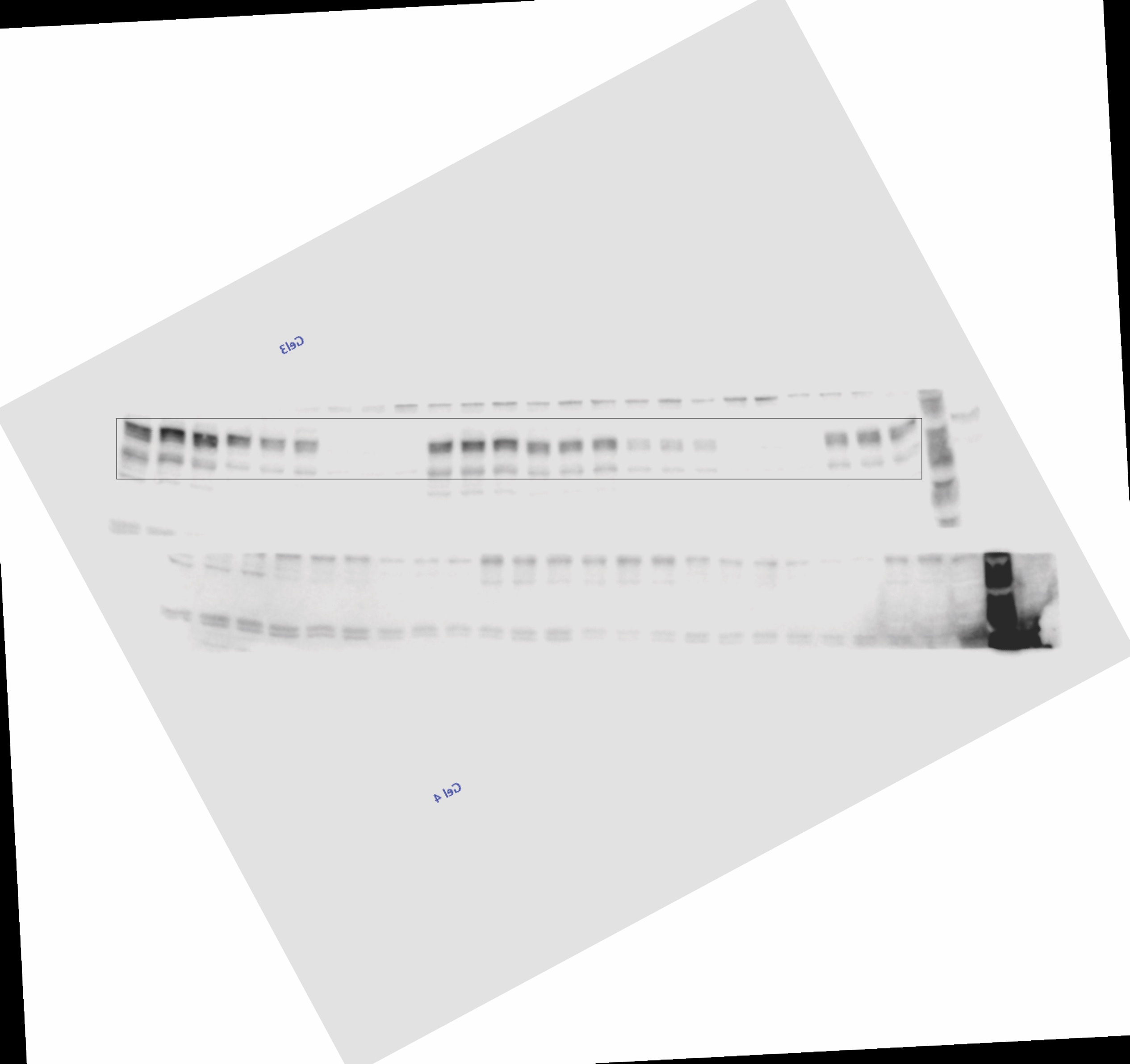

Supplement: Figure 4—figure supplement 3—source data 2. [file elife-97577-fig4-figsupp3-data2.zip › FigureS2D_SourceData2/FigS2D_Box_MTF2.jpeg]

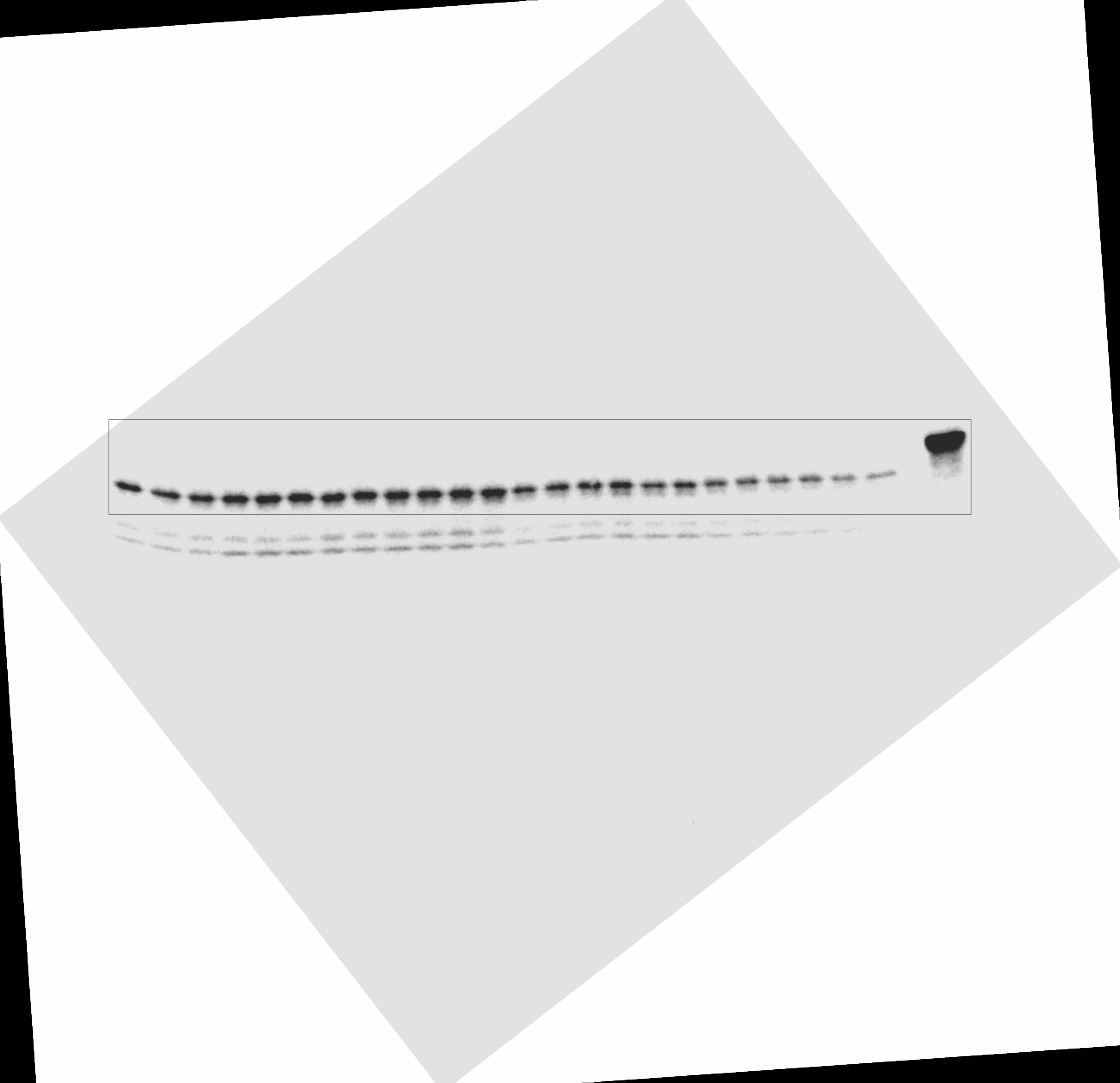

Supplement: Figure 4—figure supplement 3—source data 2. [file elife-97577-fig4-figsupp3-data2.zip › FigureS2D_SourceData2/FigS2D_Box_BIM.jpeg]

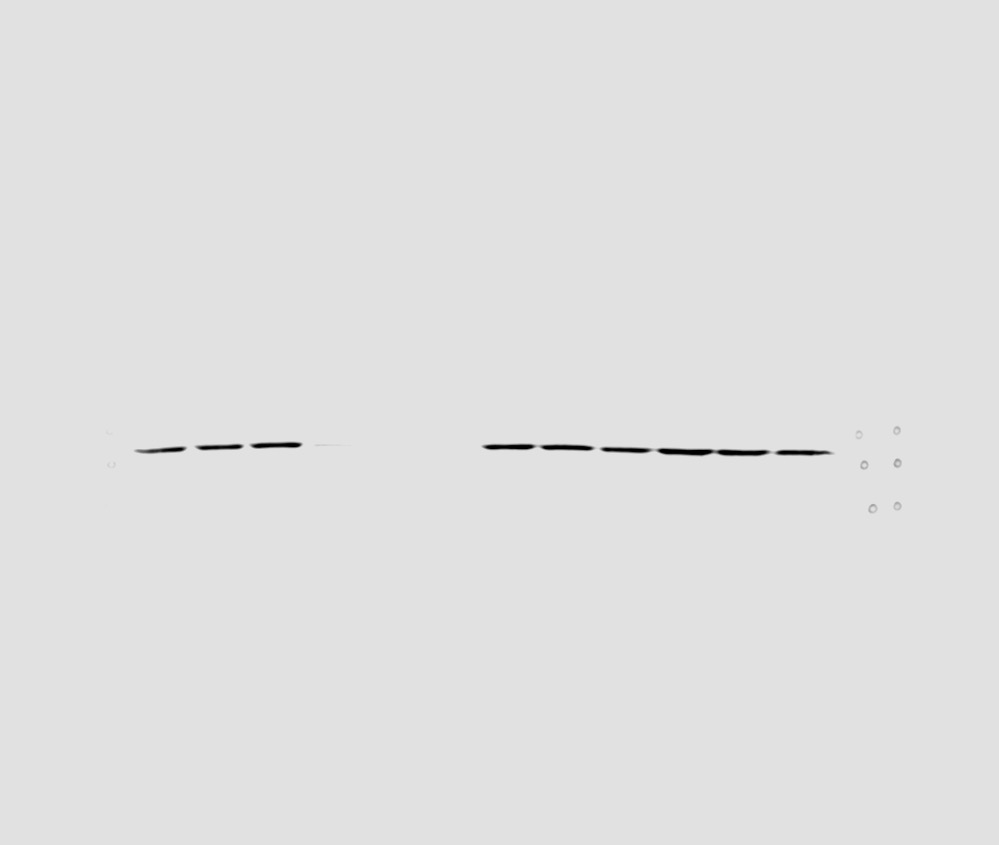

Supplement: Figure 5—source data 1. [file elife-97577-fig5-data1.zip › Figure5A_SourceData1/Fig5A_H3K27me3.jpeg]

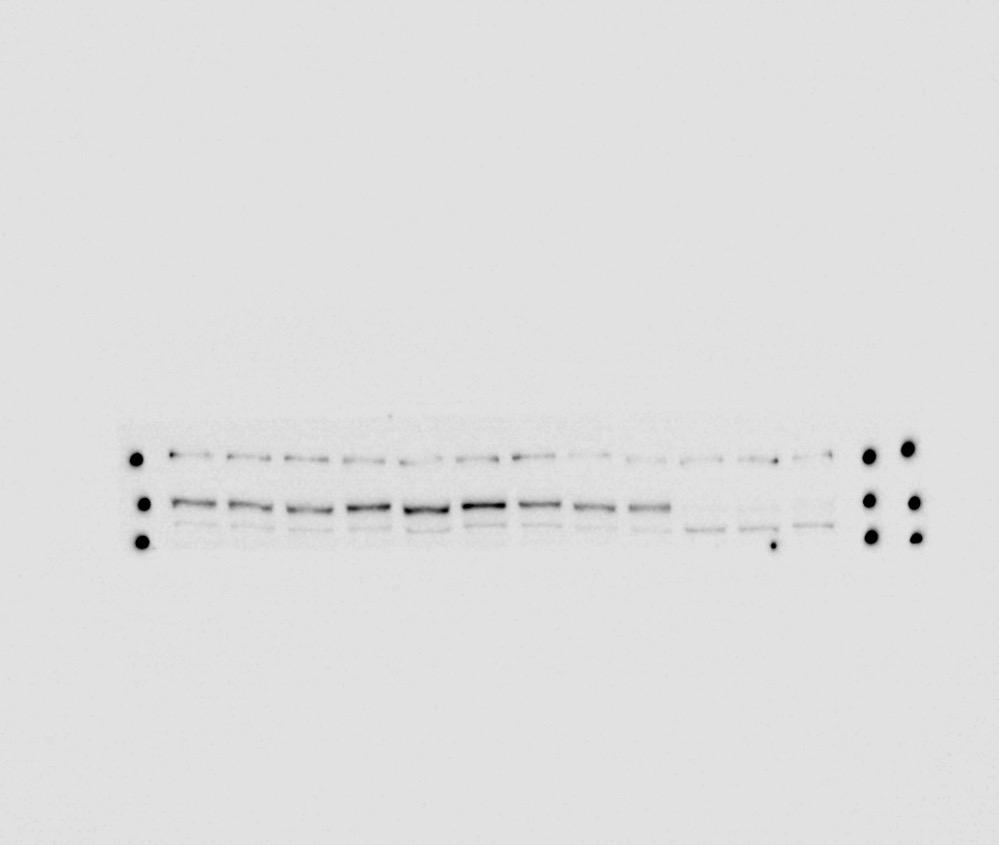

Supplement: Figure 5—source data 1. [file elife-97577-fig5-data1.zip › Figure5A_SourceData1/Fig5A_JARID2.jpeg]

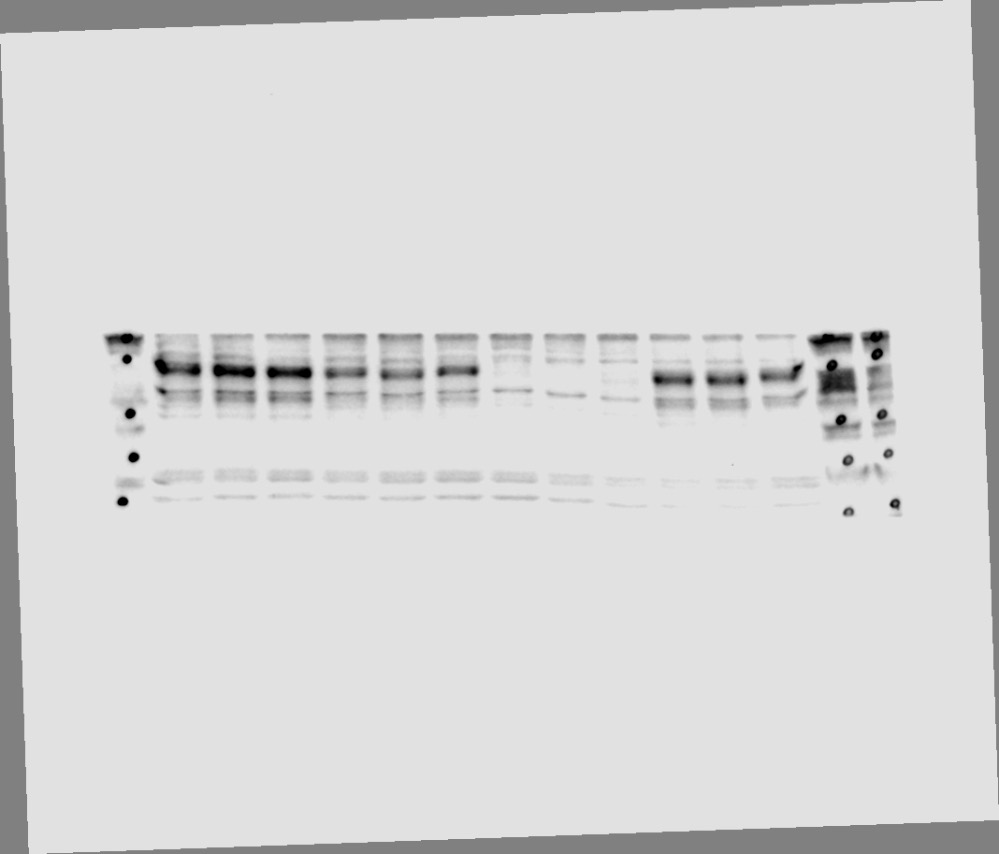

Supplement: Figure 5—source data 1. [file elife-97577-fig5-data1.zip › Figure5A_SourceData1/Fig5A_MTF2.jpeg]

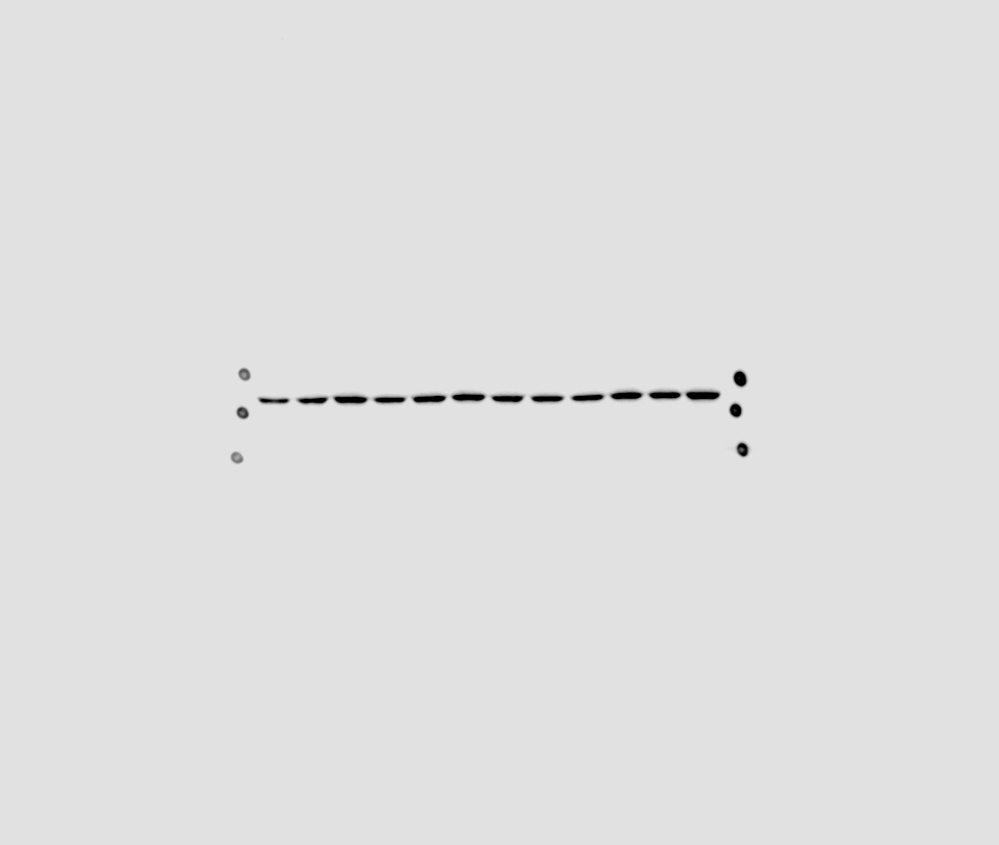

Supplement: Figure 5—source data 1. [file elife-97577-fig5-data1.zip › Figure5A_SourceData1/Fig5A_H3.jpeg]

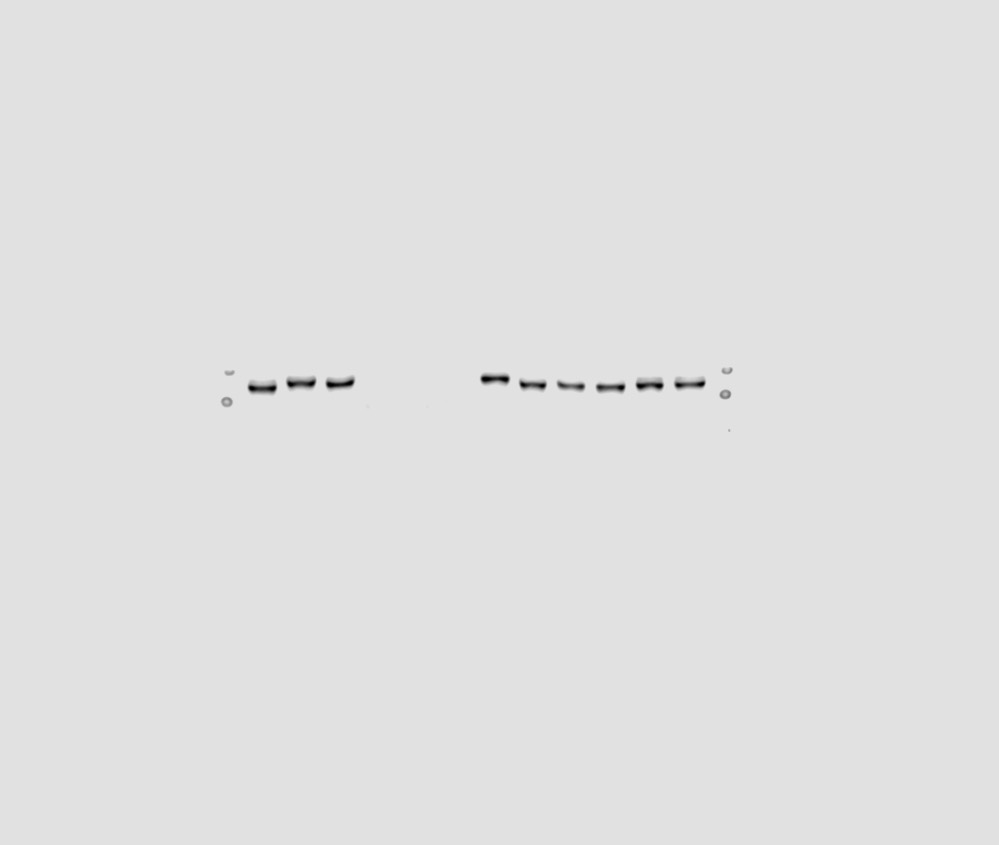

Supplement: Figure 5—source data 1. [file elife-97577-fig5-data1.zip › Figure5A_SourceData1/Fig5A_SUZ12.jpeg]

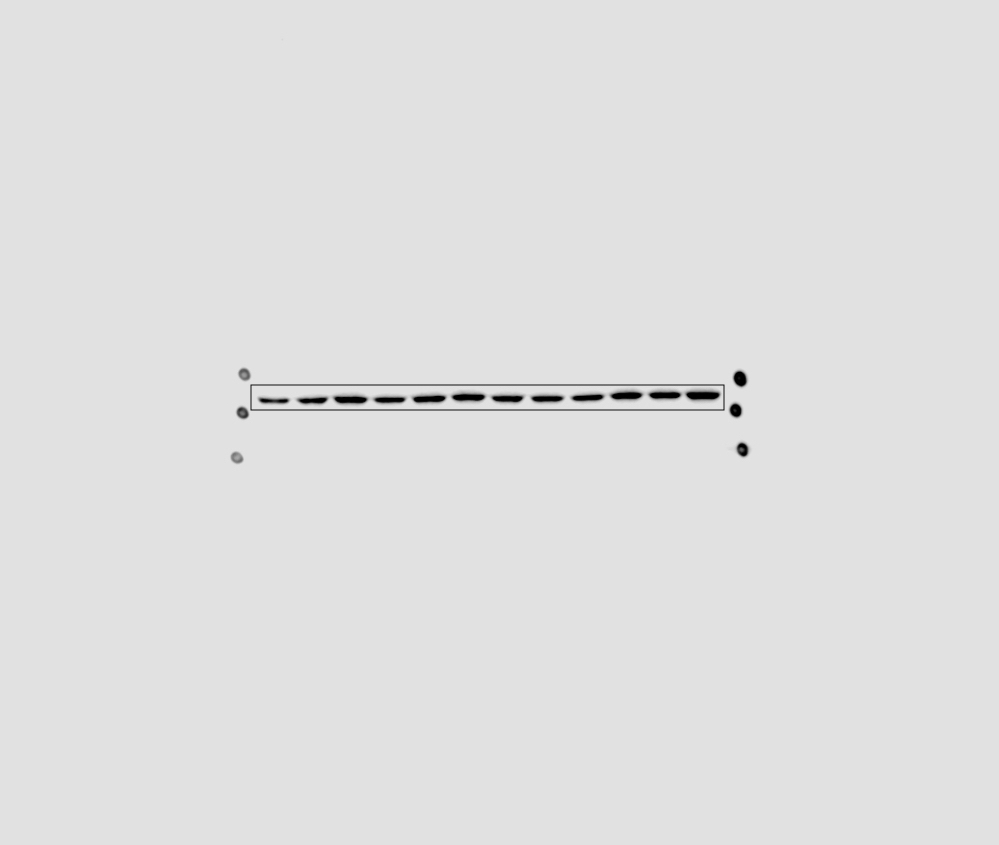

Supplement: Figure 5—source data 2. [file elife-97577-fig5-data2.zip › Figure5A_SourceData2/Fig5A_Box_H3.jpg]

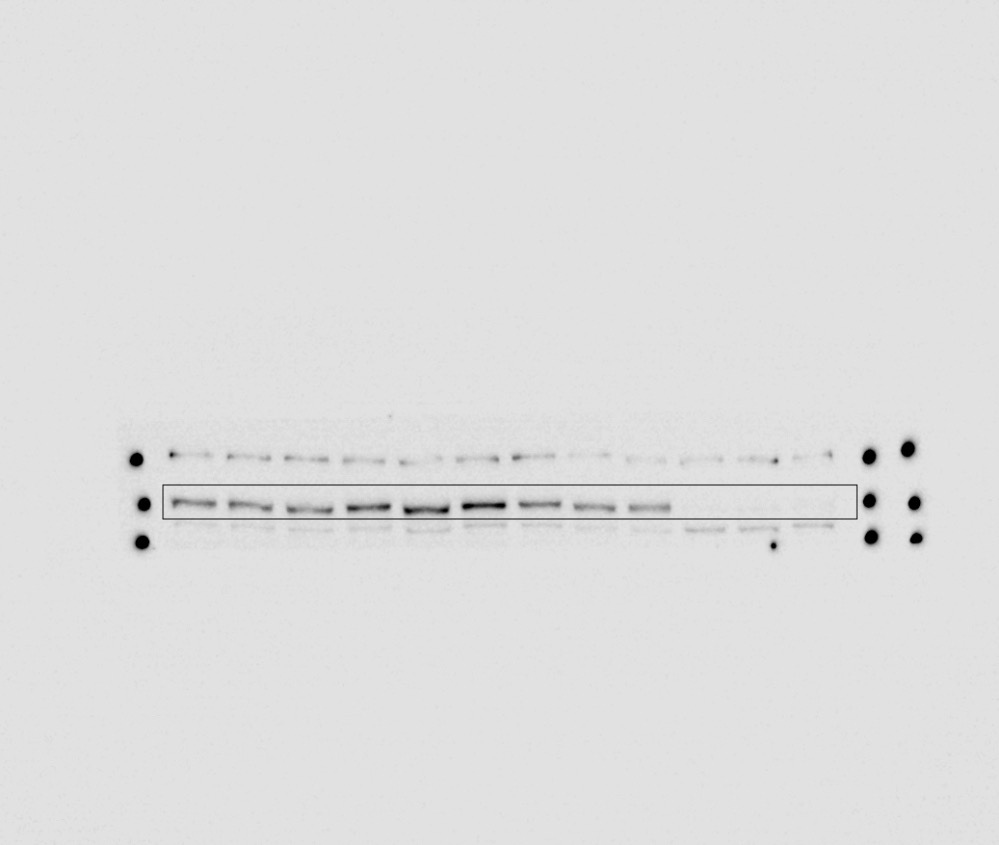

Supplement: Figure 5—source data 2. [file elife-97577-fig5-data2.zip › Figure5A_SourceData2/Fig5A_Box_JARID2.jpeg]

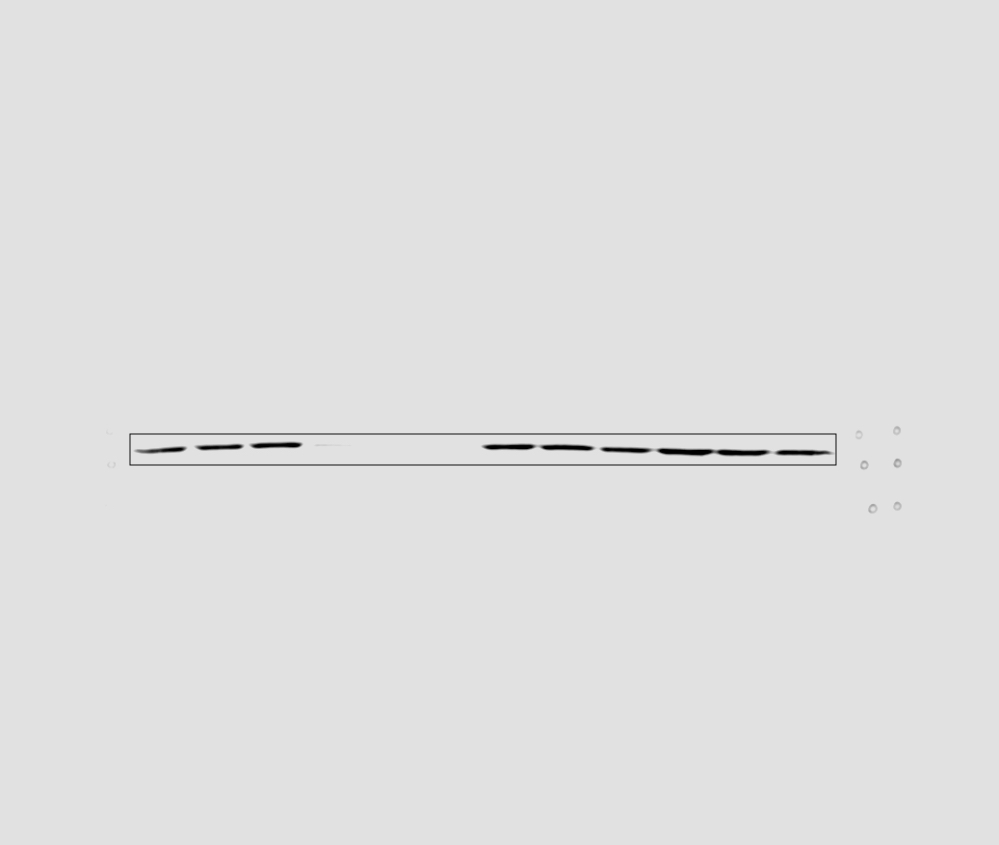

Supplement: Figure 5—source data 2. [file elife-97577-fig5-data2.zip › Figure5A_SourceData2/Fig5A_Box_H3K27me3.jpeg]

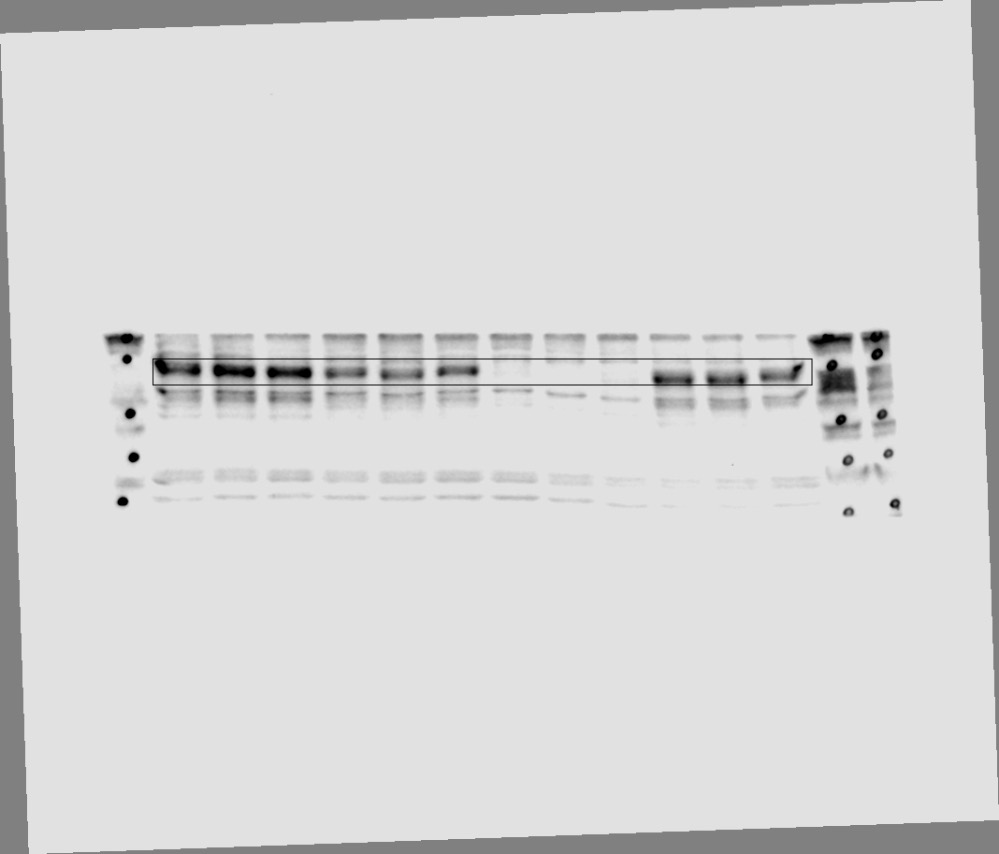

Supplement: Figure 5—source data 2. [file elife-97577-fig5-data2.zip › Figure5A_SourceData2/Fig5A_Box_MTF2.jpeg]

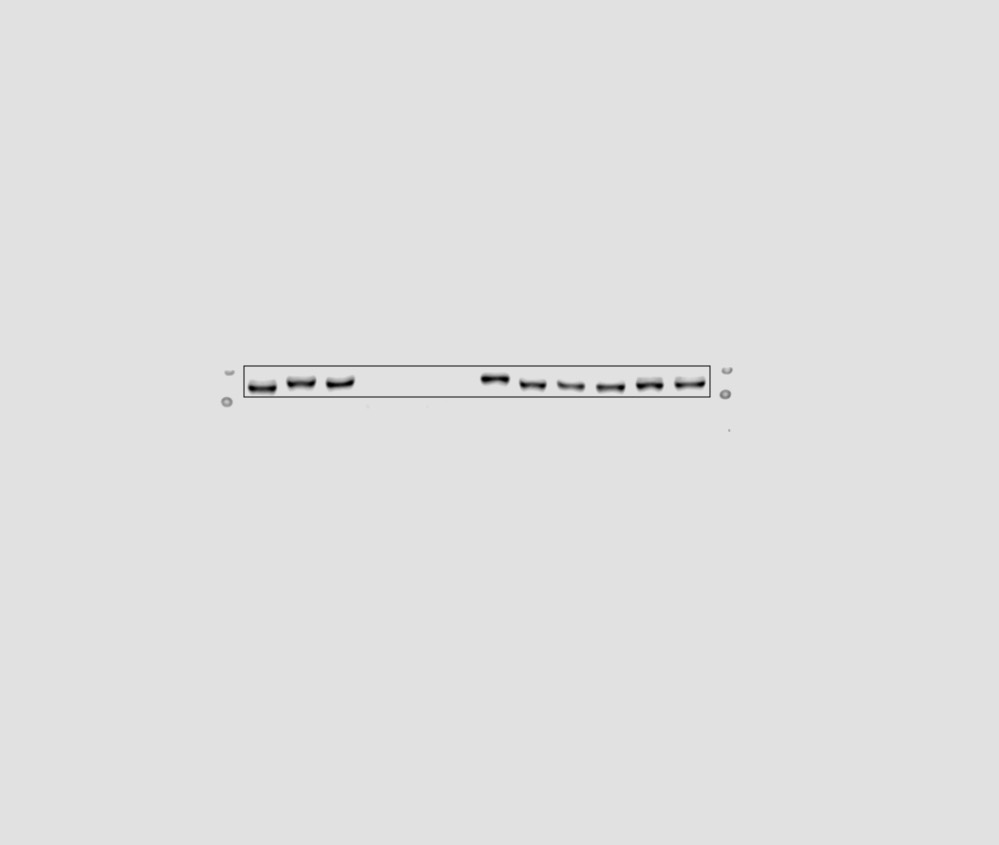

Supplement: Figure 5—source data 2. [file elife-97577-fig5-data2.zip › Figure5A_SourceData2/Fig5A_Box_SUZ12.jpeg]

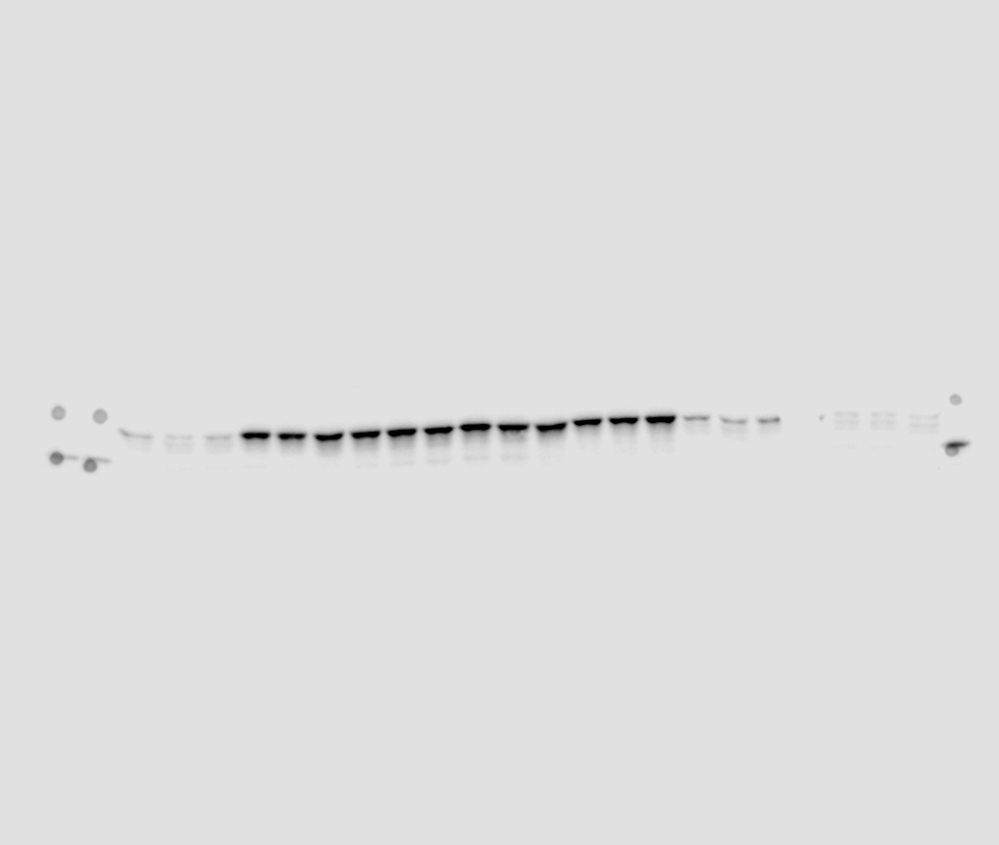

Supplement: Figure 6—source data 1. [file elife-97577-fig6-data1.zip › Figure6C_SourceData1/Fig6C_CCND1.jpeg]

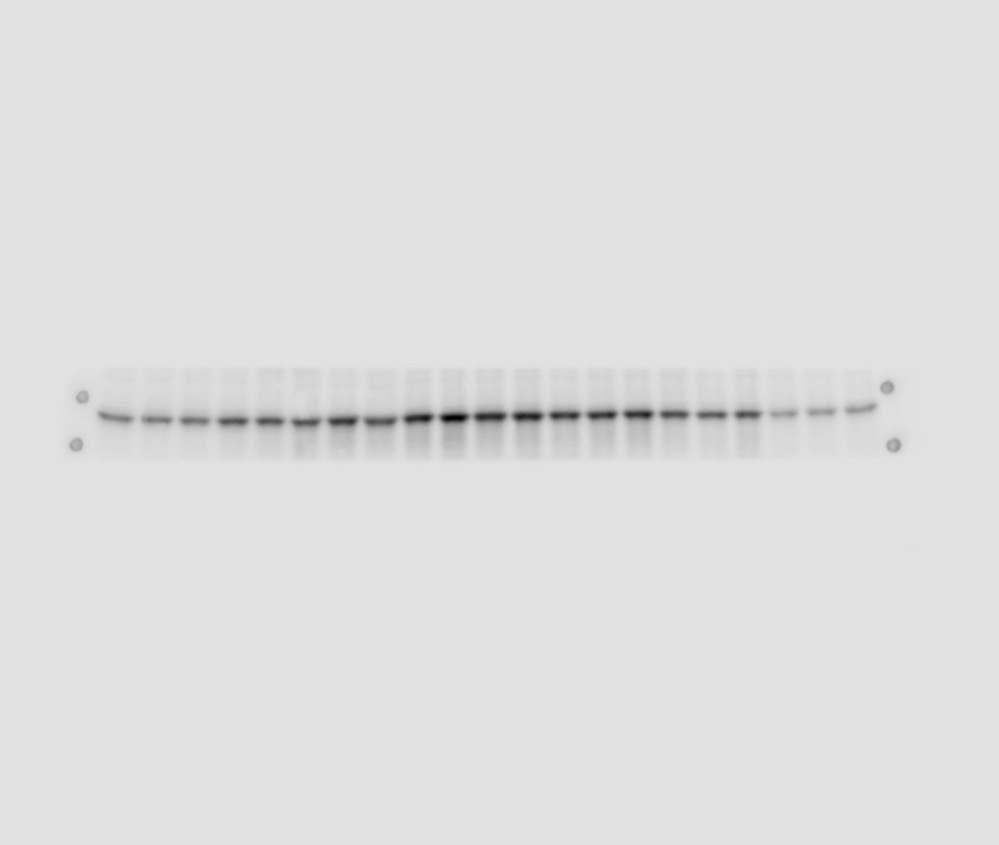

Supplement: Figure 6—source data 1. [file elife-97577-fig6-data1.zip › Figure6C_SourceData1/Fig6C_CCND2.jpeg]

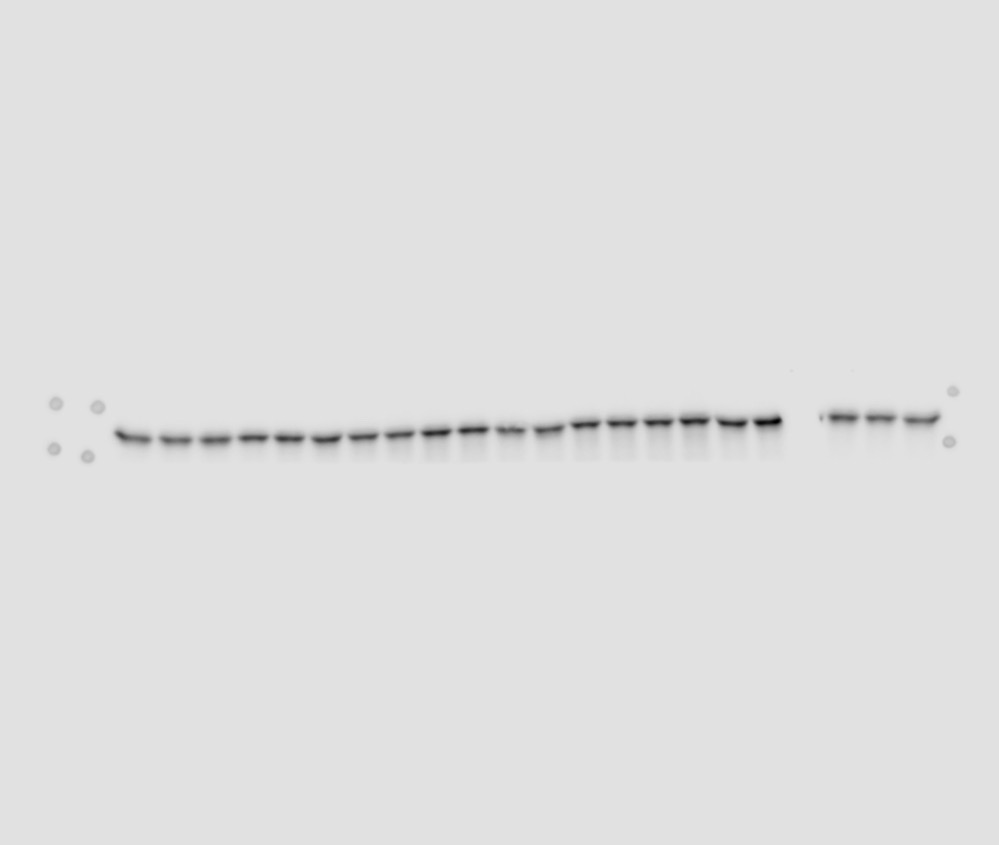

Supplement: Figure 6—source data 1. [file elife-97577-fig6-data1.zip › Figure6C_SourceData1/Fig6C_CCND3.jpeg]

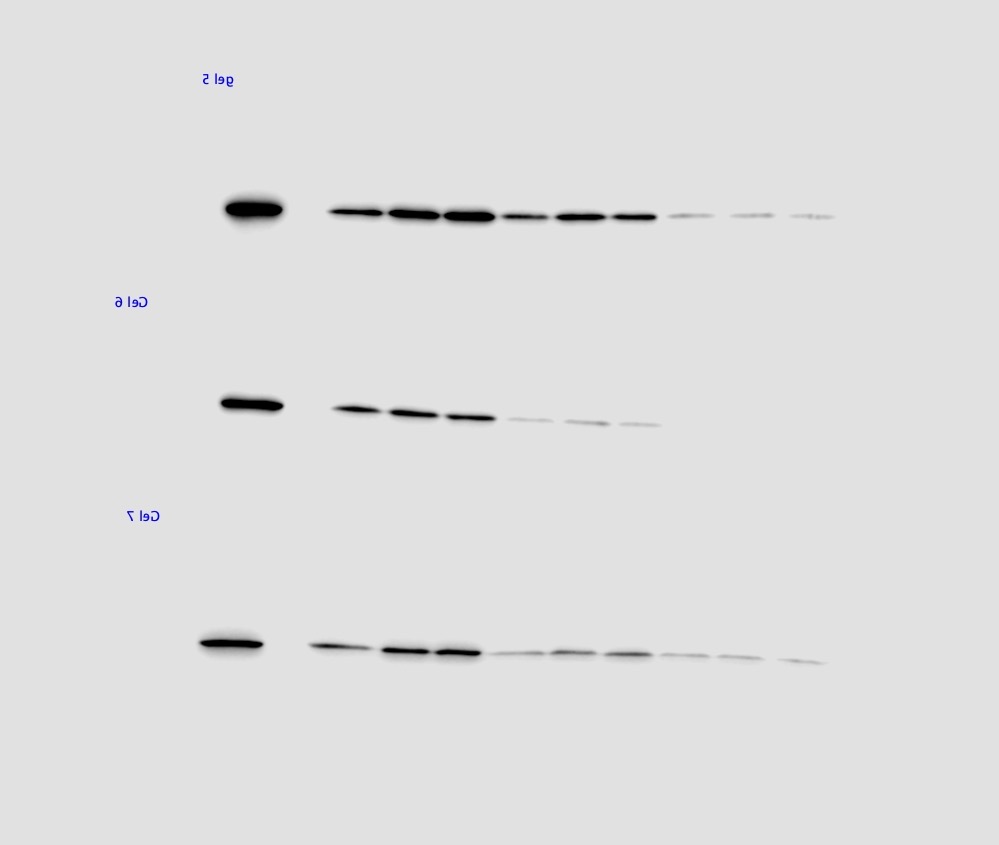

Supplement: Figure 6—source data 1. [file elife-97577-fig6-data1.zip › Figure6D_SourceData1/Fig6D_NR6_COS7_H3K27me3.jpeg]

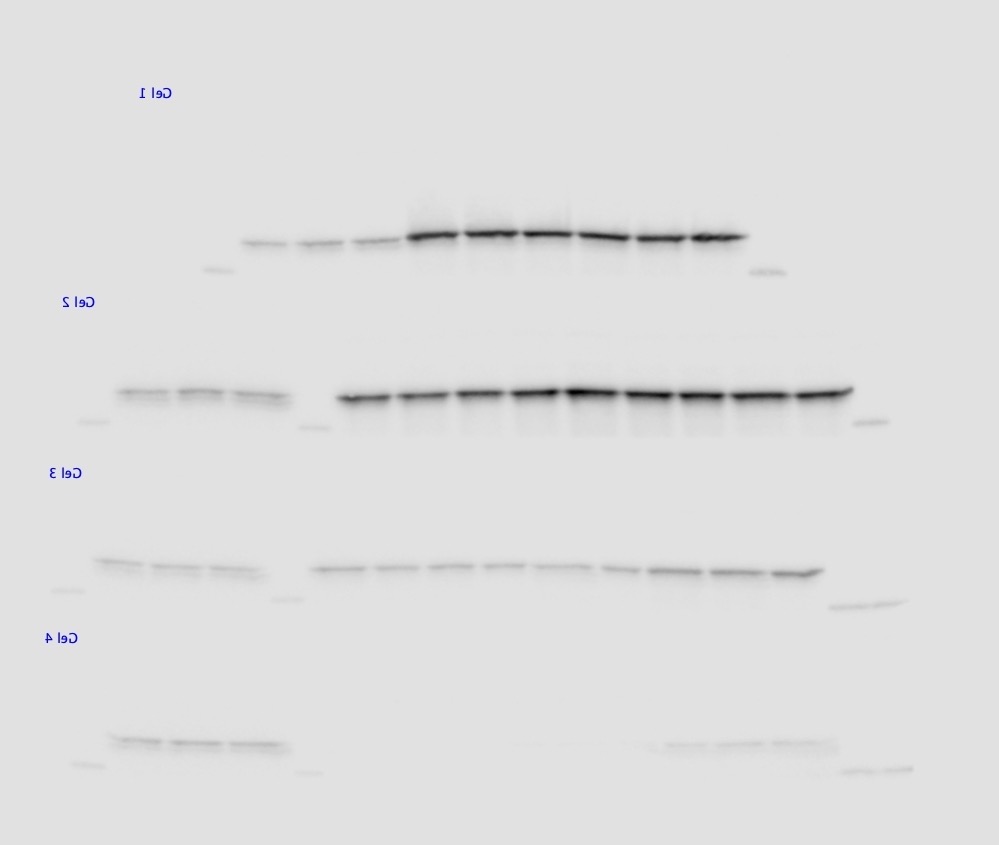

Supplement: Figure 6—source data 1. [file elife-97577-fig6-data1.zip › Figure6D_SourceData1/Fig6D_HAP1_CCND1.jpeg]

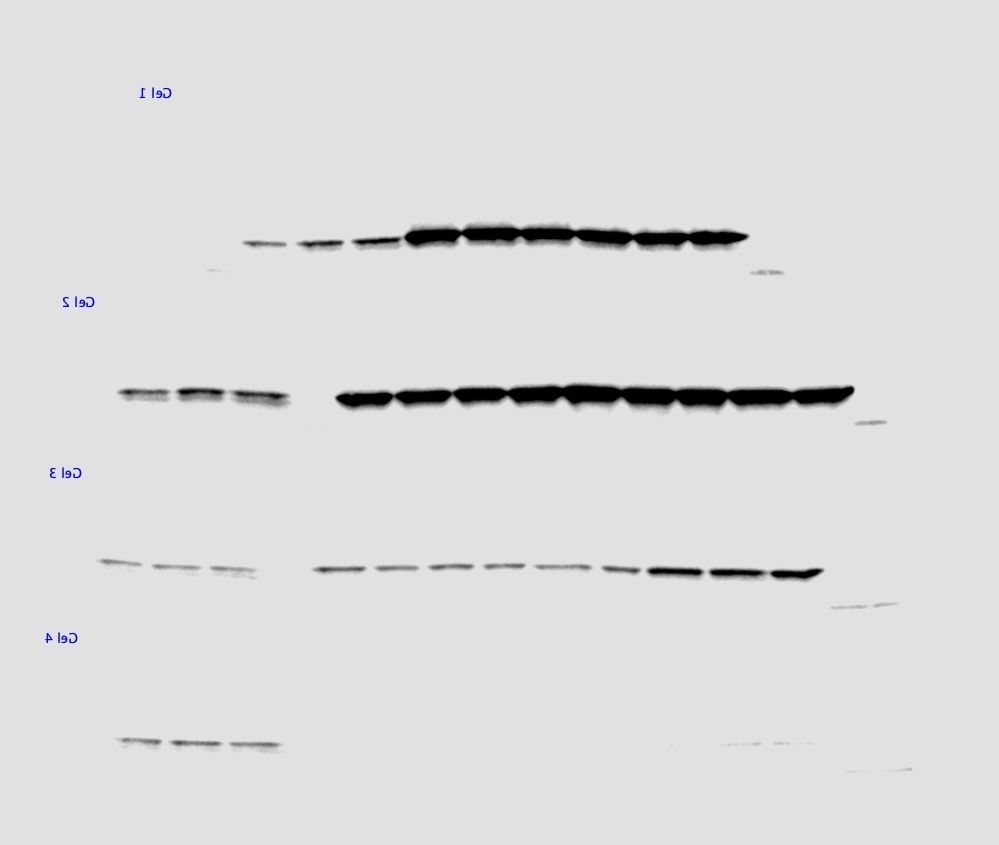

Supplement: Figure 6—source data 1. [file elife-97577-fig6-data1.zip › Figure6D_SourceData1/Fig6D_MDA-MB-231_CCND1.jpeg]

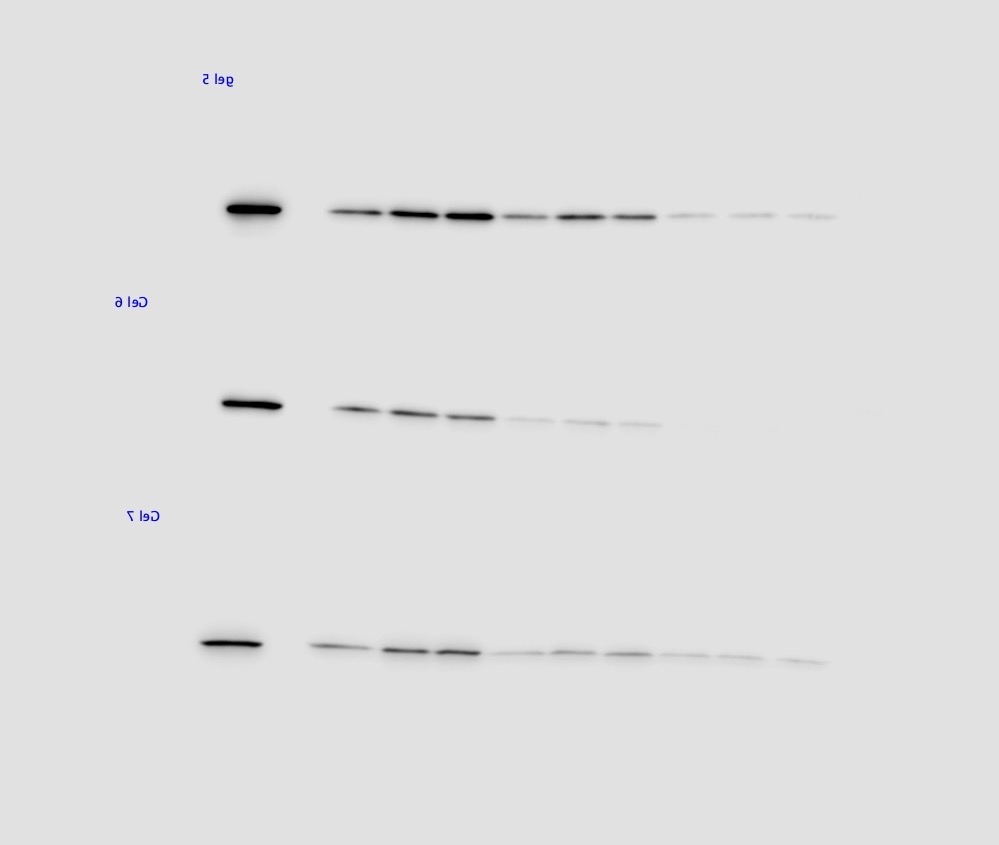

Supplement: Figure 6—source data 1. [file elife-97577-fig6-data1.zip › Figure6D_SourceData1/Fig6D_LNCaP-M1-2166_H3K27me3.jpeg]

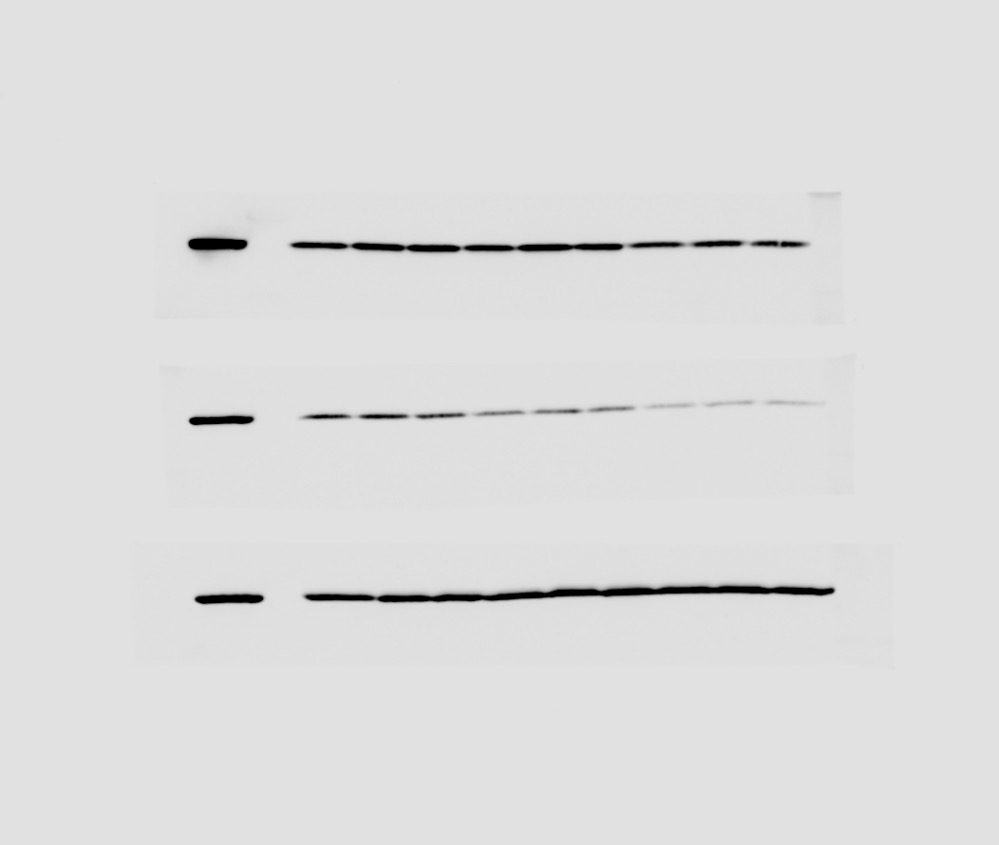

Supplement: Figure 6—source data 1. [file elife-97577-fig6-data1.zip › Figure6D_SourceData1/Fig6D_COS7_H3.jpeg]

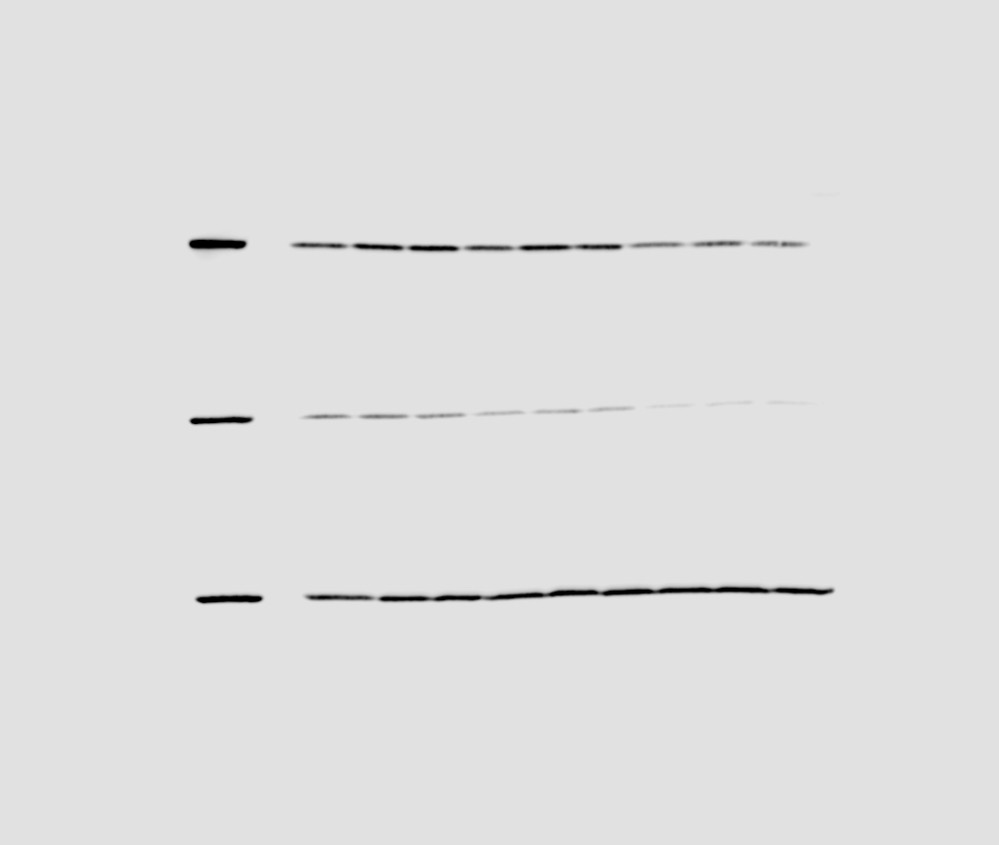

Supplement: Figure 6—source data 1. [file elife-97577-fig6-data1.zip › Figure6D_SourceData1/Fig6D_LNCaP_NR6_H3.jpeg]

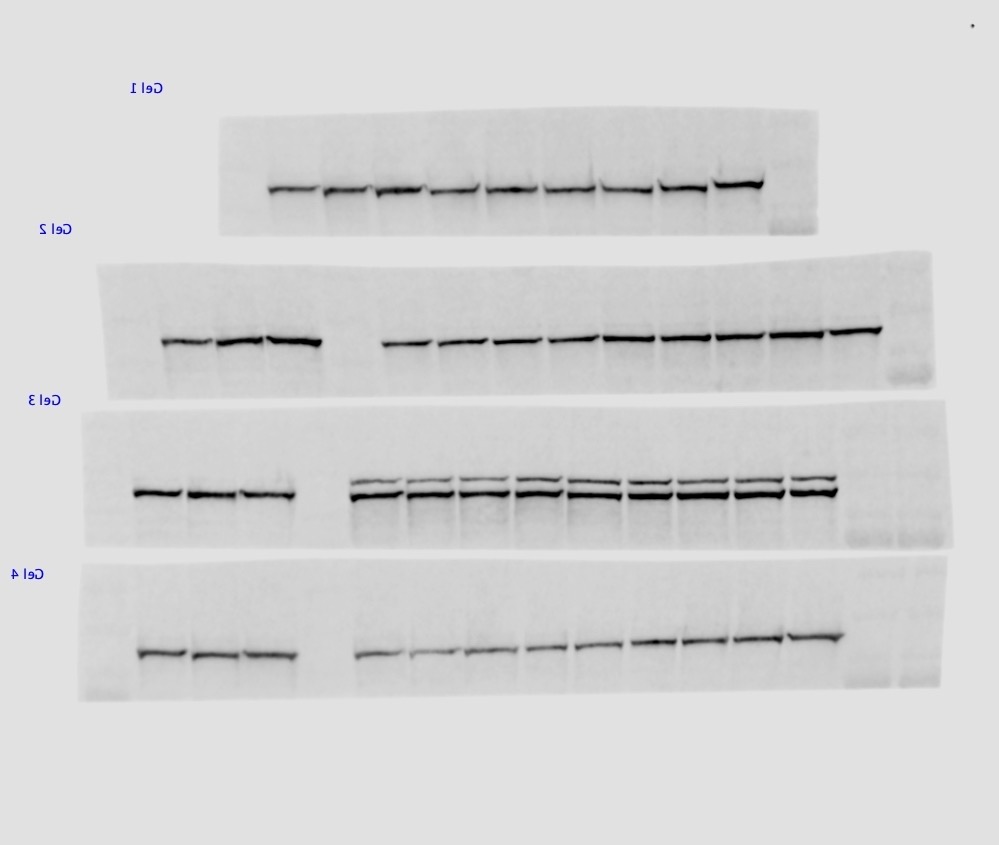

Supplement: Figure 6—source data 1. [file elife-97577-fig6-data1.zip › Figure6D_SourceData1/Fig6D_HAP1_MDA-MB-231_KBM7_Vinc.jpeg]

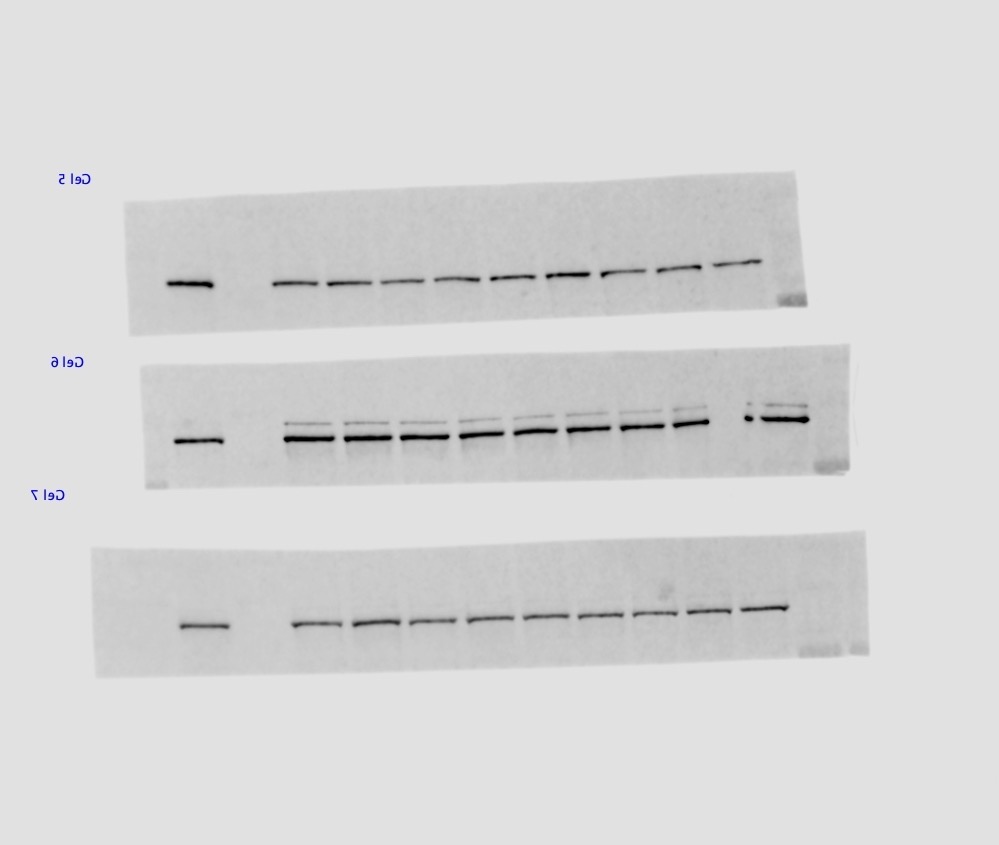

Supplement: Figure 6—source data 1. [file elife-97577-fig6-data1.zip › Figure6D_SourceData1/Fig6D_LNCaP-M1-2166_NR6_COS7_Vinc.jpeg]

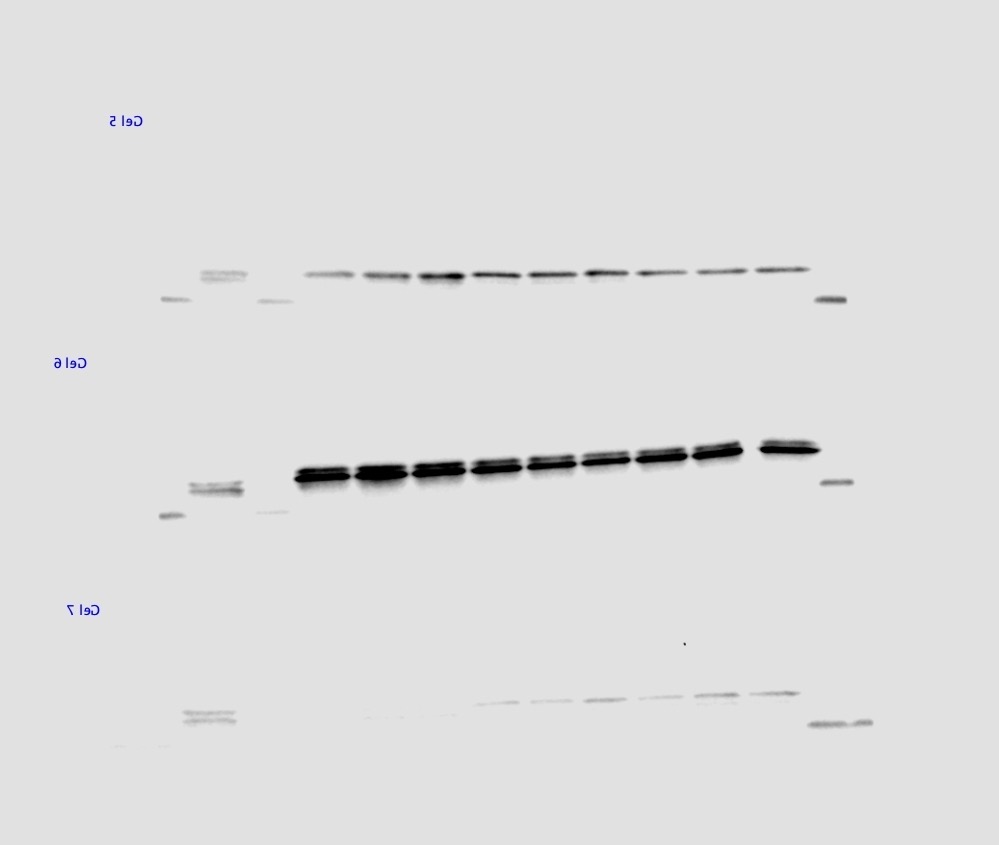

Supplement: Figure 6—source data 1. [file elife-97577-fig6-data1.zip › Figure6D_SourceData1/Fig6D_LNCaP-M1-2166_NR6_CCND1.jpeg]

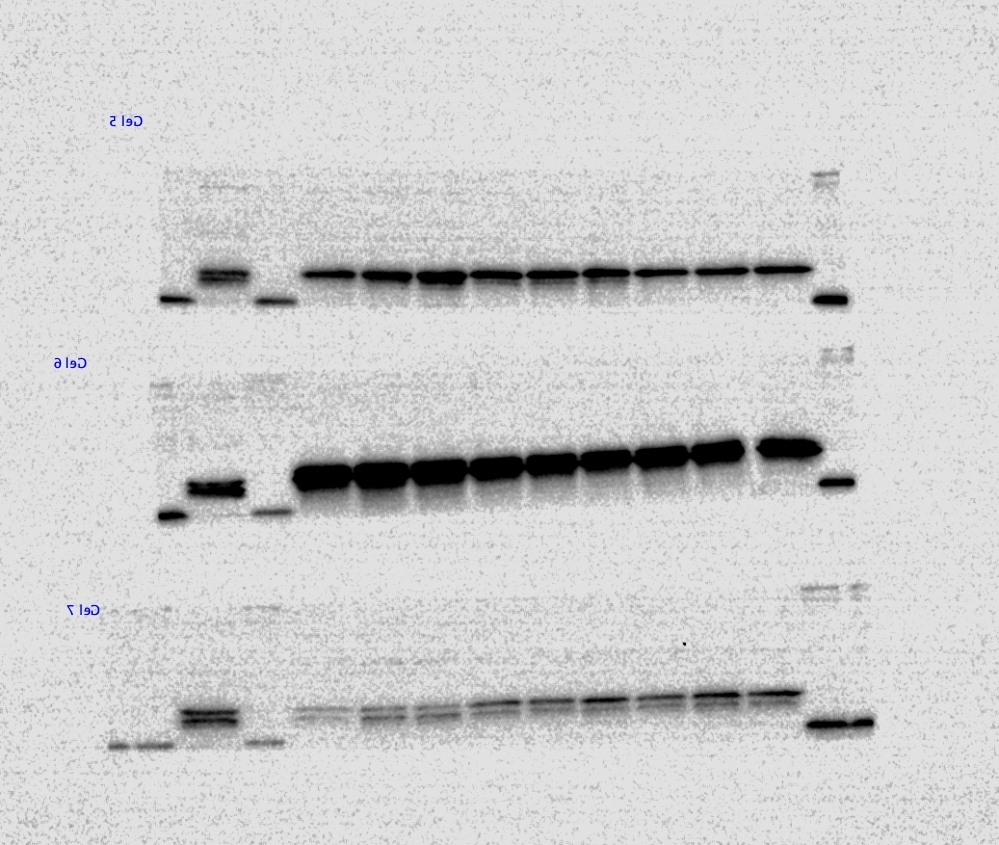

Supplement: Figure 6—source data 1. [file elife-97577-fig6-data1.zip › Figure6D_SourceData1/Fig6D_COS7_CCND1.jpeg]

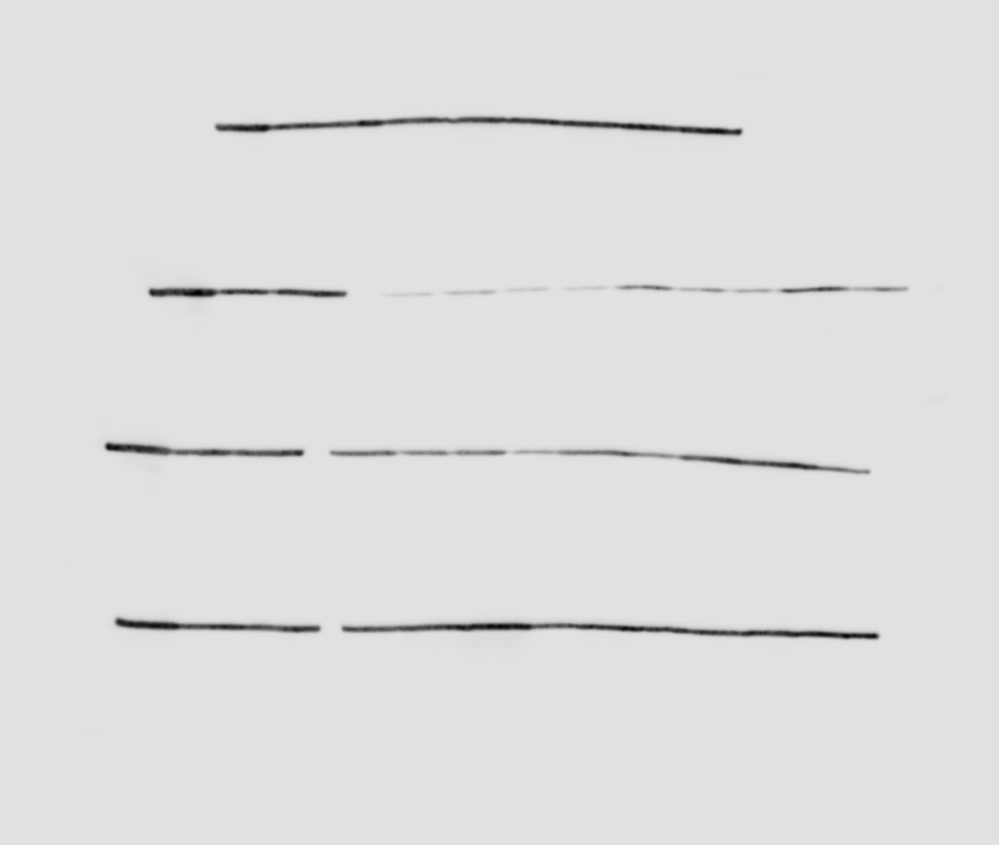

Supplement: Figure 6—source data 1. [file elife-97577-fig6-data1.zip › Figure6D_SourceData1/Fig6D_HAP1_MDA-MB-231_KBM7_H3.jpeg]

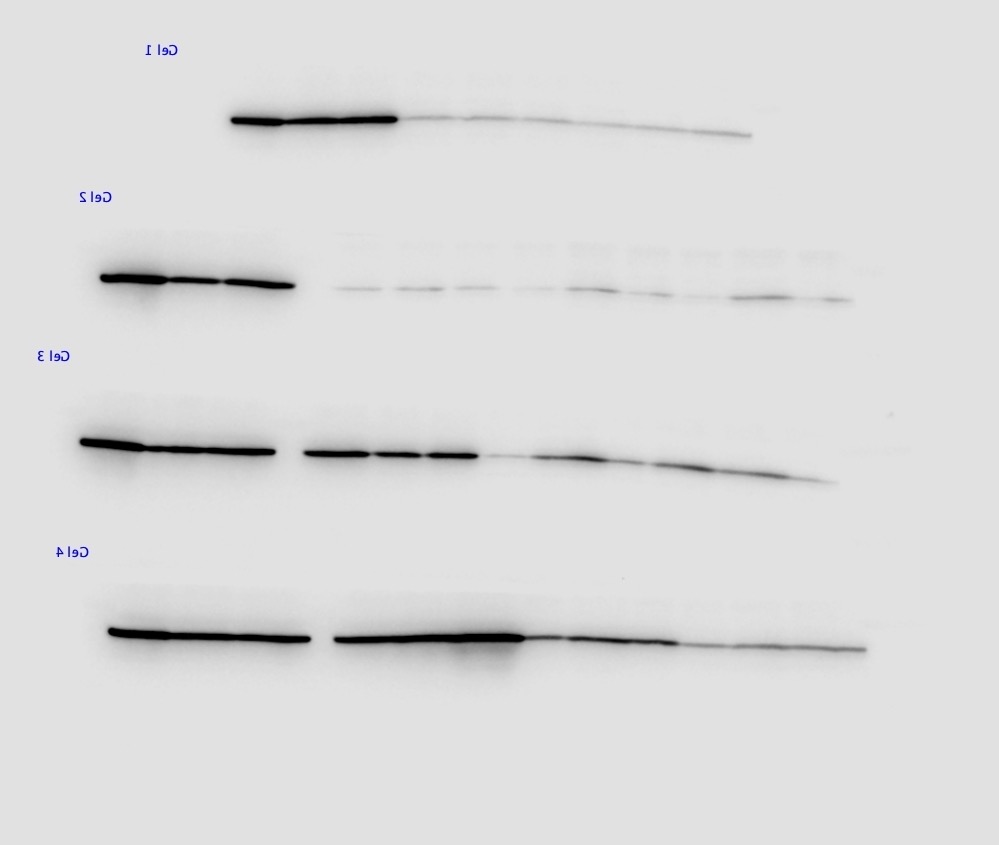

Supplement: Figure 6—source data 1. [file elife-97577-fig6-data1.zip › Figure6D_SourceData1/Fig6D_HAP1_MDA-MB-231_KBM7_H3K27me3.jpeg]

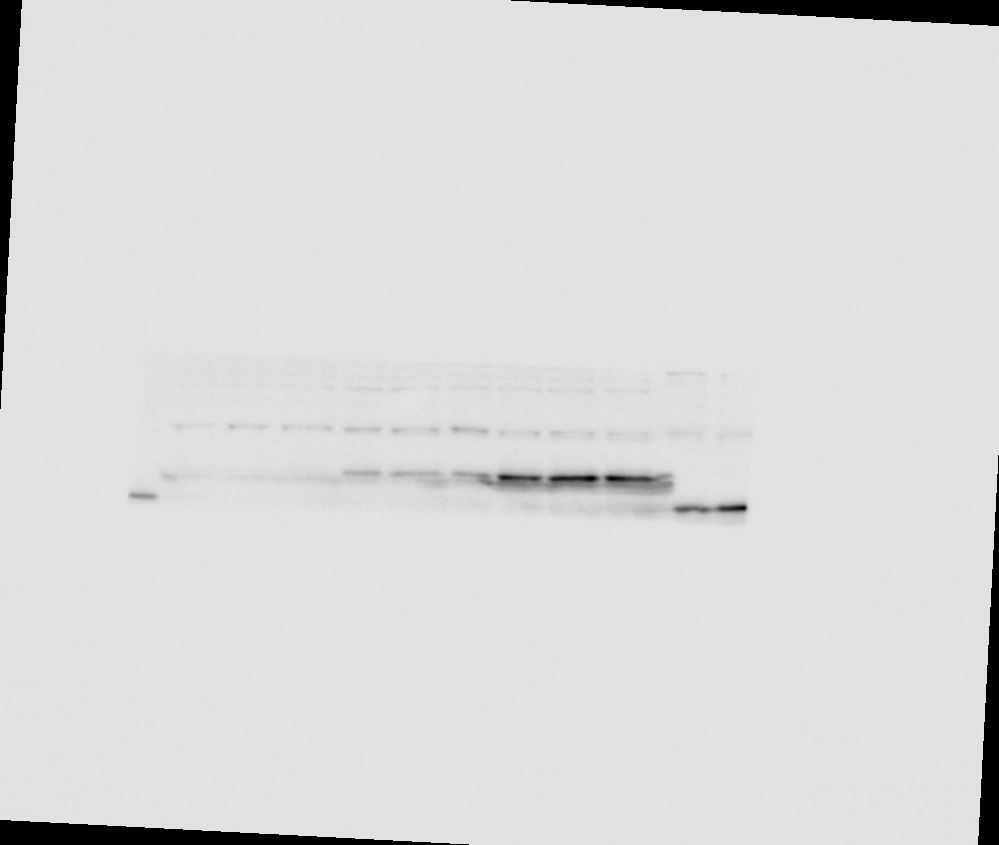

Supplement: Figure 6—source data 1. [file elife-97577-fig6-data1.zip › Figure6D_SourceData1/Fig6D_KBM7_CCND1.jpeg]

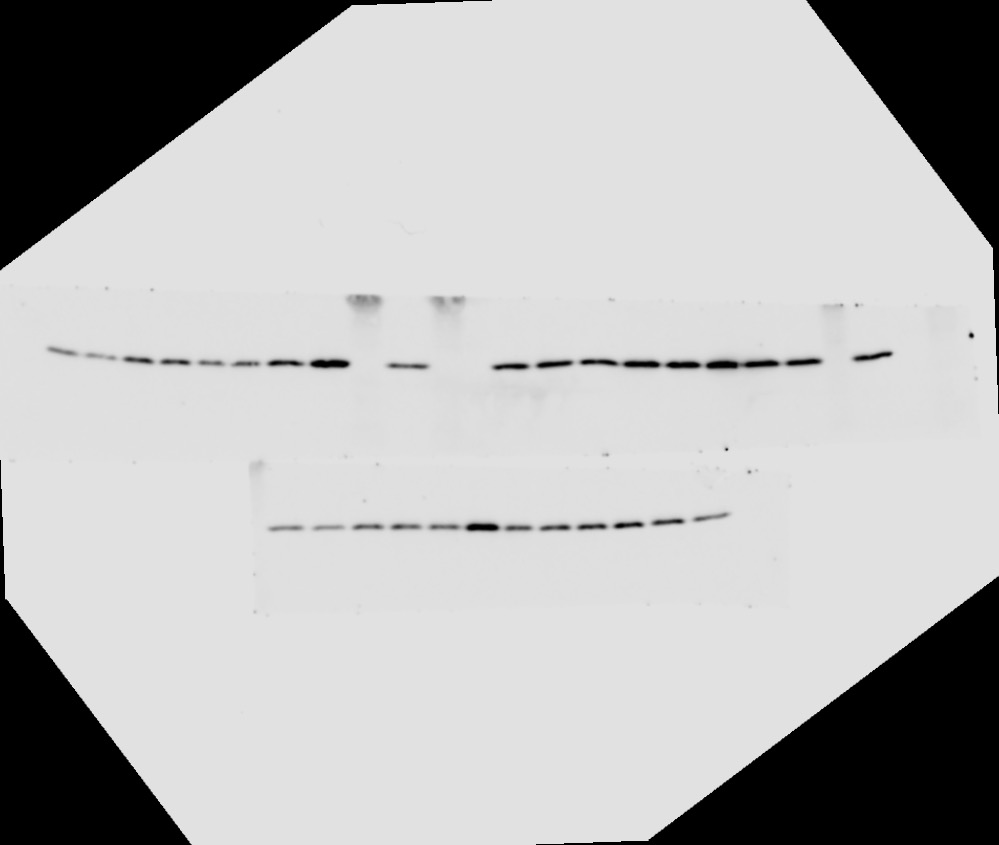

Supplement: Figure 6—source data 1. [file elife-97577-fig6-data1.zip › Figure6E_SourceData1/Fig6E_COS7_H3.jpeg]

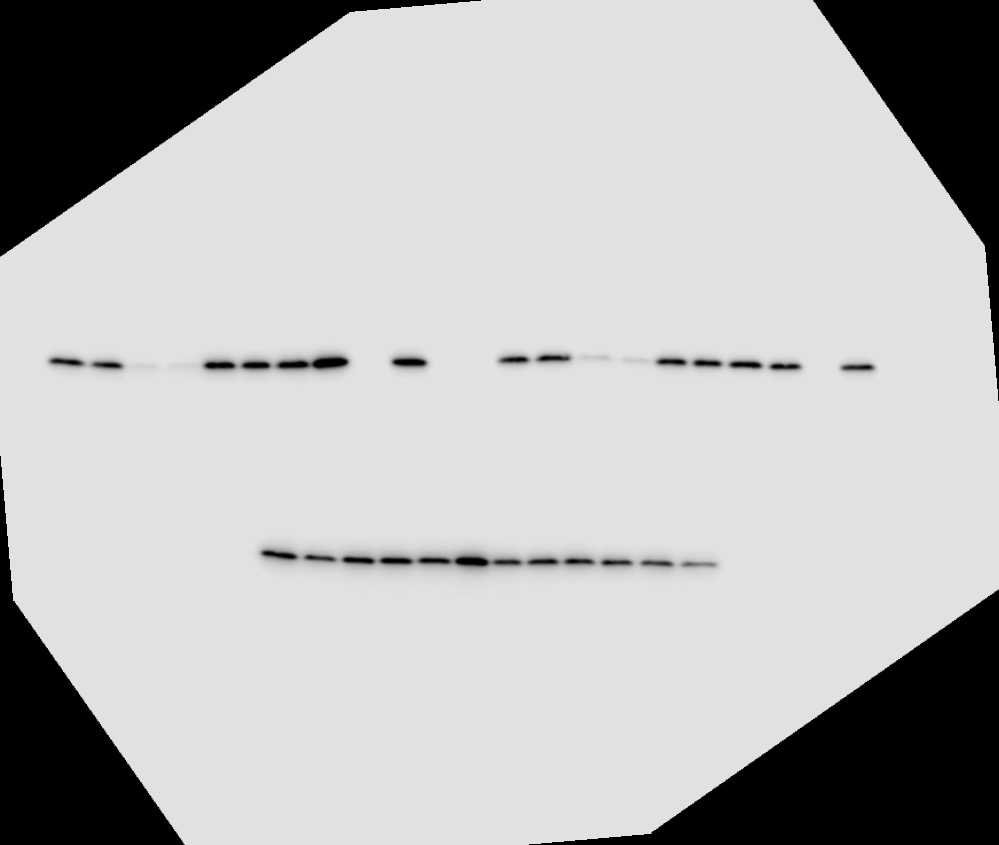

Supplement: Figure 6—source data 1. [file elife-97577-fig6-data1.zip › Figure6E_SourceData1/Fig6E_COS7_MDA-MB-231_H3K27me3.jpeg]

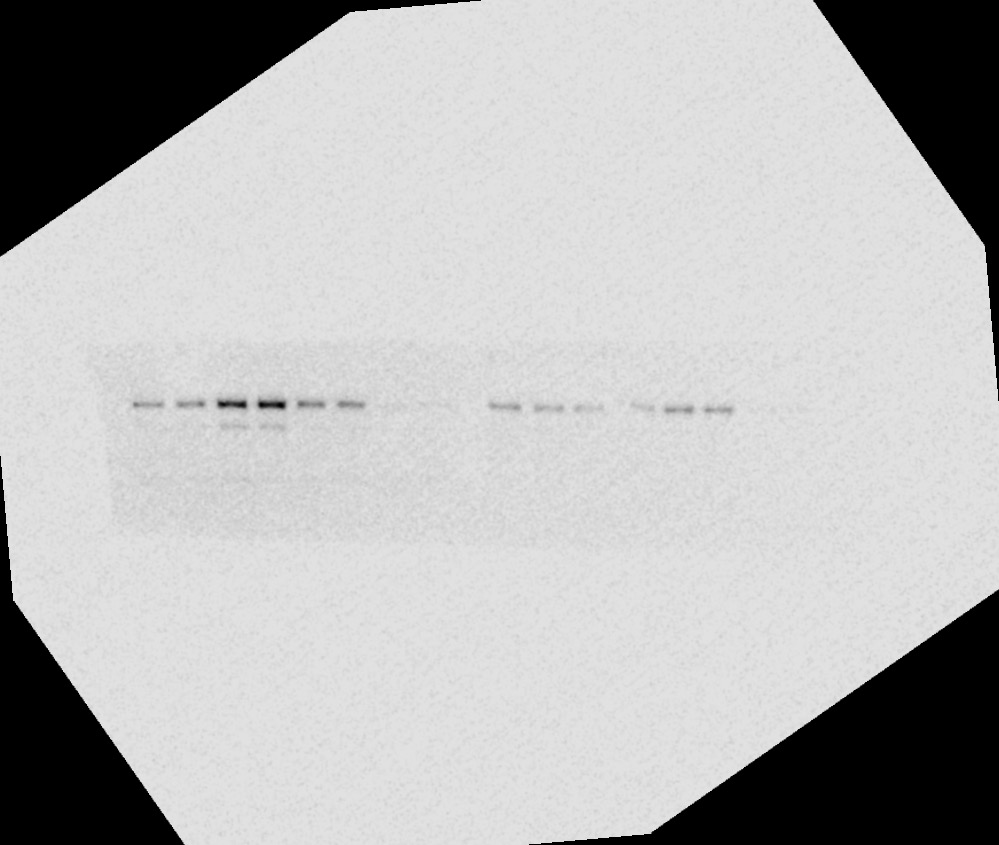

Supplement: Figure 6—source data 1. [file elife-97577-fig6-data1.zip › Figure6E_SourceData1/Fig6E_MDA-MB-231_JARID2.jpeg]

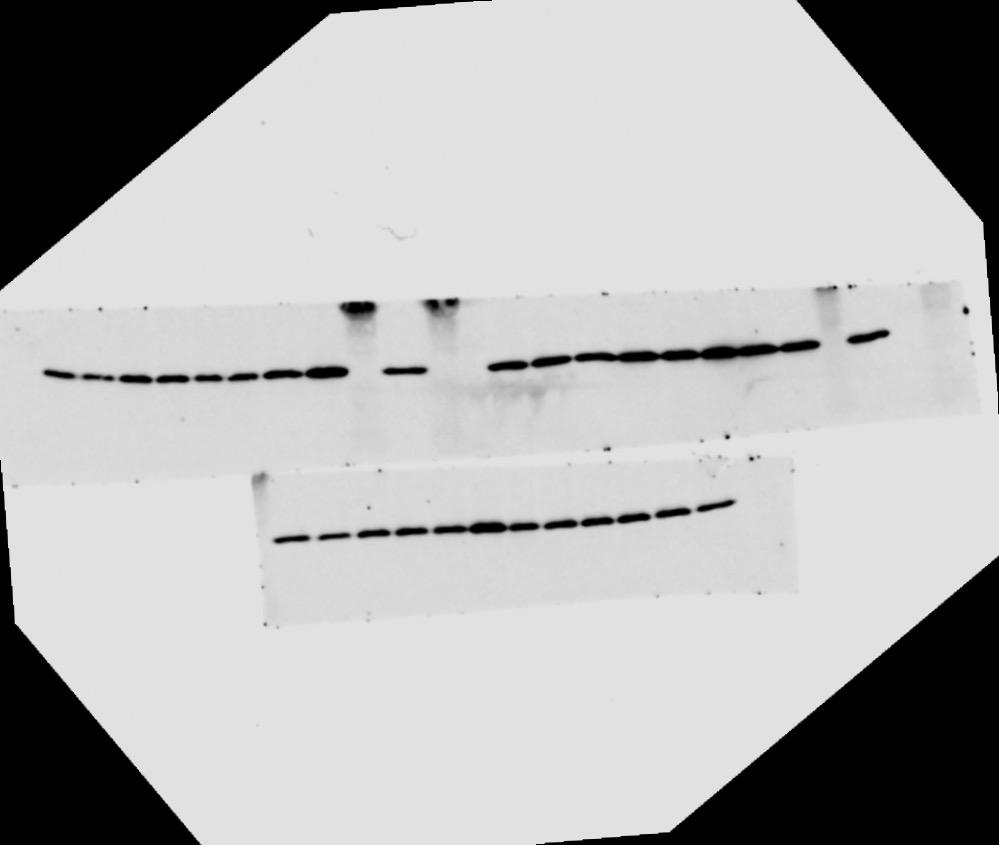

Supplement: Figure 6—source data 1. [file elife-97577-fig6-data1.zip › Figure6E_SourceData1/Fig6E_MDA-MB-231_H3.jpeg]

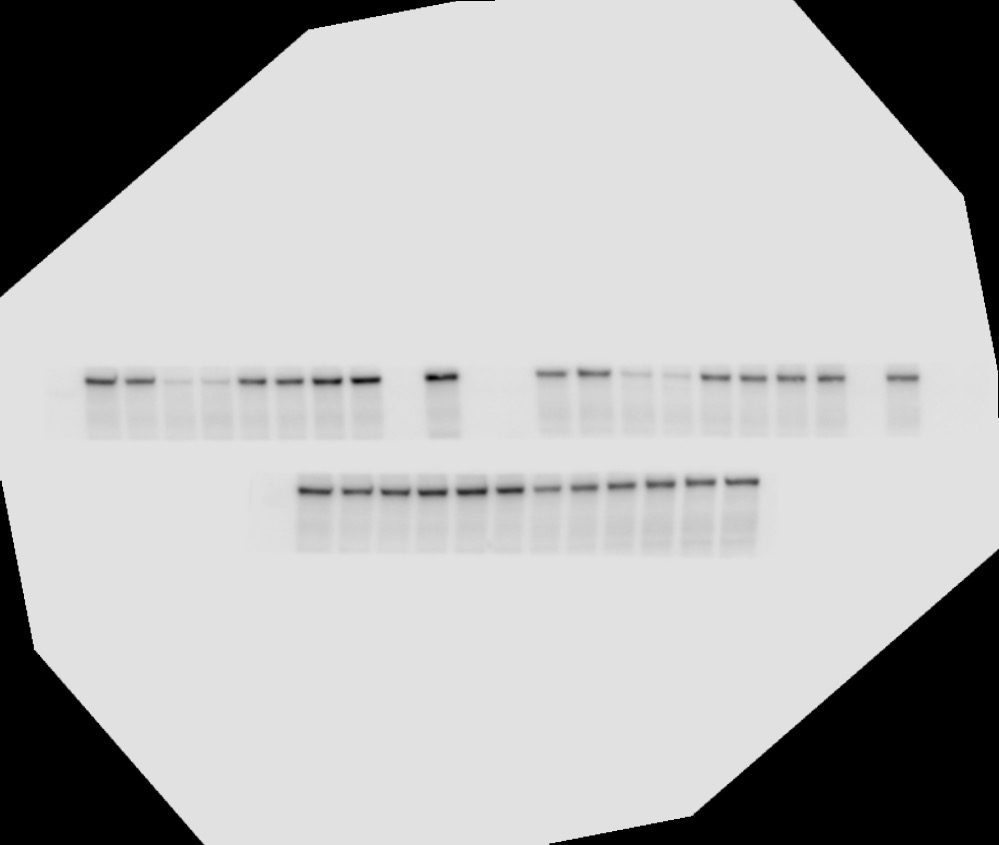

Supplement: Figure 6—source data 1. [file elife-97577-fig6-data1.zip › Figure6E_SourceData1/Fig6E_MDA-MB-231_COS7_SUZ12.jpeg]

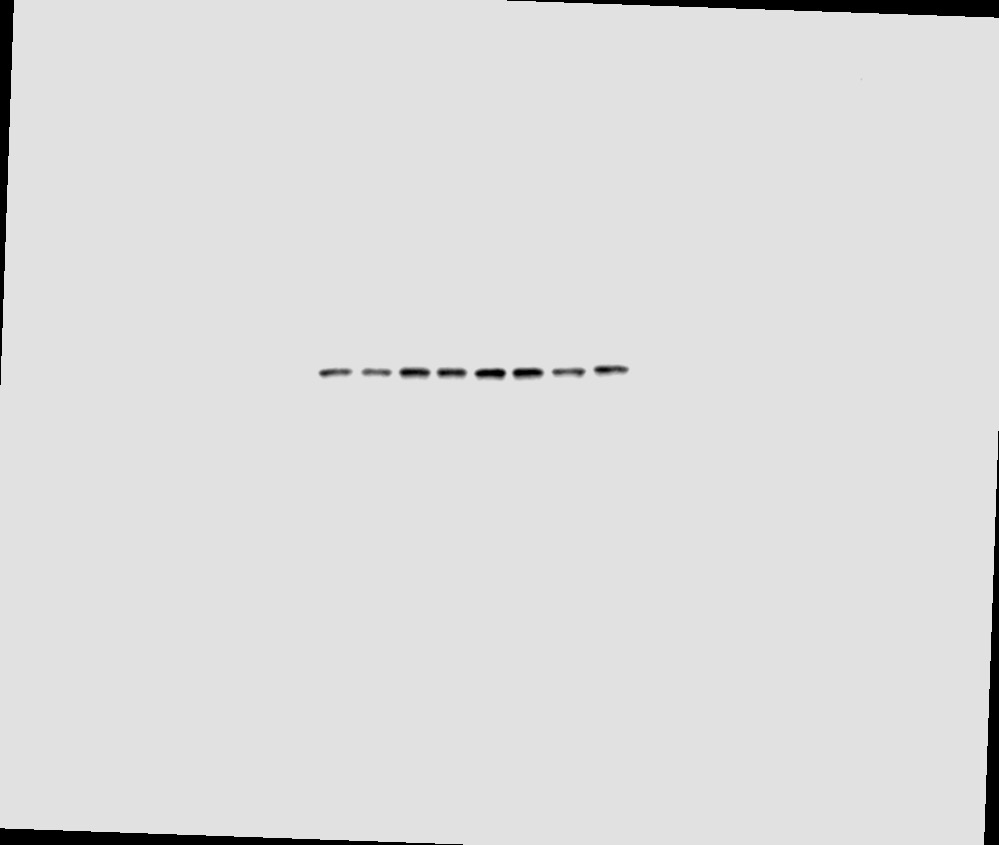

Supplement: Figure 6—source data 1. [file elife-97577-fig6-data1.zip › Figure6E_SourceData1/Fig6E_MDA-MB-231_CCND1.jpeg]

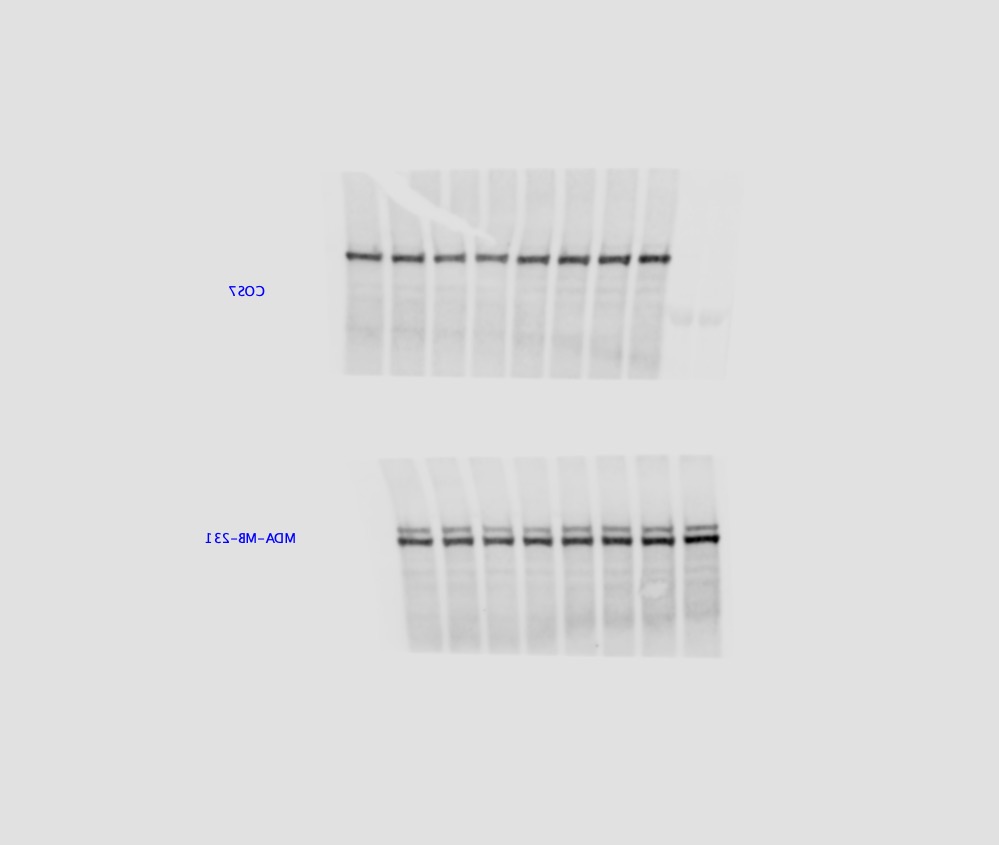

Supplement: Figure 6—source data 1. [file elife-97577-fig6-data1.zip › Figure6E_SourceData1/Fig6E_COS7_MDA-MB-231_Vinc.jpeg]

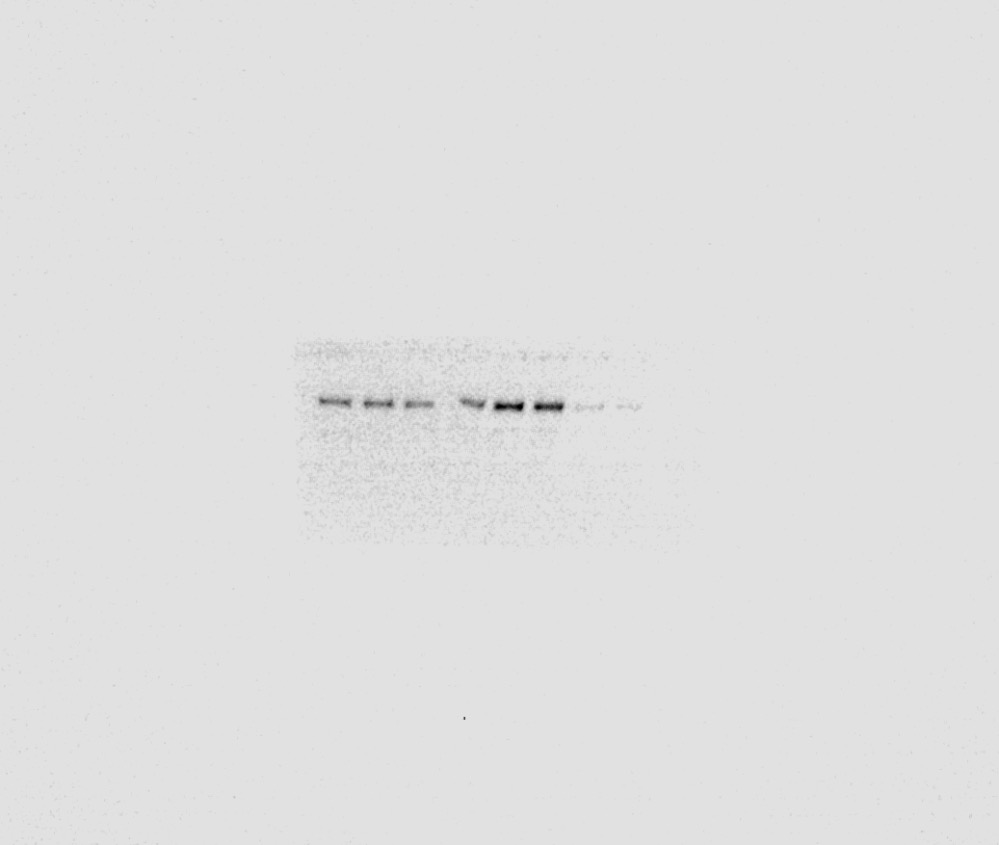

Supplement: Figure 6—source data 1. [file elife-97577-fig6-data1.zip › Figure6E_SourceData1/Fig6E_COS7_JARID2.jpeg]

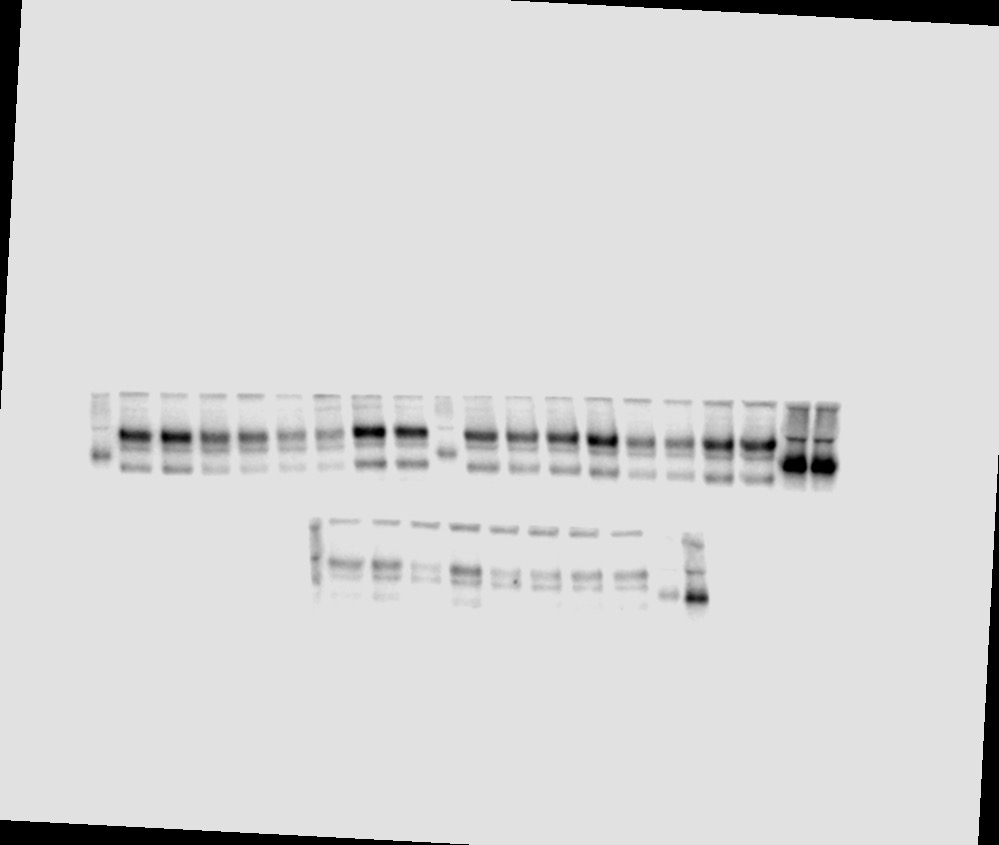

Supplement: Figure 6—source data 1. [file elife-97577-fig6-data1.zip › Figure6E_SourceData1/Fig6E_MDA-MB-231_COS7_MTF2.jpeg]

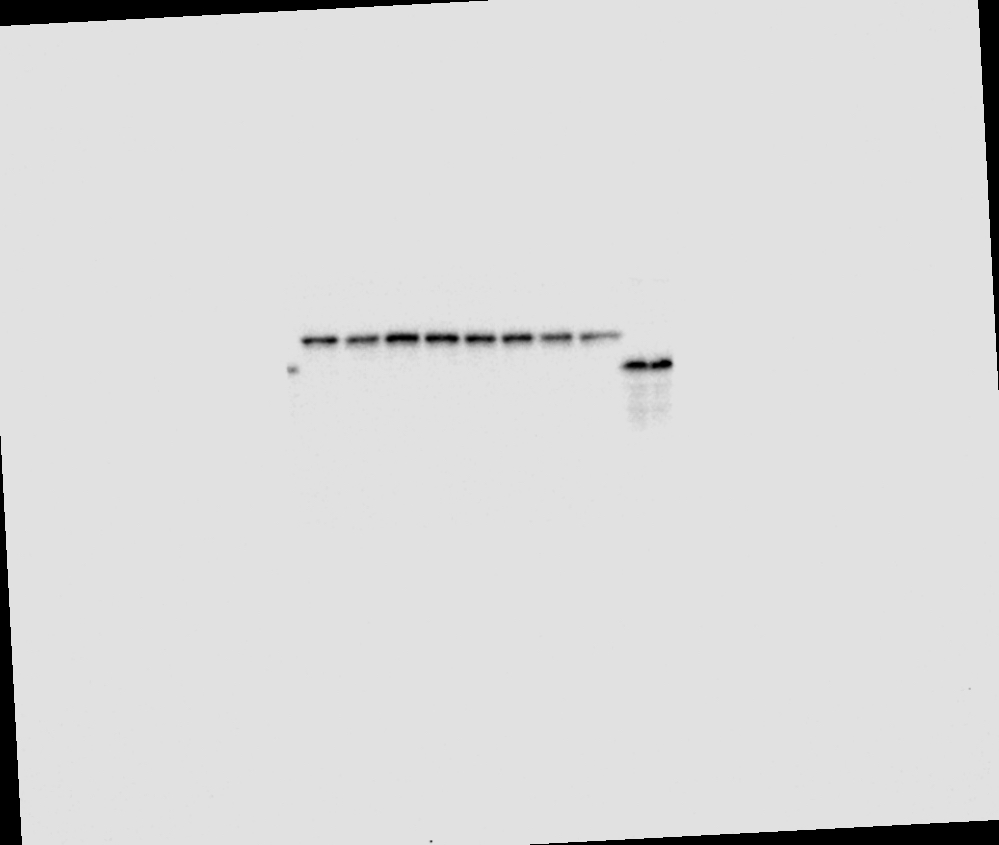

Supplement: Figure 6—source data 1. [file elife-97577-fig6-data1.zip › Figure6E_SourceData1/Fig6E_COS7_CCND1.jpeg]

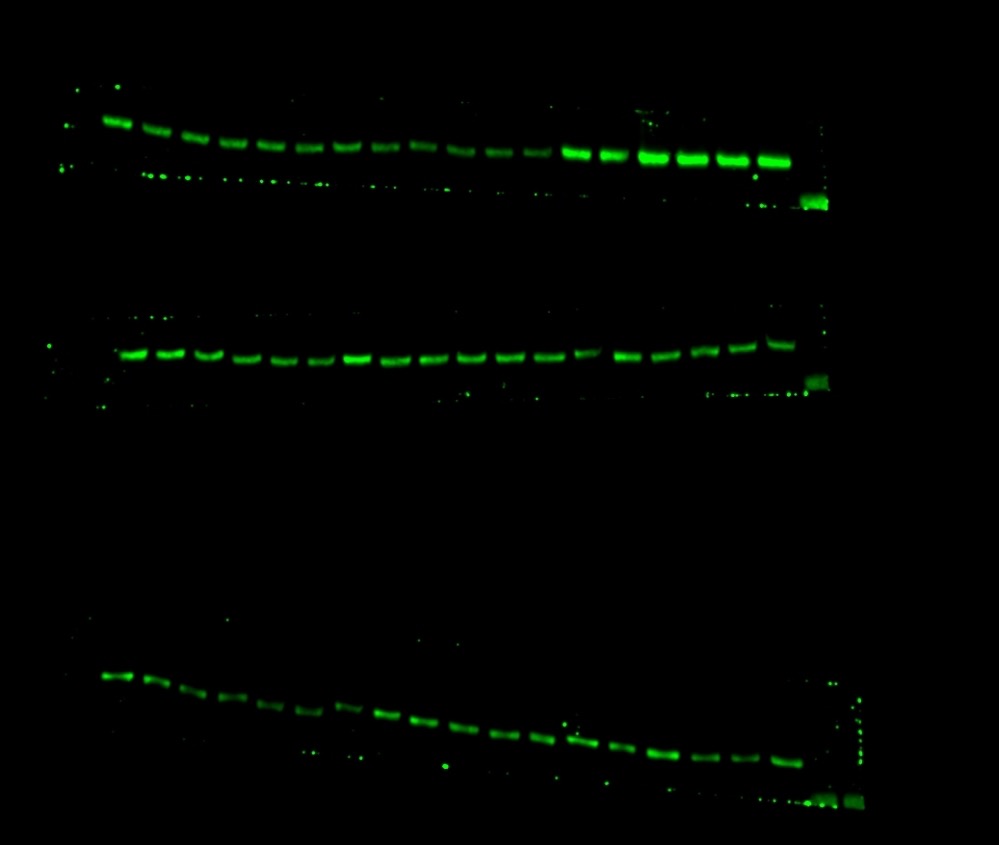

Supplement: Figure 6—source data 1. [file elife-97577-fig6-data1.zip › Figure6F_SourceData1/Fig6F_RB1.jpeg]

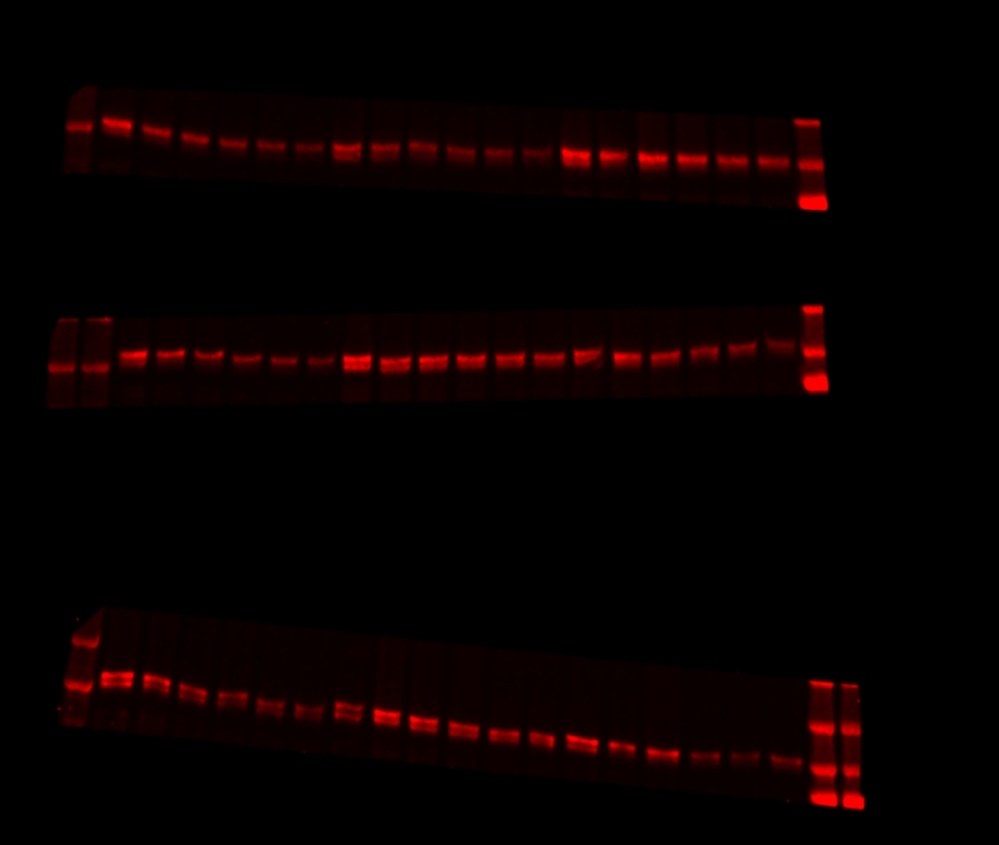

Supplement: Figure 6—source data 1. [file elife-97577-fig6-data1.zip › Figure6F_SourceData1/811_RB1.jpeg]

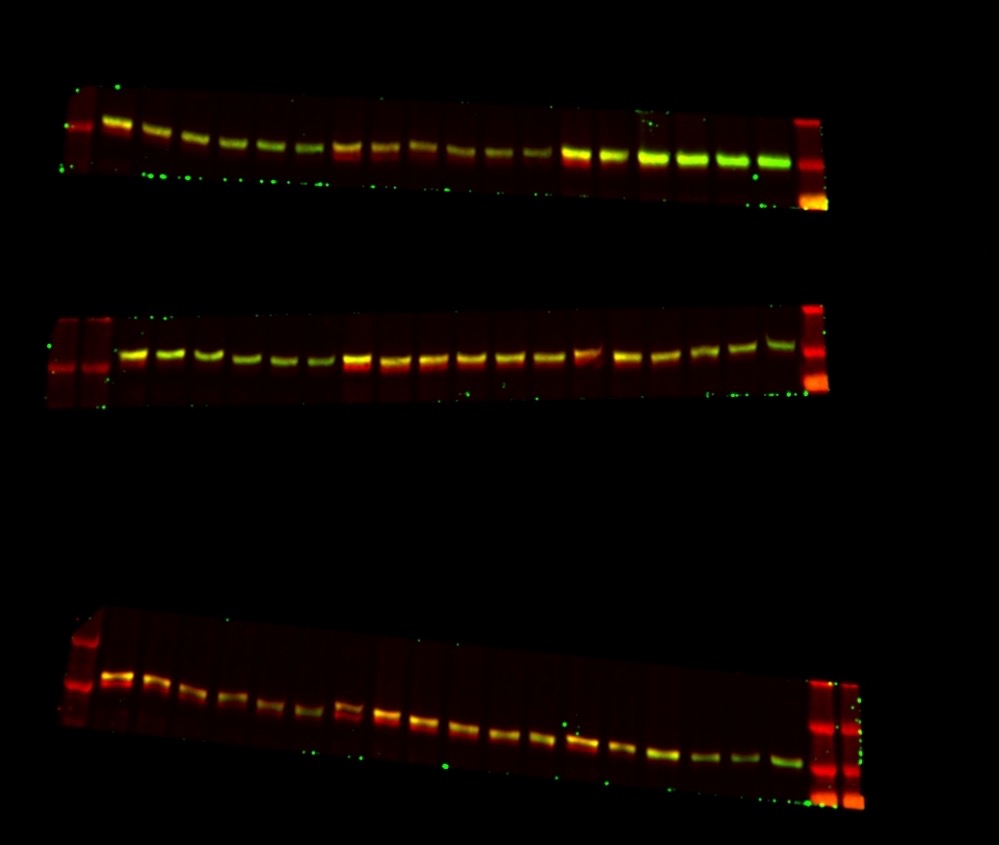

Supplement: Figure 6—source data 1. [file elife-97577-fig6-data1.zip › Figure6F_SourceData1/Fig6F_P-RB1_RB1_Composite.jpeg]

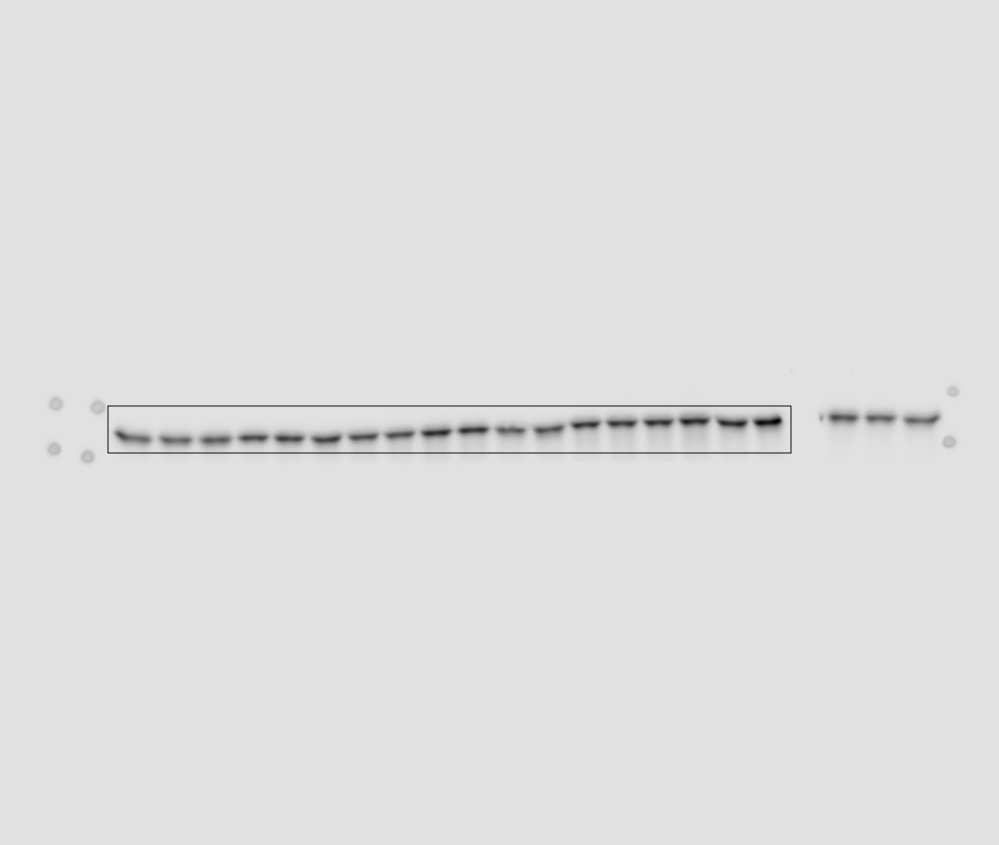

Supplement: Figure 6—source data 2. [file elife-97577-fig6-data2.zip › Figure6C_SourceData2/Fig6C_Box_CCND3.jpeg]

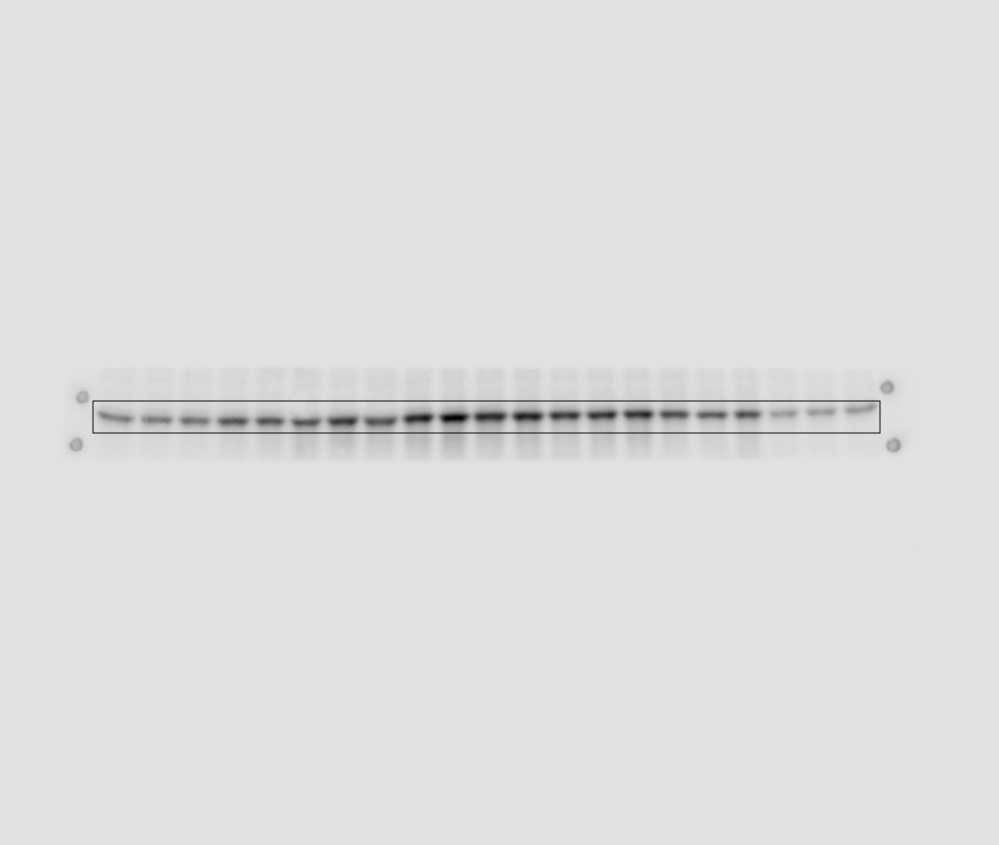

Supplement: Figure 6—source data 2. [file elife-97577-fig6-data2.zip › Figure6C_SourceData2/Fig6C_Box_CCND2.jpeg]

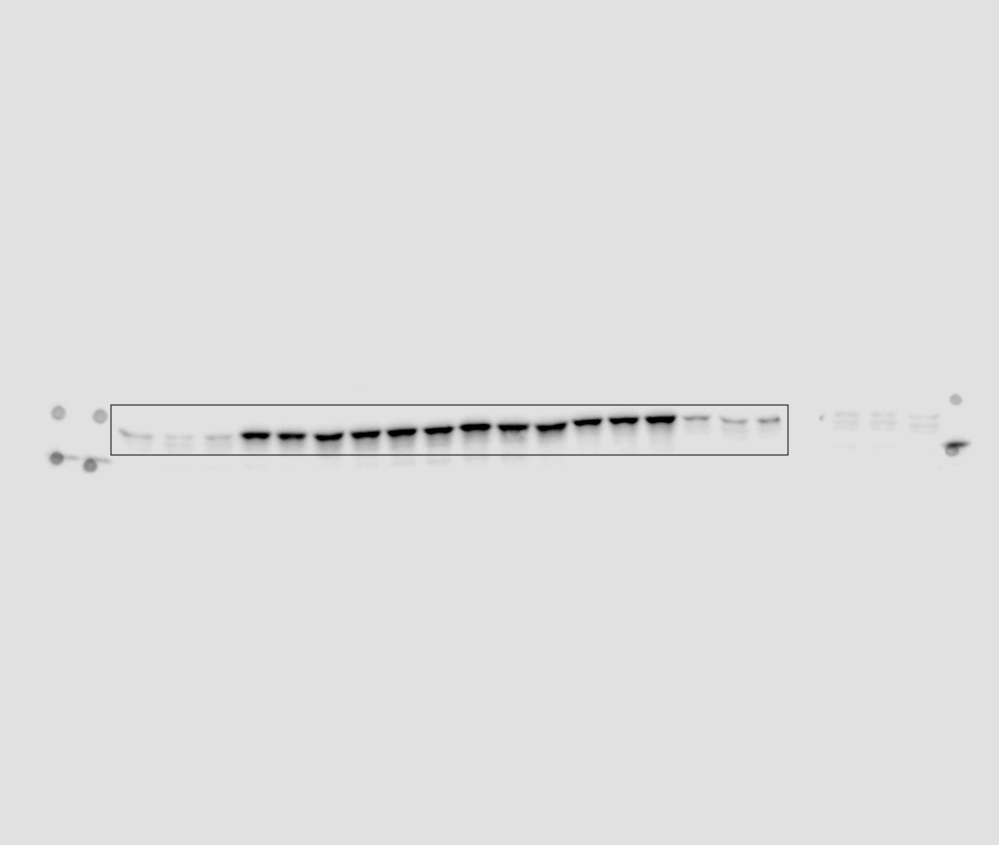

Supplement: Figure 6—source data 2. [file elife-97577-fig6-data2.zip › Figure6C_SourceData2/Fig6C_Box_CCND1.jpg]

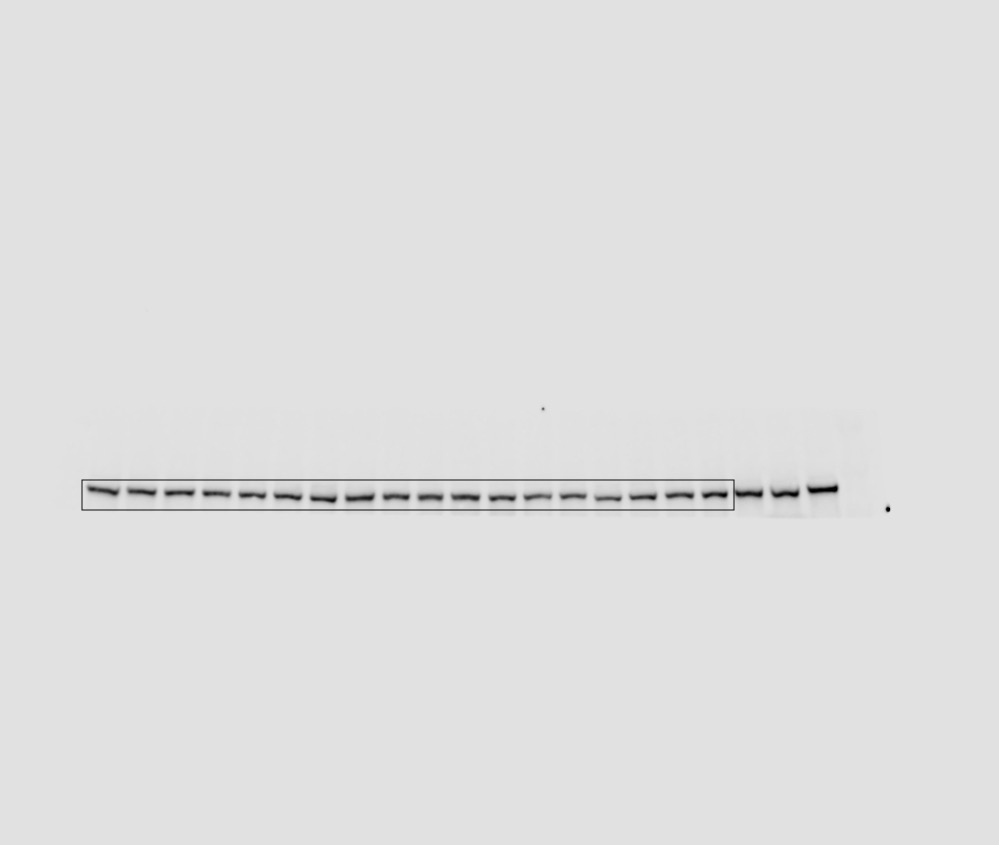

Supplement: Figure 6—source data 2. [file elife-97577-fig6-data2.zip › Figure6C_SourceData2/Fig6C_Box_Vinc.jpeg]

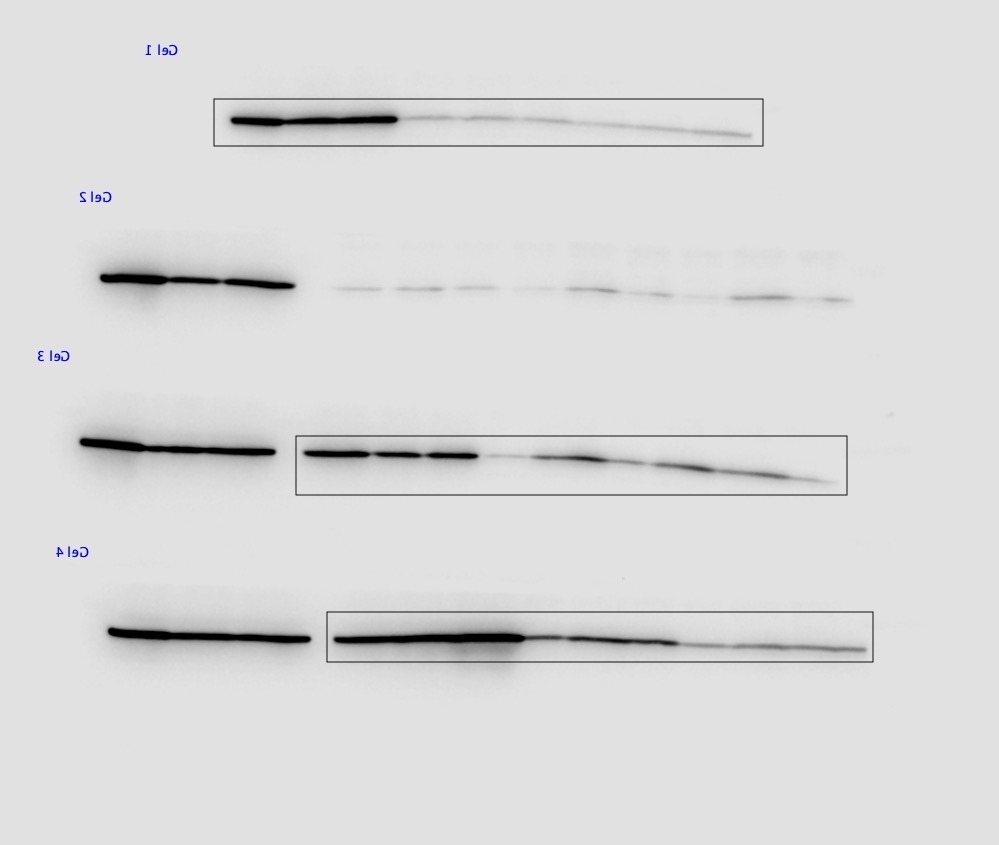

Supplement: Figure 6—source data 2. [file elife-97577-fig6-data2.zip › Figure6D_SourceData2/Fig6D_Box_HAP1_MDA-MB-231_KBM7_H3K27me3.jpeg]

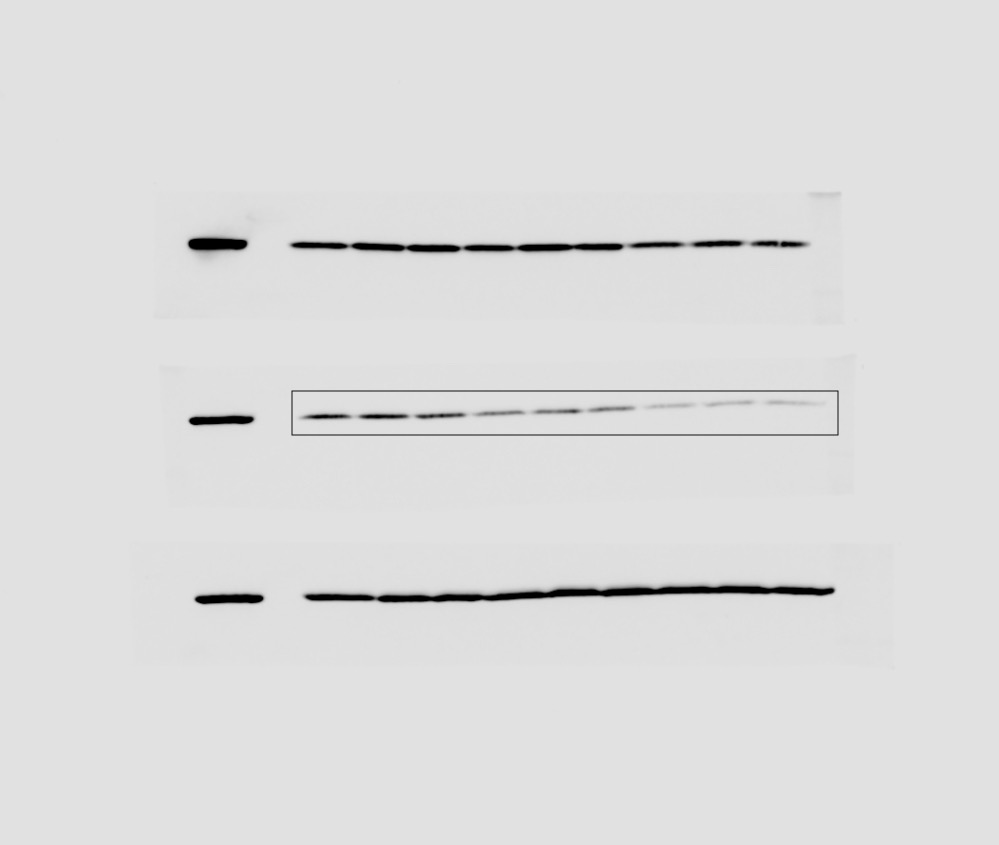

Supplement: Figure 6—source data 2. [file elife-97577-fig6-data2.zip › Figure6D_SourceData2/Fig6D_Box_NR6_H3.jpeg]

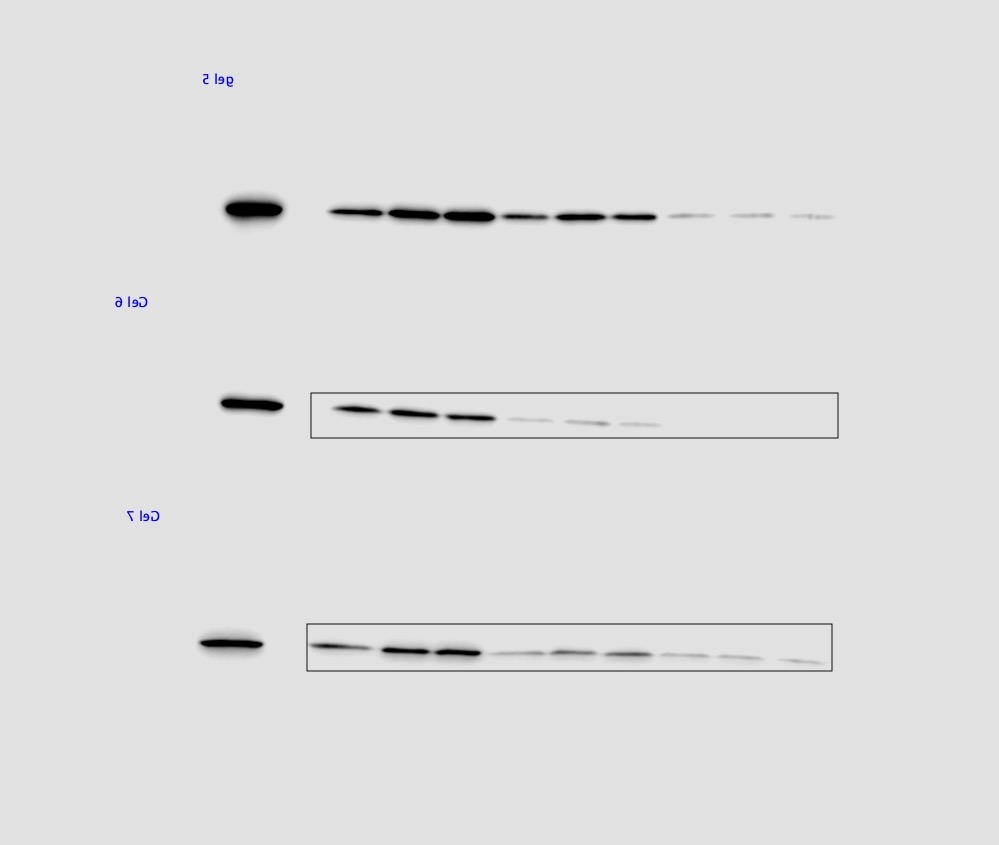

Supplement: Figure 6—source data 2. [file elife-97577-fig6-data2.zip › Figure6D_SourceData2/Fig6D_Box_NR6_COS7_H3K27me3.jpeg]

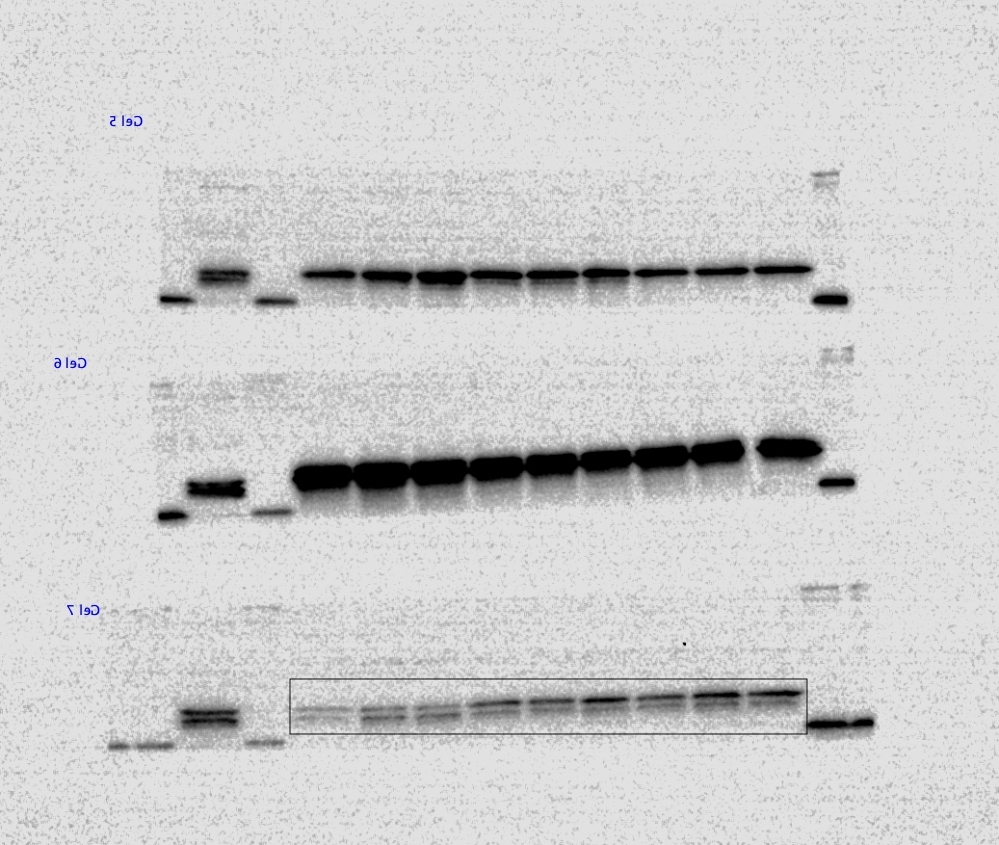

Supplement: Figure 6—source data 2. [file elife-97577-fig6-data2.zip › Figure6D_SourceData2/Fig6D_Box_COS7_CCND1.jpeg]

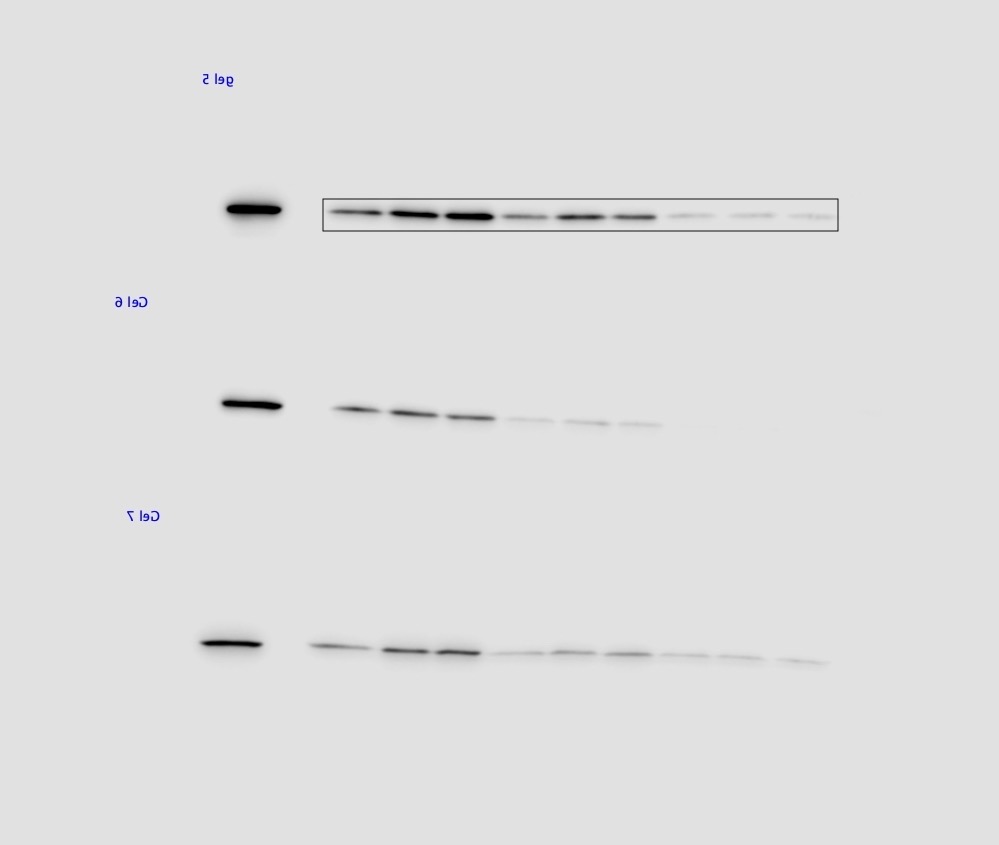

Supplement: Figure 6—source data 2. [file elife-97577-fig6-data2.zip › Figure6D_SourceData2/Fig6D_Box_LNCaP-M1-2166_H3K27me3.jpeg]

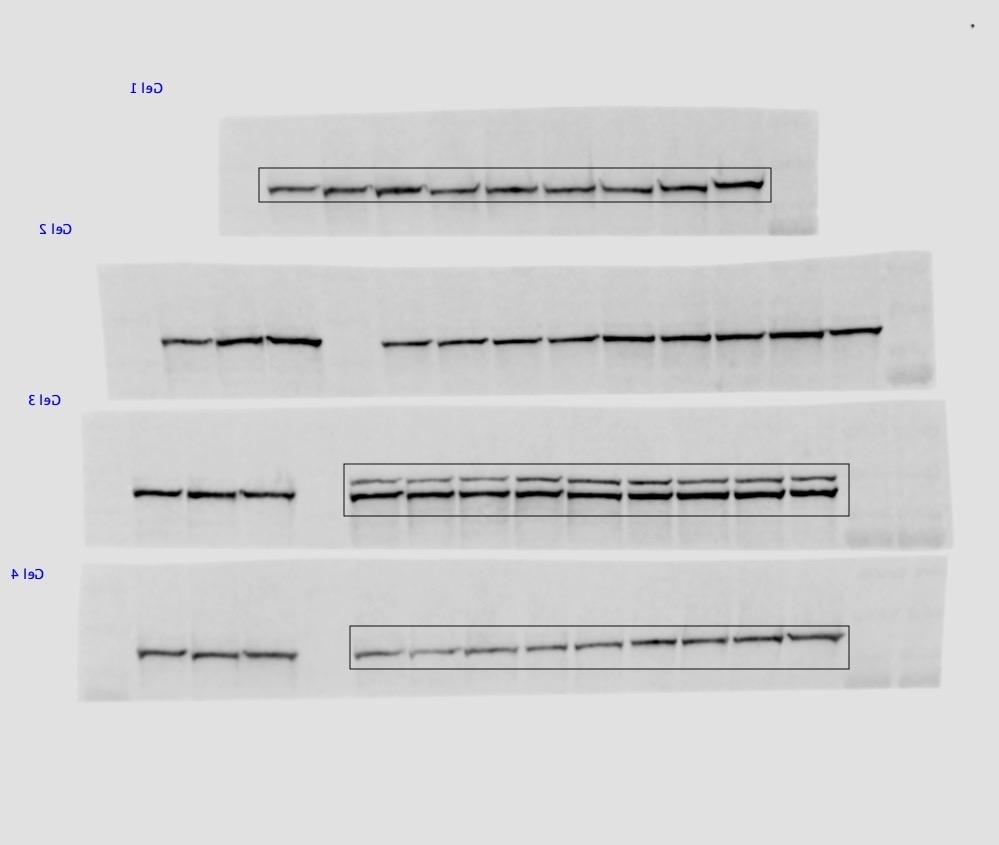

Supplement: Figure 6—source data 2. [file elife-97577-fig6-data2.zip › Figure6D_SourceData2/Fig6D_Box_HAP1_MDA-MB-231_KBM7_Vinc.jpeg]

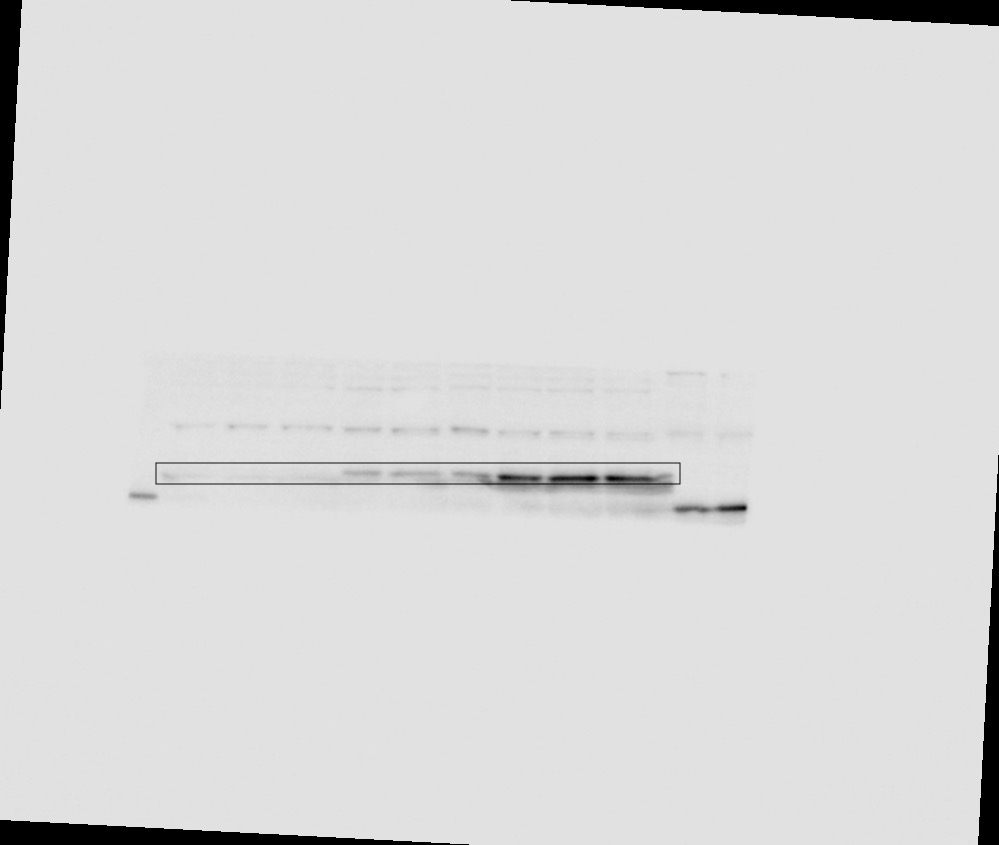

Supplement: Figure 6—source data 2. [file elife-97577-fig6-data2.zip › Figure6D_SourceData2/Fig6D_Box_KBM7_CCND1.jpeg]

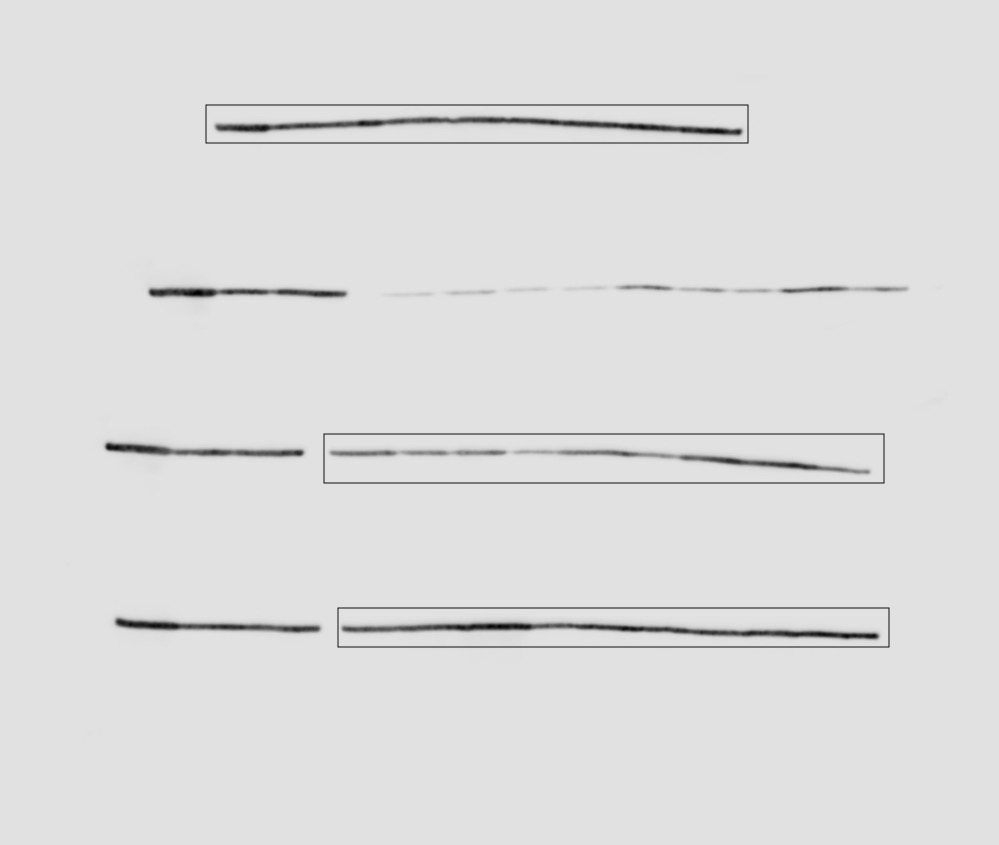

Supplement: Figure 6—source data 2. [file elife-97577-fig6-data2.zip › Figure6D_SourceData2/Fig6D_Box_HAP1_MDA-MB-231_KBM7_H3.jpeg]

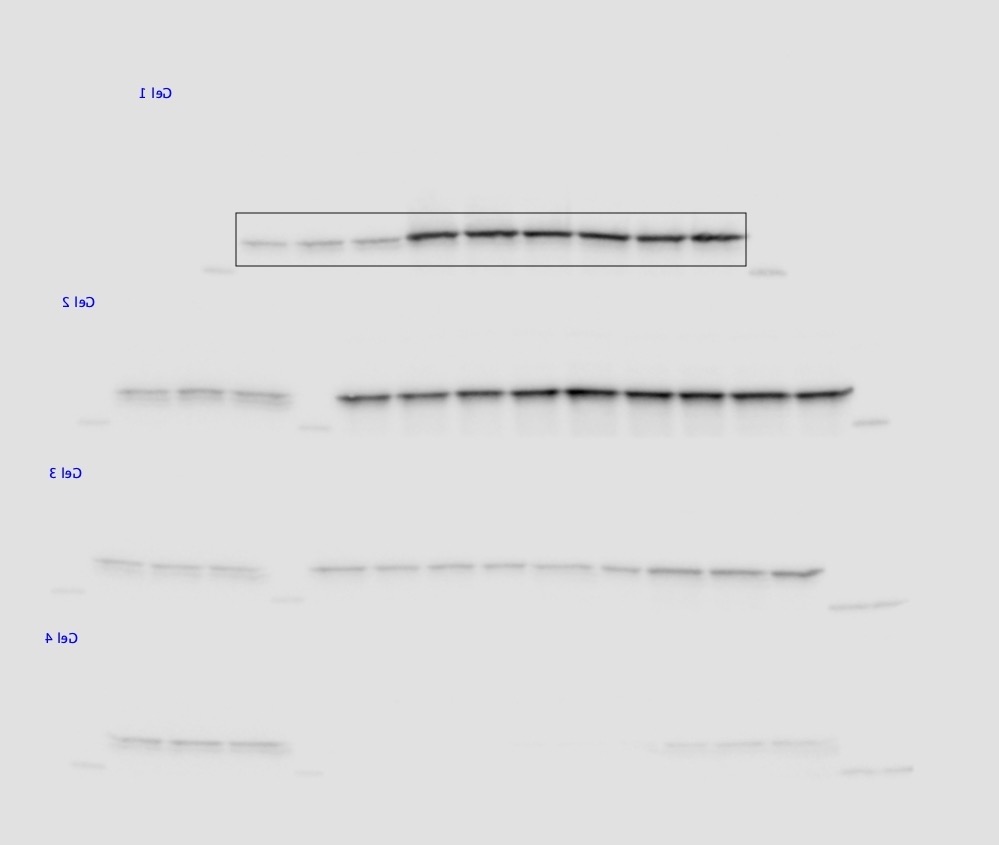

Supplement: Figure 6—source data 2. [file elife-97577-fig6-data2.zip › Figure6D_SourceData2/Fig6D_Box_HAP1_CCND1.jpeg]

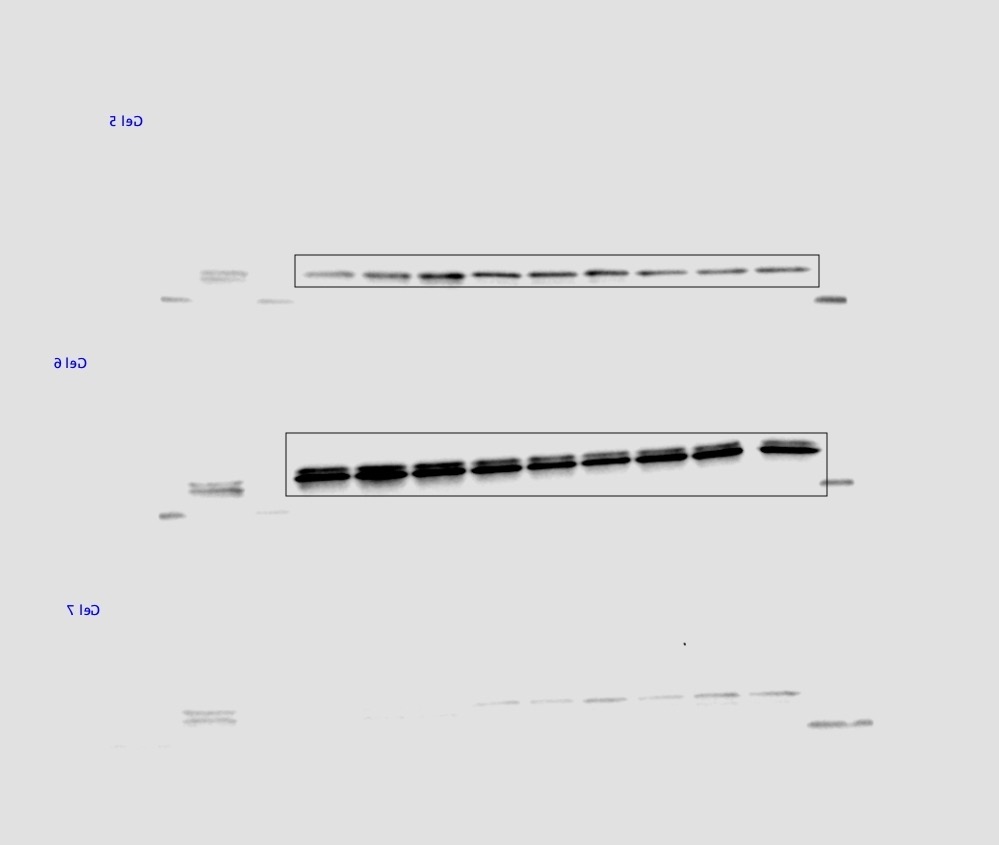

Supplement: Figure 6—source data 2. [file elife-97577-fig6-data2.zip › Figure6D_SourceData2/Fig6D_Box_LNCaP-M1-2166_NR6_CCND1.jpeg]

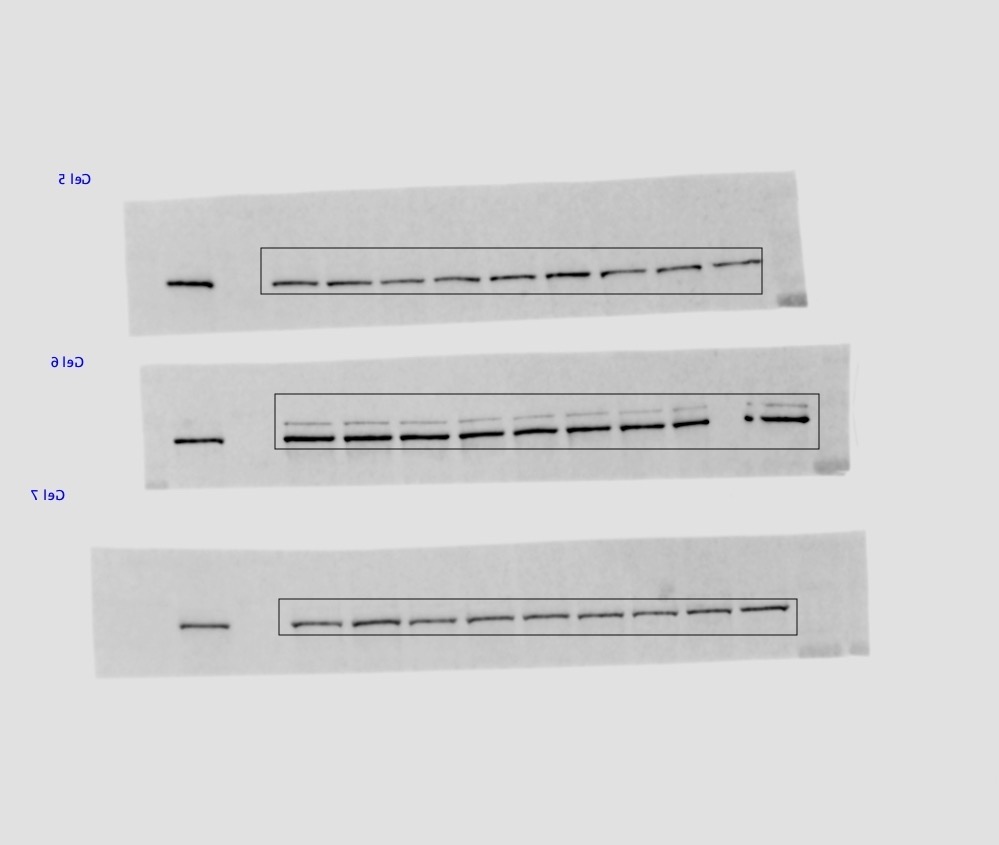

Supplement: Figure 6—source data 2. [file elife-97577-fig6-data2.zip › Figure6D_SourceData2/Fig6D_Box_LNCaP-M1-2166_NR6_COS7_Vinc.jpeg]

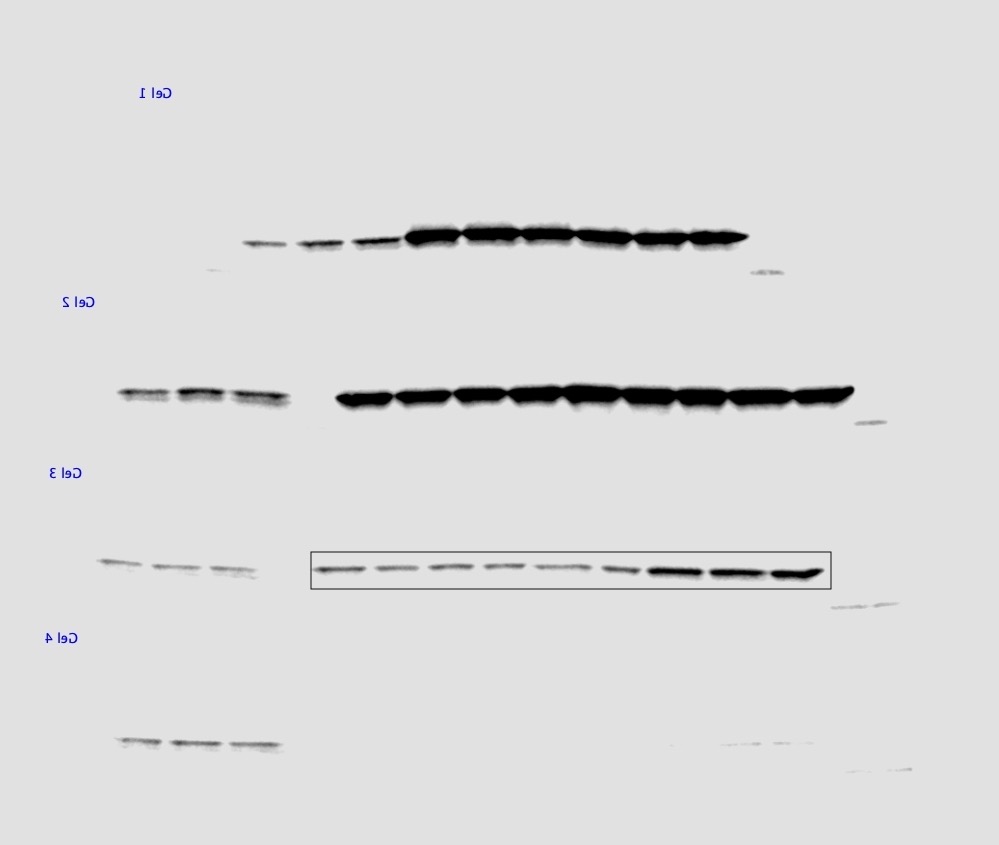

Supplement: Figure 6—source data 2. [file elife-97577-fig6-data2.zip › Figure6D_SourceData2/Fig6D_Box_MDA-MB-231_CCND1.jpeg]

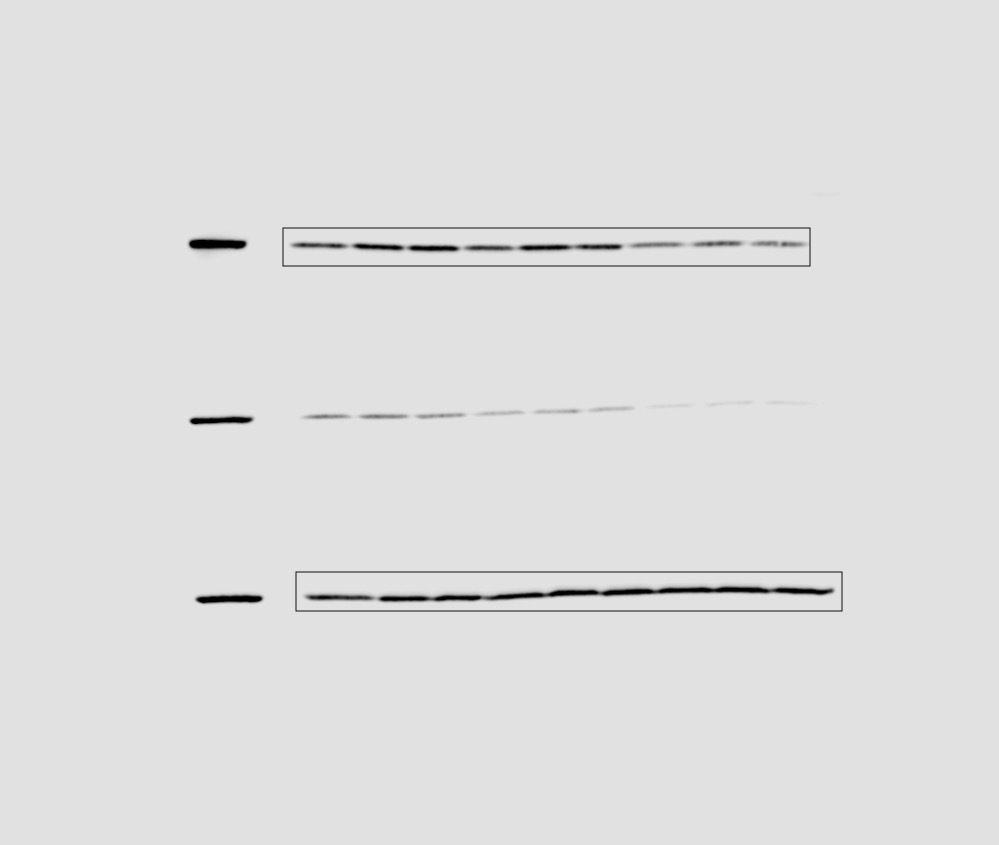

Supplement: Figure 6—source data 2. [file elife-97577-fig6-data2.zip › Figure6D_SourceData2/Fig6D_Box_LNCaP_COS7_H3.jpeg]

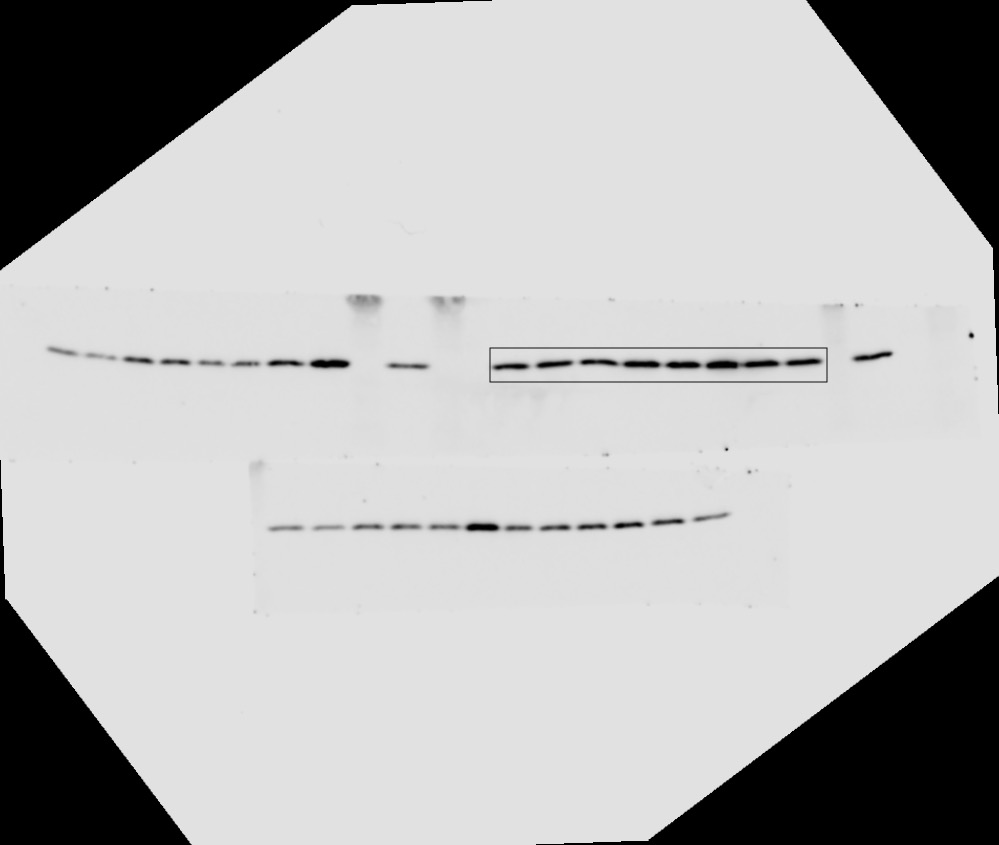

Supplement: Figure 6—source data 2. [file elife-97577-fig6-data2.zip › Figure6E_SourceData2/Fig6E_Box_COS7_H3.jpeg]

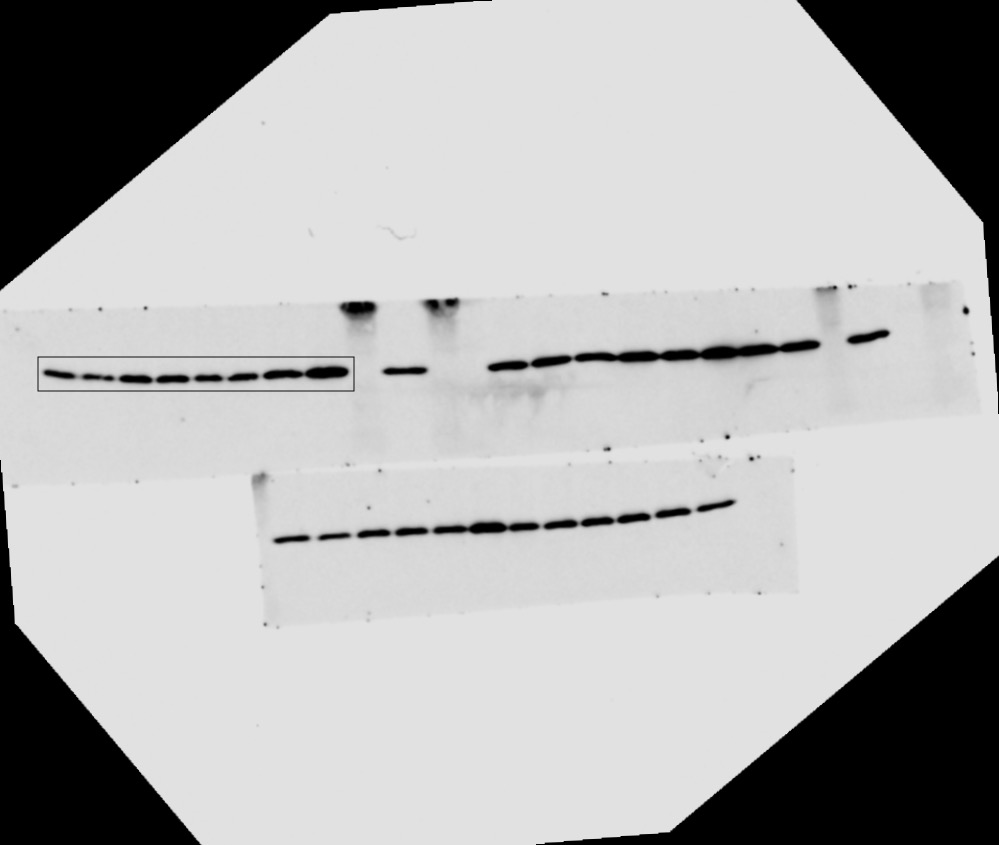

Supplement: Figure 6—source data 2. [file elife-97577-fig6-data2.zip › Figure6E_SourceData2/Fig6E_Box_MDA-MB-231_H3.jpeg]

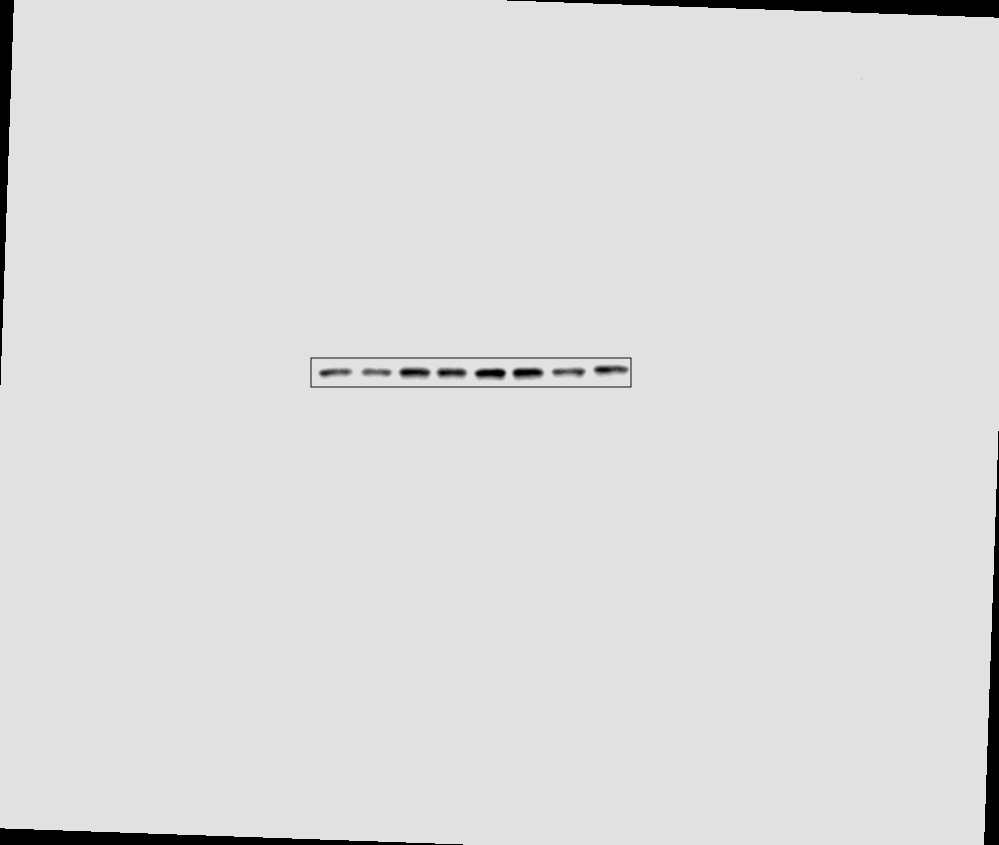

Supplement: Figure 6—source data 2. [file elife-97577-fig6-data2.zip › Figure6E_SourceData2/Fig6E_Box_MDA-MB-231_CCND1.jpeg]

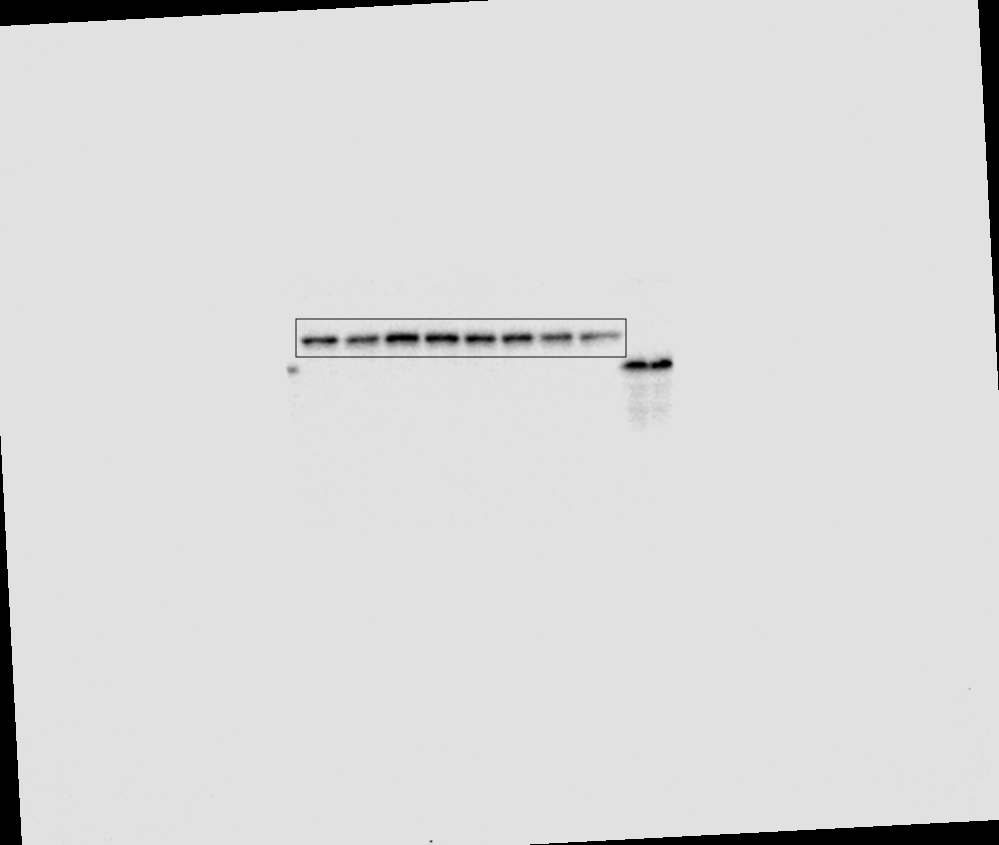

Supplement: Figure 6—source data 2. [file elife-97577-fig6-data2.zip › Figure6E_SourceData2/Fig6E_Box_COS7_CCND1.jpeg]

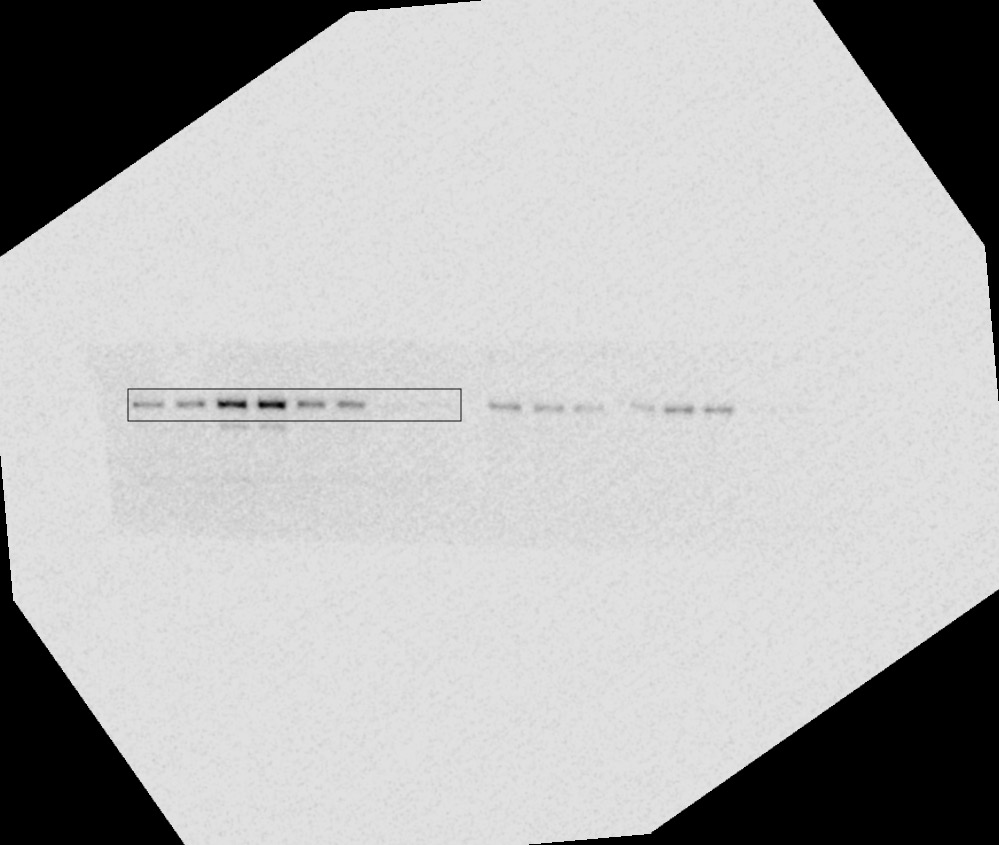

Supplement: Figure 6—source data 2. [file elife-97577-fig6-data2.zip › Figure6E_SourceData2/Fig6E_Box_MDA-MB-231_JARID2.jpeg]

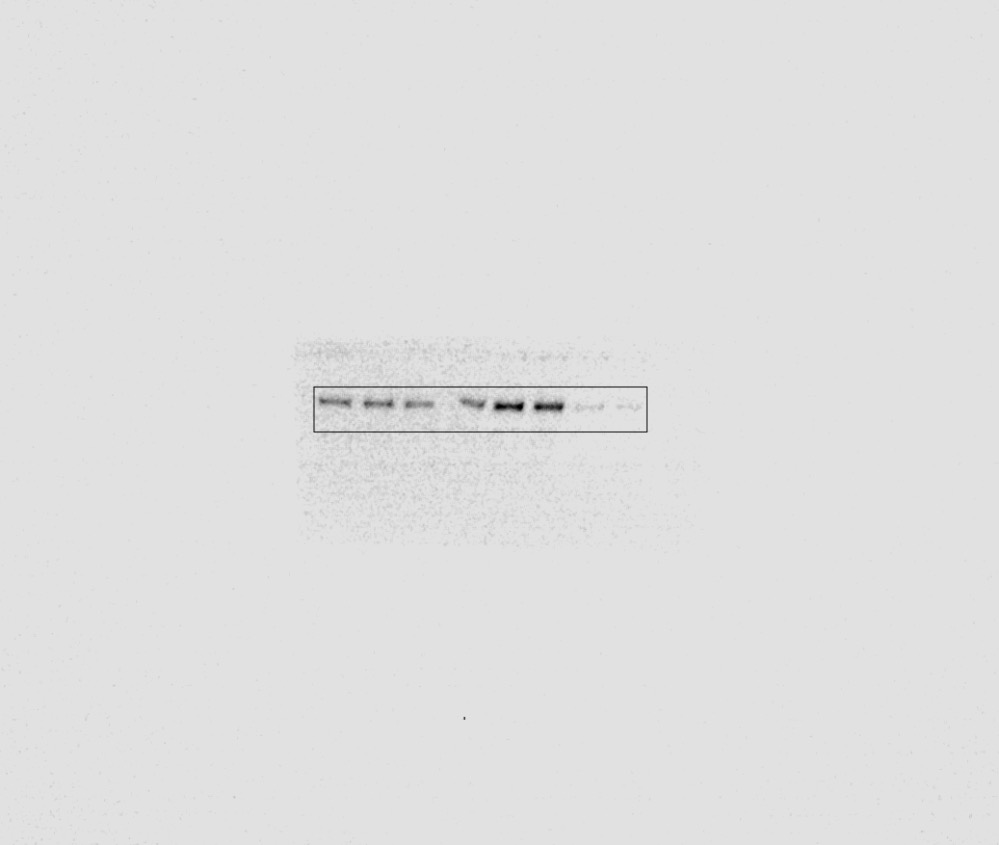

Supplement: Figure 6—source data 2. [file elife-97577-fig6-data2.zip › Figure6E_SourceData2/Fig6E_Box_COS7_JARID2.jpeg]

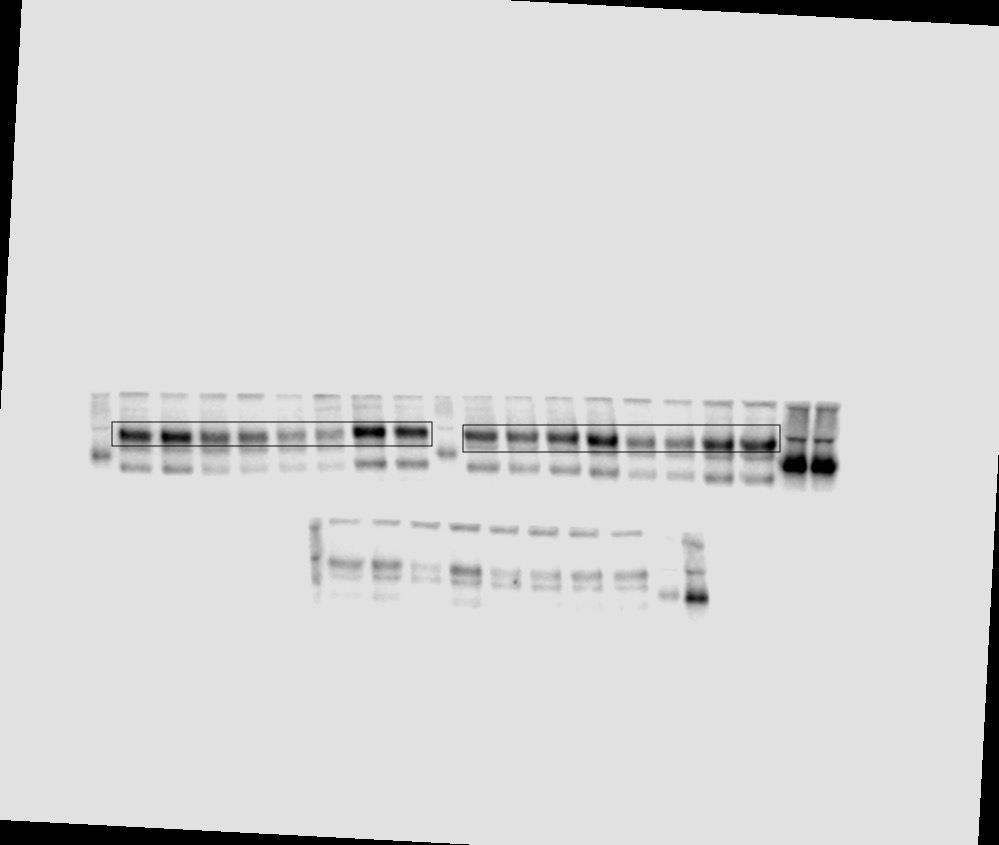

Supplement: Figure 6—source data 2. [file elife-97577-fig6-data2.zip › Figure6E_SourceData2/Fig6E_Box_MDA-MB-231_COS7_MTF2.jpeg]

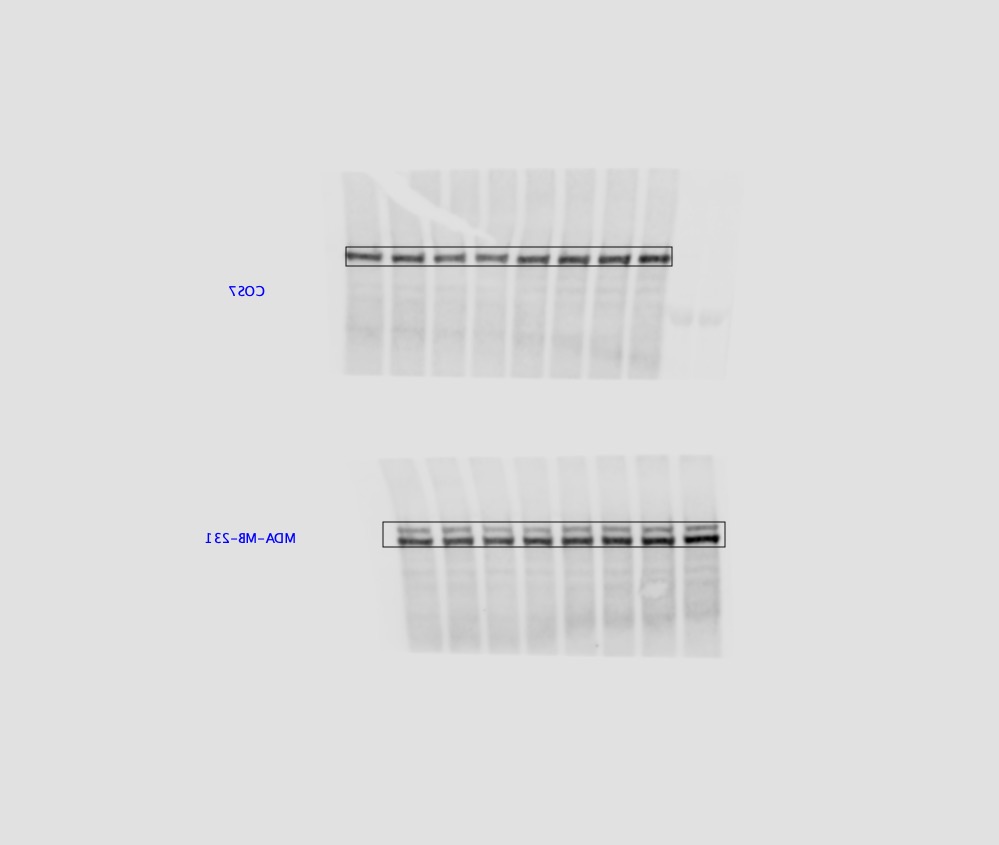

Supplement: Figure 6—source data 2. [file elife-97577-fig6-data2.zip › Figure6E_SourceData2/Fig6E_Box_COS7_MDA-MB-231_Vinc.jpeg]

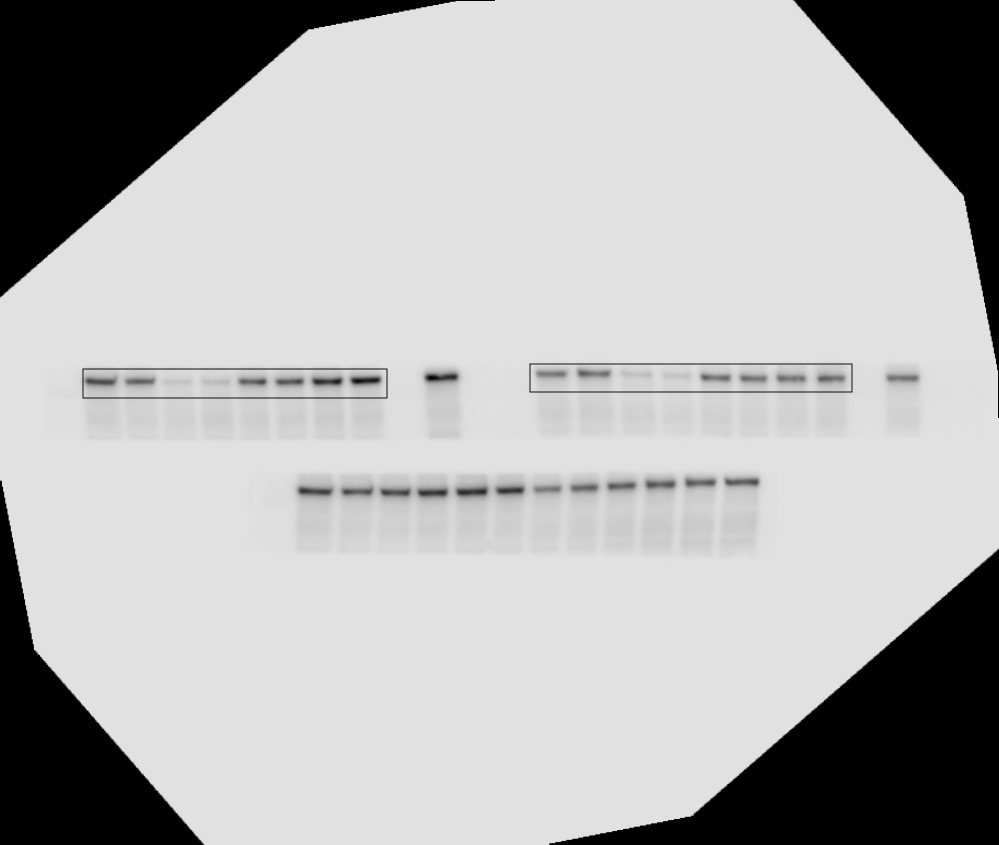

Supplement: Figure 6—source data 2. [file elife-97577-fig6-data2.zip › Figure6E_SourceData2/Fig6E_Box_MDA-MB-231_COS7_SUZ12.jpeg]

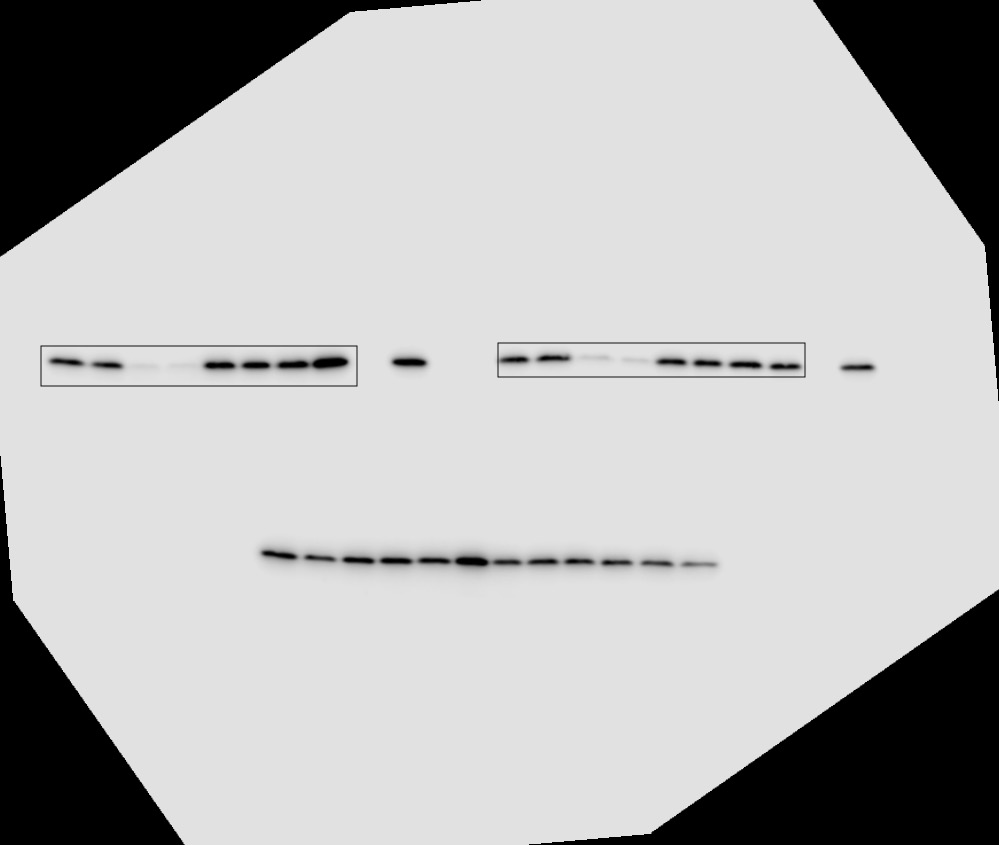

Supplement: Figure 6—source data 2. [file elife-97577-fig6-data2.zip › Figure6E_SourceData2/Fig6E_Box_COS7_MDA-MB-231_H3K27me3.jpeg]

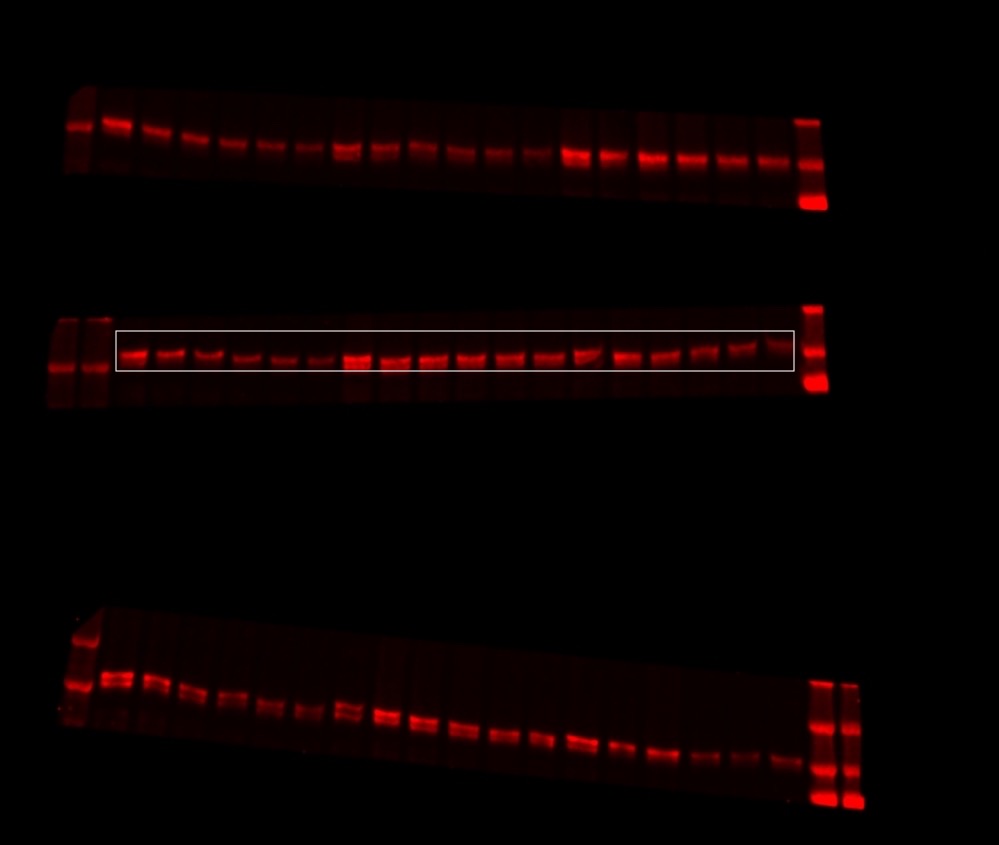

Supplement: Figure 6—source data 2. [file elife-97577-fig6-data2.zip › Figure6F_SourceData2/811_RB1.jpeg]

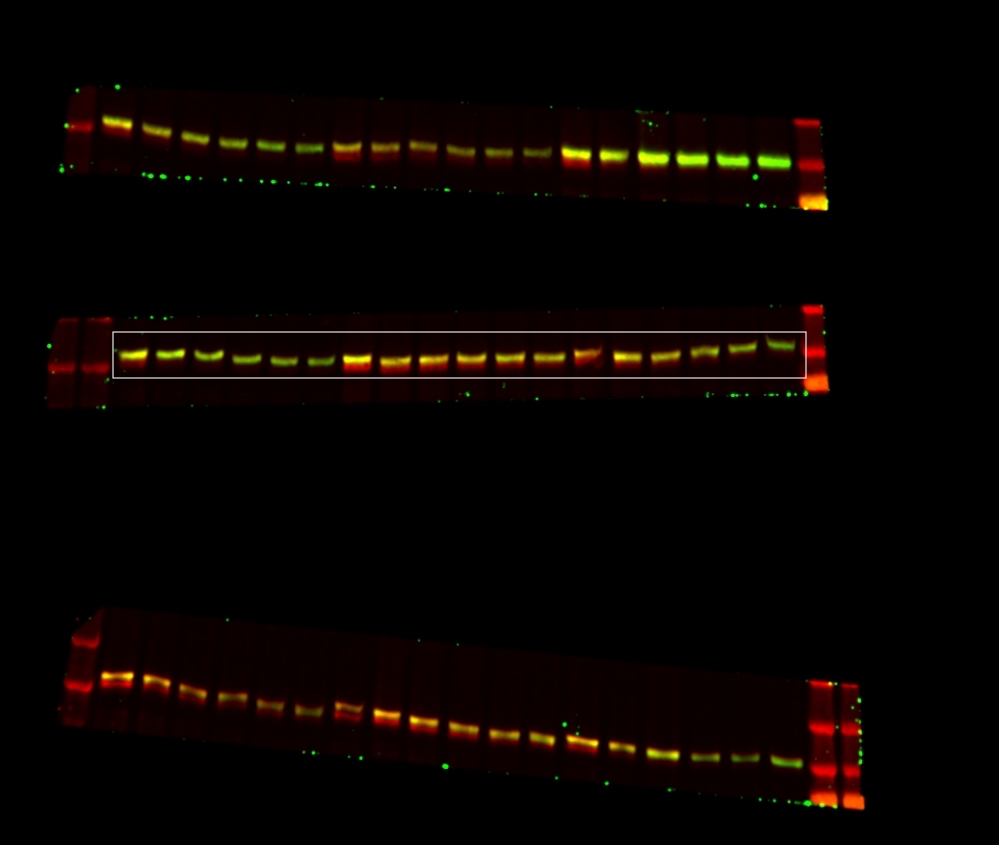

Supplement: Figure 6—source data 2. [file elife-97577-fig6-data2.zip › Figure6F_SourceData2/Fig6F_Box_P-RB1_RB1_Composite.jpeg]

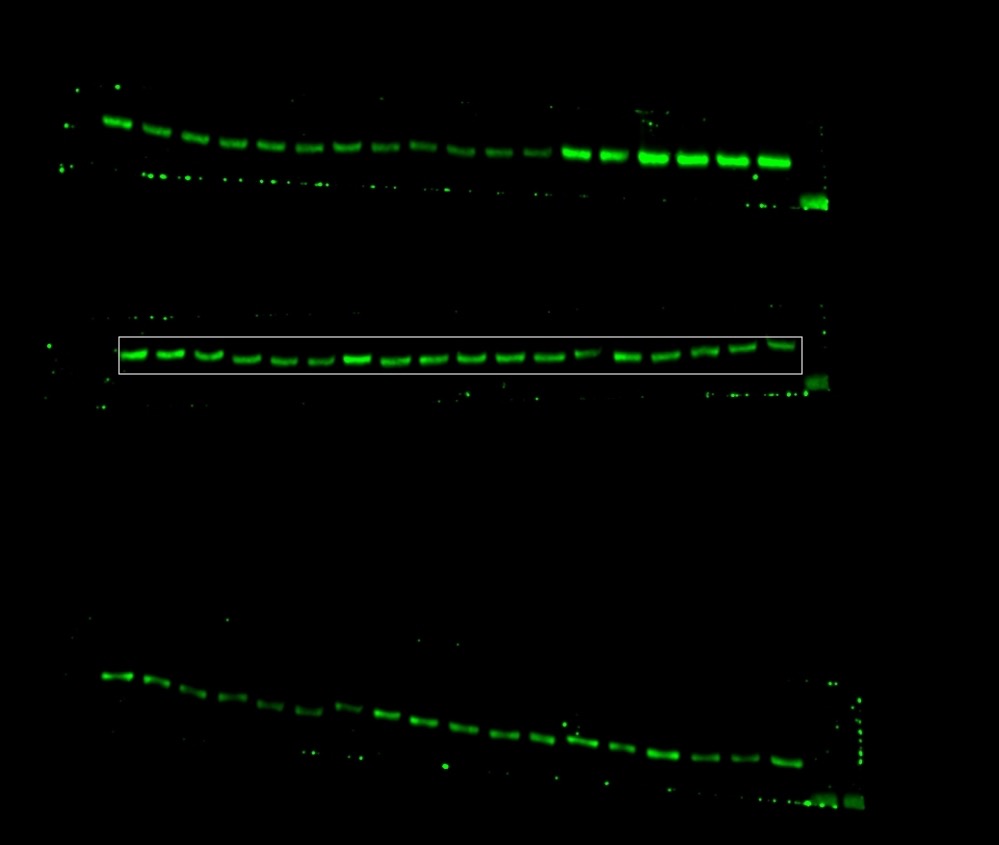

Supplement: Figure 6—source data 2. [file elife-97577-fig6-data2.zip › Figure6F_SourceData2/Fig6F_Box_RB1.jpeg]

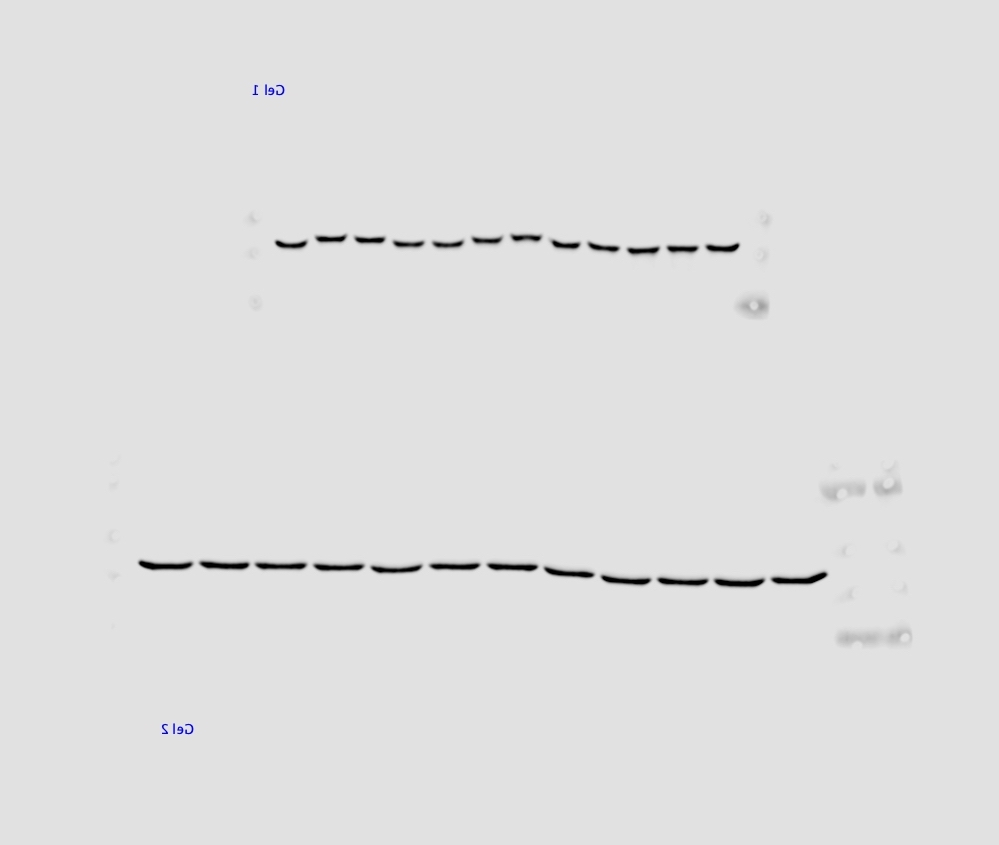

Supplement: Figure 6—figure supplement 2—source data 1. [file elife-97577-fig6-figsupp2-data1.zip › FigureS5B_SourceData1/FigS5B_Actin.jpeg]

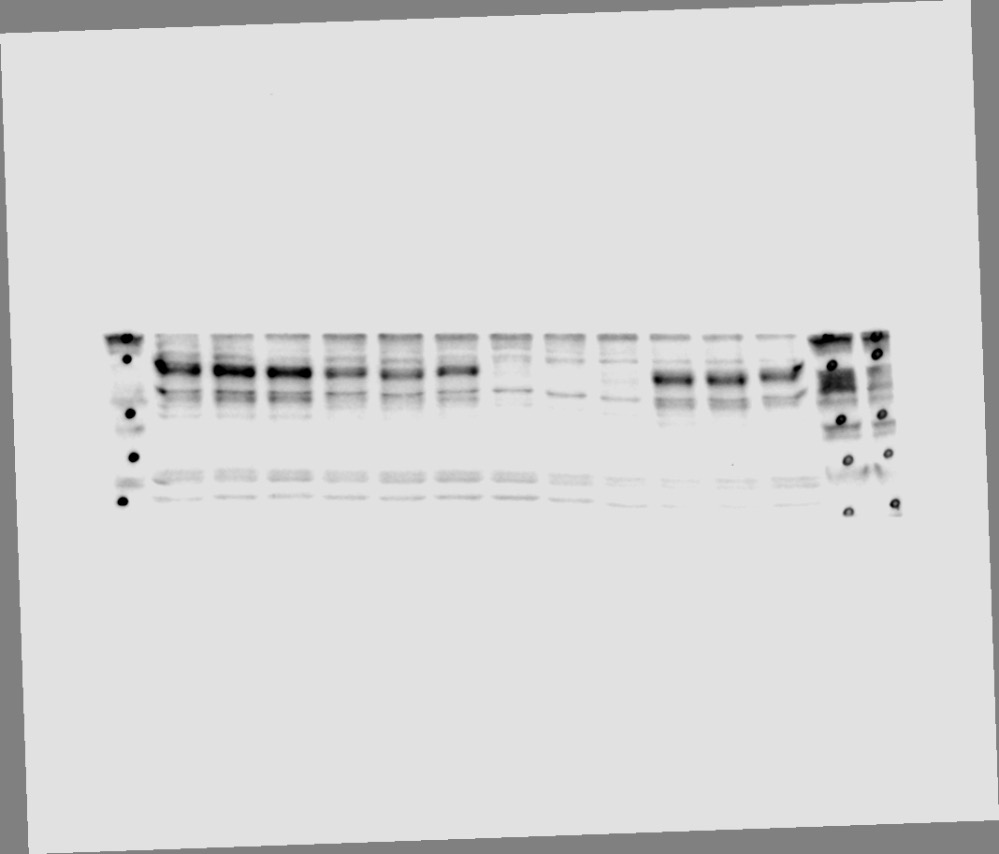

Supplement: Figure 6—figure supplement 2—source data 1. [file elife-97577-fig6-figsupp2-data1.zip › FigureS5B_SourceData1/FigS5B_MTF2.jpeg]

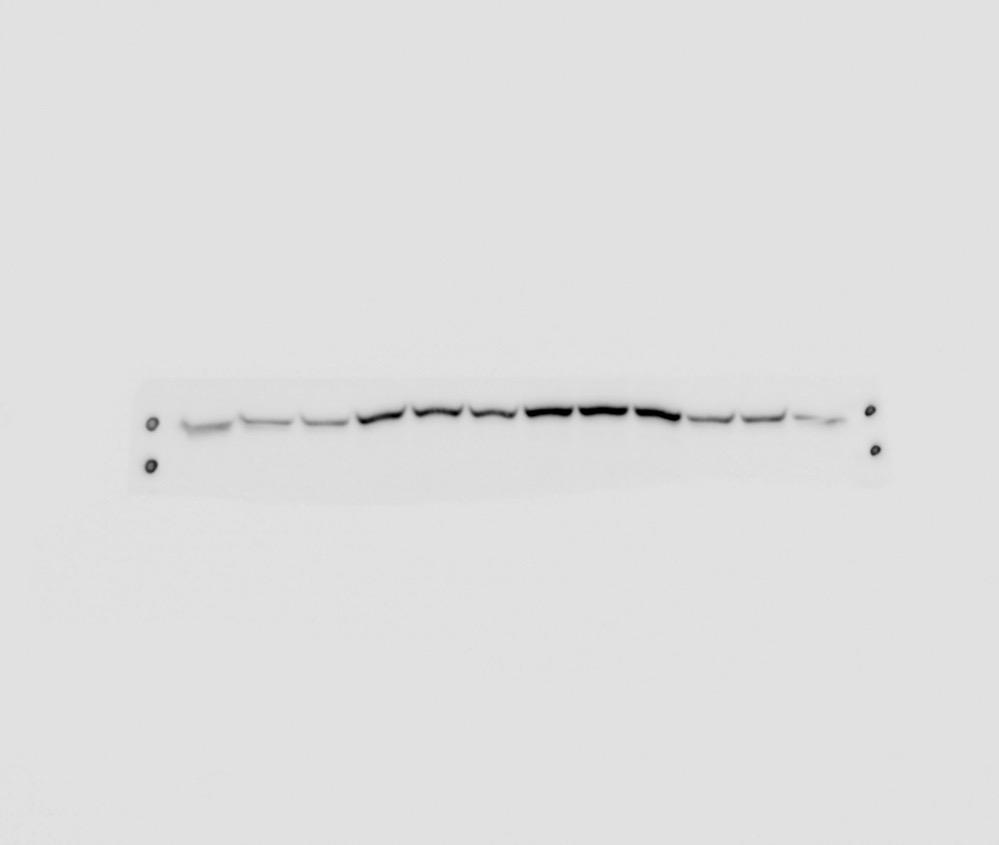

Supplement: Figure 6—figure supplement 2—source data 1. [file elife-97577-fig6-figsupp2-data1.zip › FigureS5B_SourceData1/FigS5B_CCND2.jpeg]

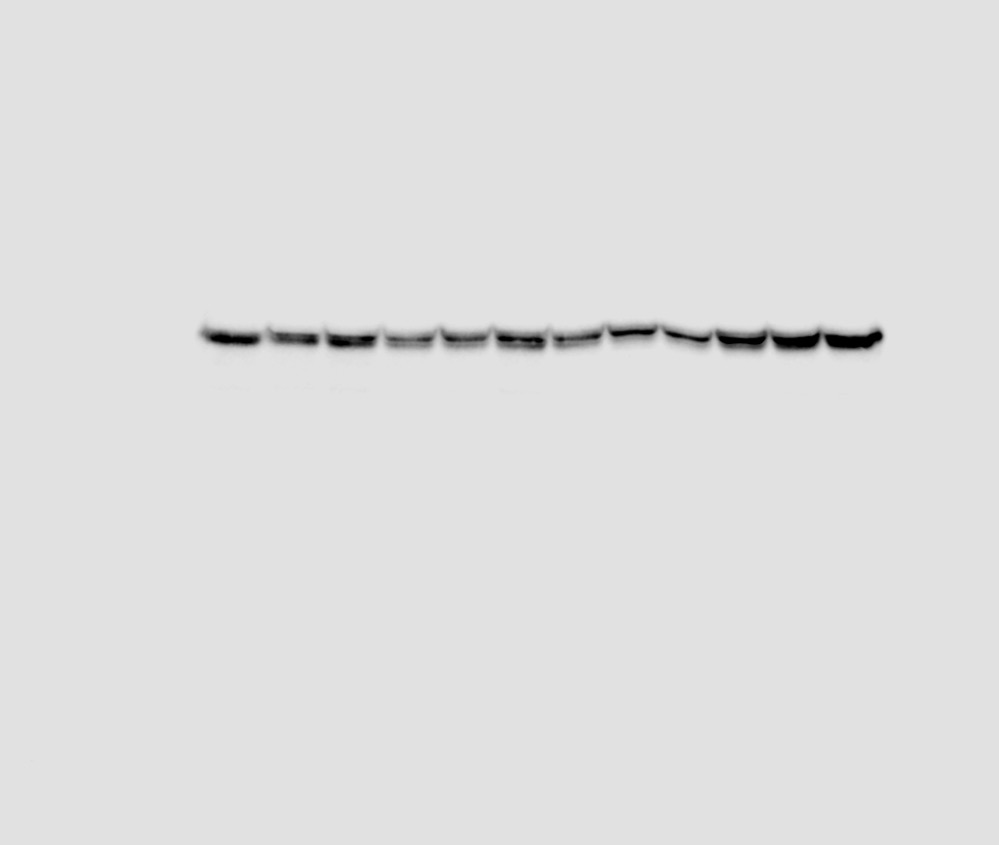

Supplement: Figure 6—figure supplement 2—source data 1. [file elife-97577-fig6-figsupp2-data1.zip › FigureS5B_SourceData1/FigS5B_CCND3.jpeg]

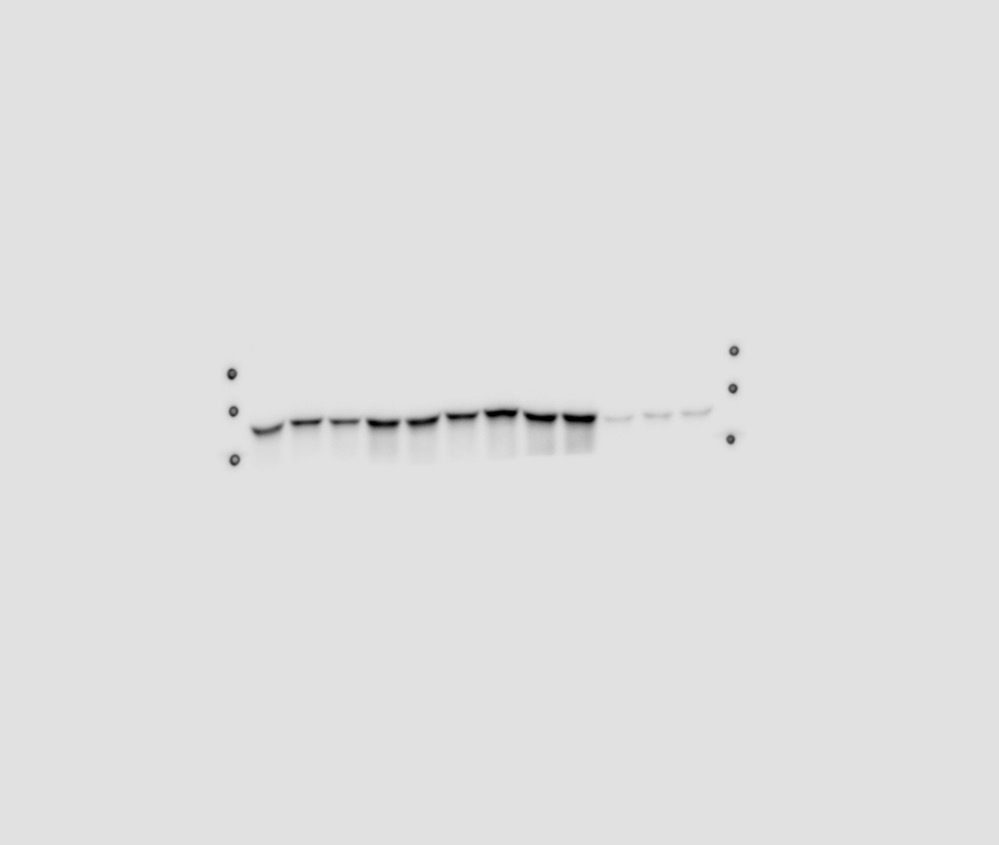

Supplement: Figure 6—figure supplement 2—source data 1. [file elife-97577-fig6-figsupp2-data1.zip › FigureS5B_SourceData1/FigS5B_CCND1.jpeg]
