# Supplementary figures and images for: The PRC2.1 subcomplex opposes G1 progression through regulation of CCND1 and CCND2 (part 2 of 2)
Source: eLife. 2025 Feb 4;13:RP97577. doi: 10.7554/eLife.97577 (PMC11793871; doi:10.7554/eLife.97577)

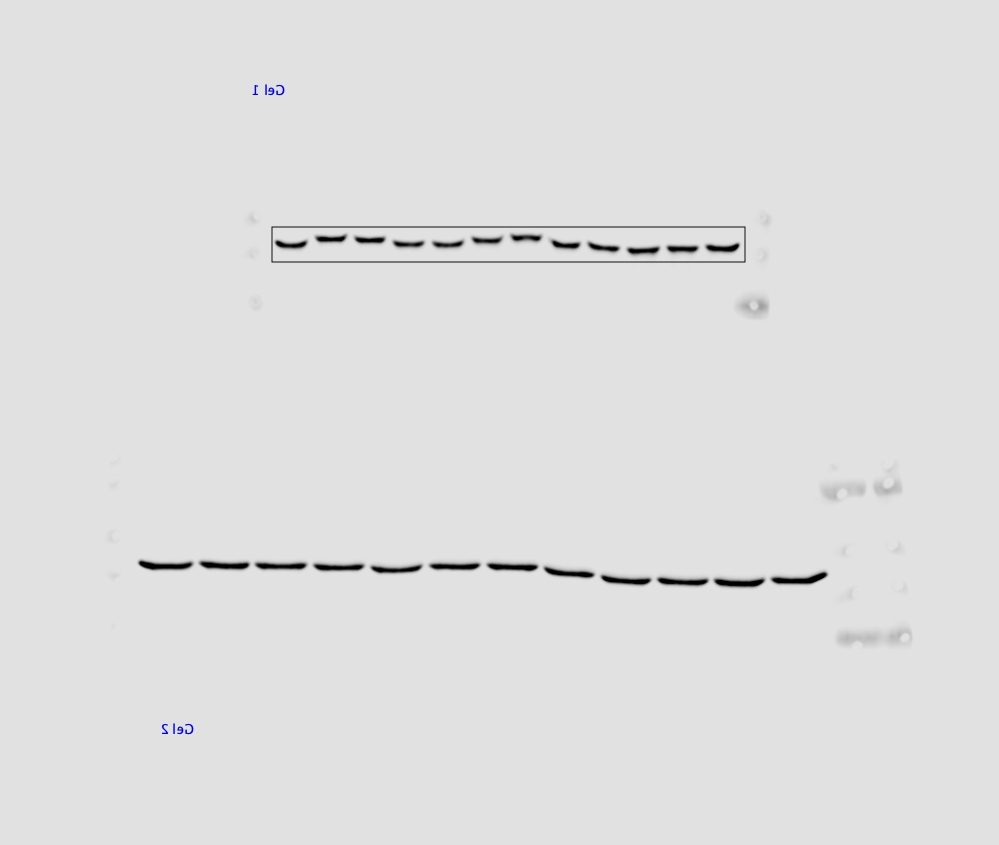

Supplement: Figure 6—figure supplement 2—source data 2. [file elife-97577-fig6-figsupp2-data2.zip › FigureS5B_SourceData2/FigS5B_Box_Actin.jpeg]

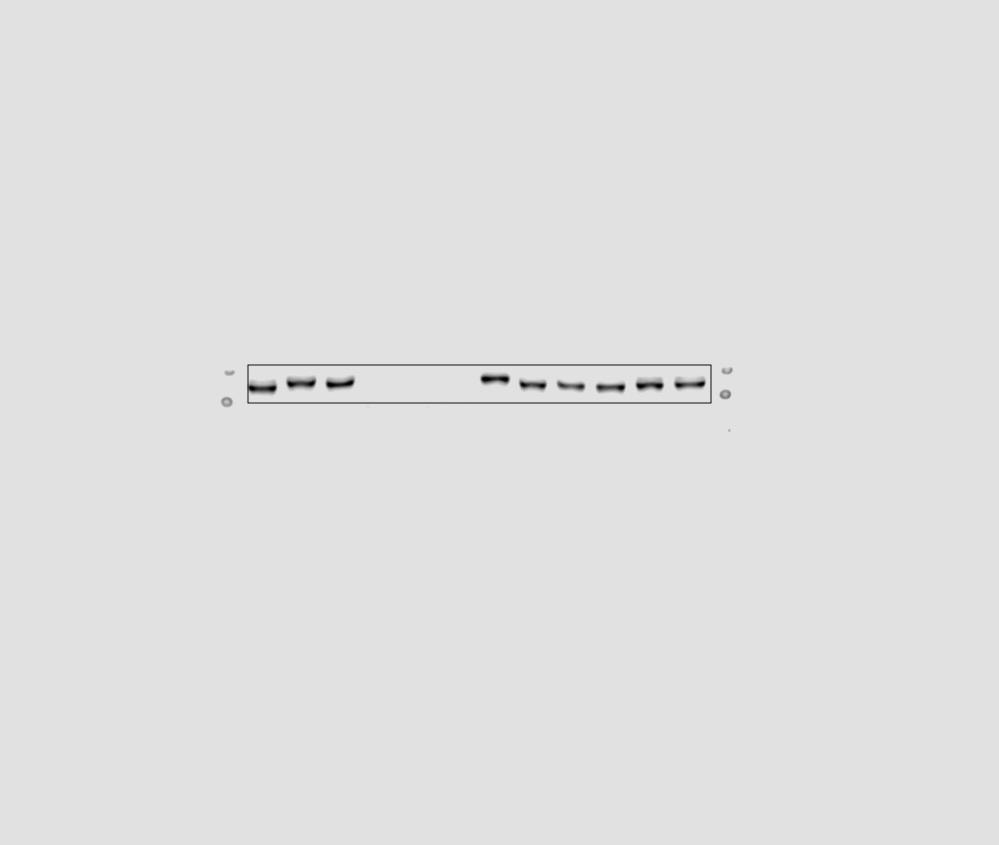

Supplement: Figure 6—figure supplement 2—source data 2. [file elife-97577-fig6-figsupp2-data2.zip › FigureS5B_SourceData2/FigS5B_Box_SUZ12.jpeg]

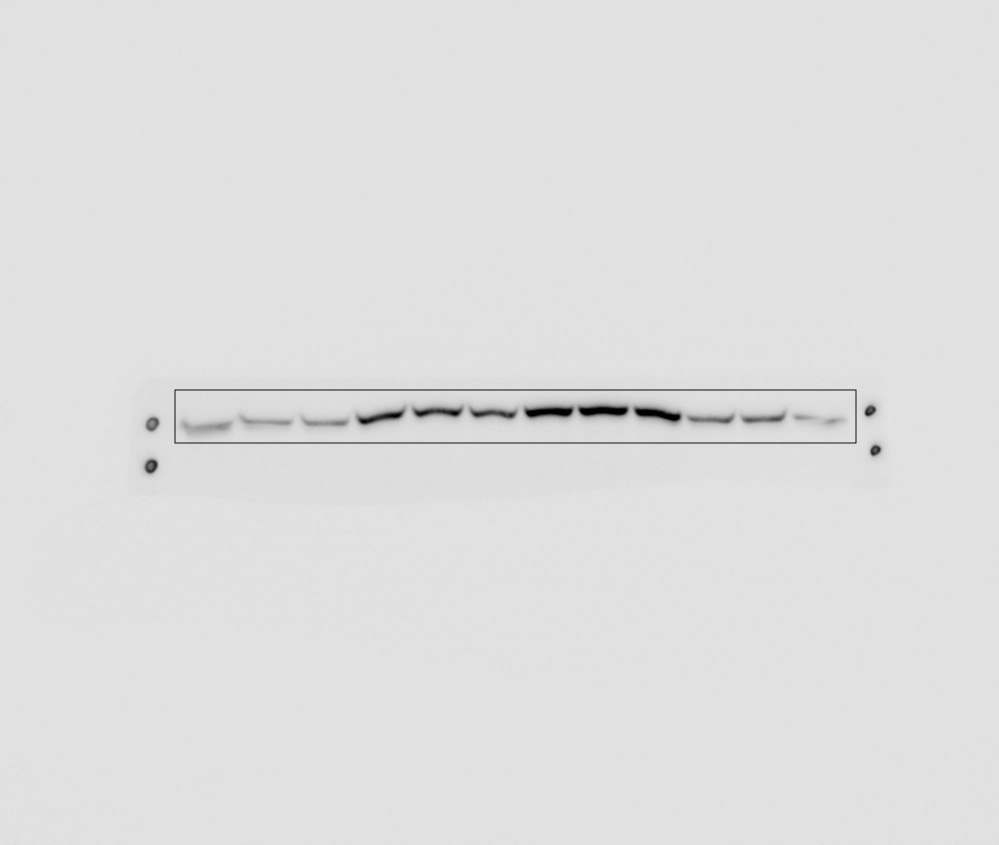

Supplement: Figure 6—figure supplement 2—source data 2. [file elife-97577-fig6-figsupp2-data2.zip › FigureS5B_SourceData2/FigS5B_Box_CCND2.jpeg]

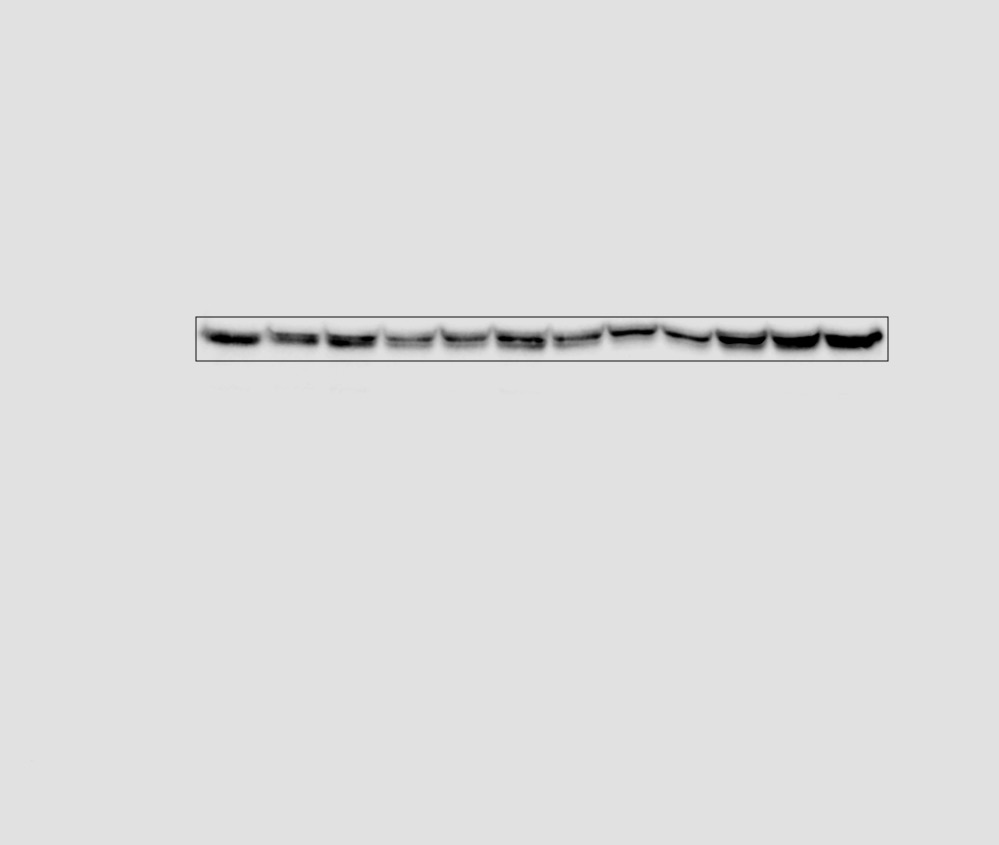

Supplement: Figure 6—figure supplement 2—source data 2. [file elife-97577-fig6-figsupp2-data2.zip › FigureS5B_SourceData2/FigS5B_Box_CCND3.jpeg]

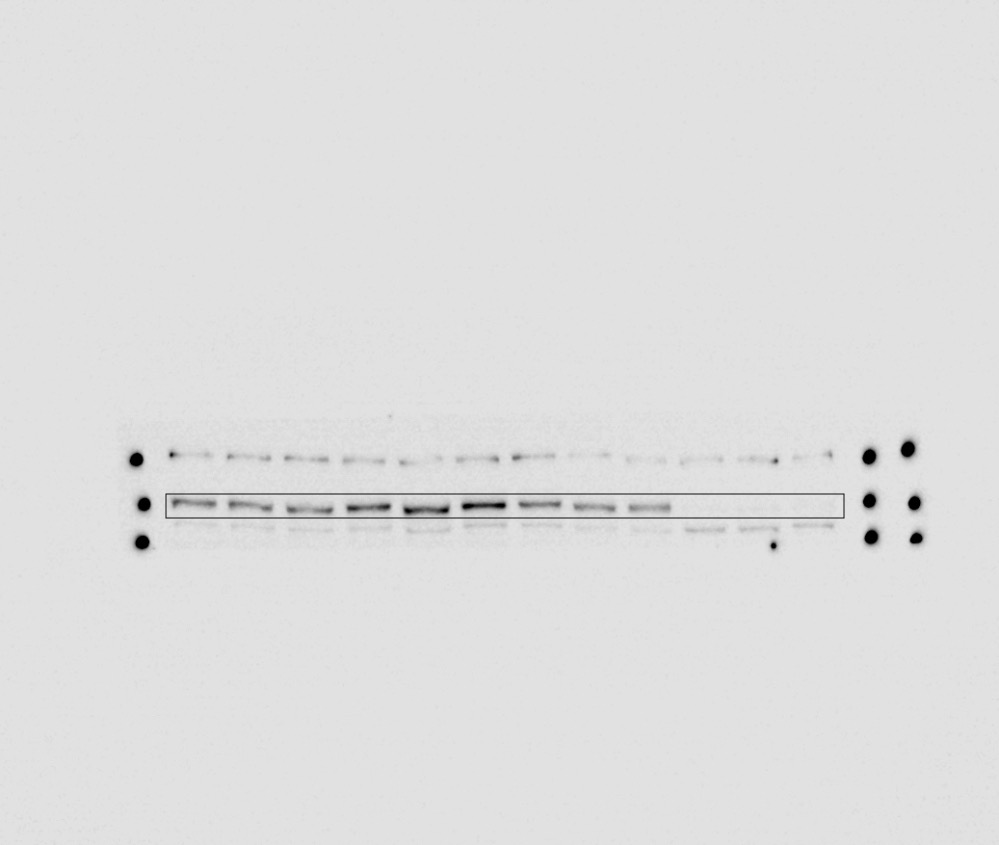

Supplement: Figure 6—figure supplement 2—source data 2. [file elife-97577-fig6-figsupp2-data2.zip › FigureS5B_SourceData2/FigS5B_Box_JARID2.jpeg]

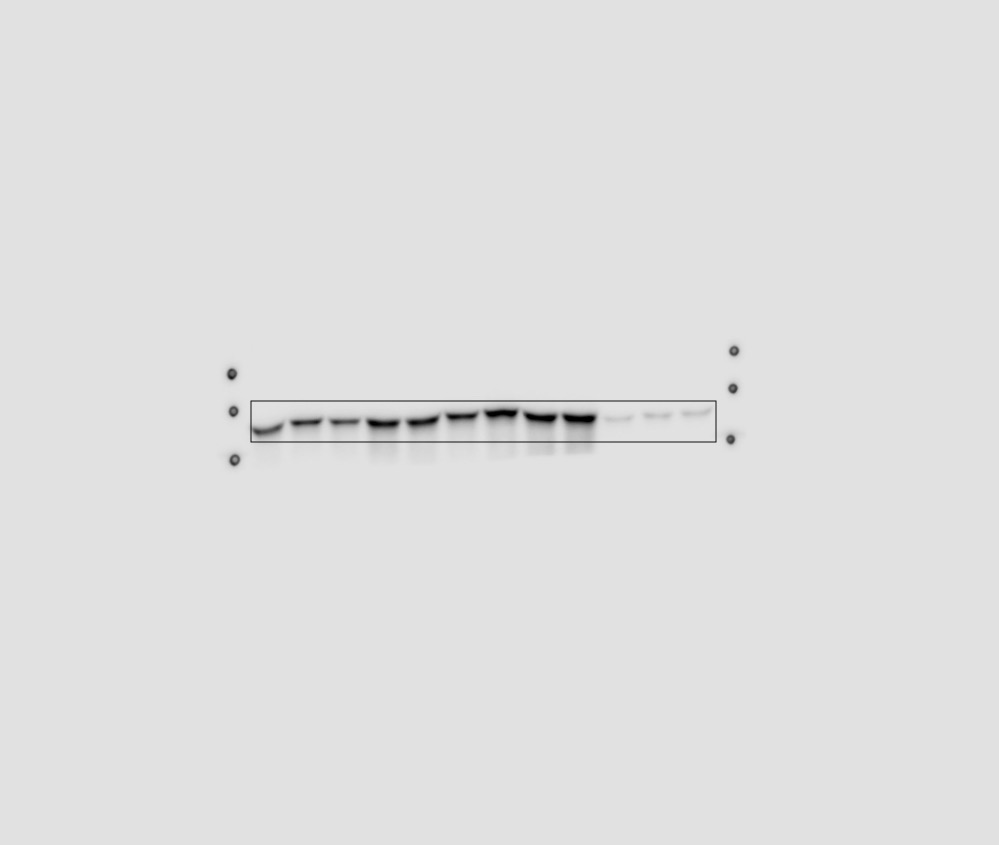

Supplement: Figure 6—figure supplement 2—source data 2. [file elife-97577-fig6-figsupp2-data2.zip › FigureS5B_SourceData2/FigS5B_Box_CCND1.jpeg]

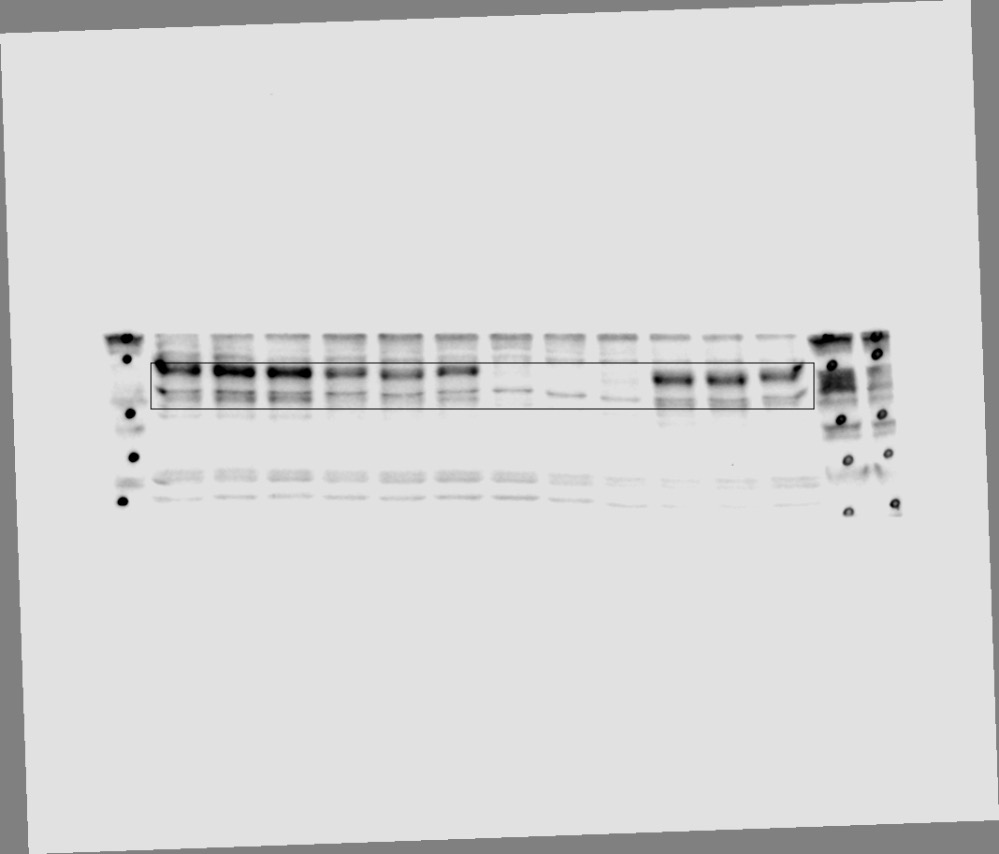

Supplement: Figure 6—figure supplement 2—source data 2. [file elife-97577-fig6-figsupp2-data2.zip › FigureS5B_SourceData2/FigS5B_Box_MTF2.jpeg]

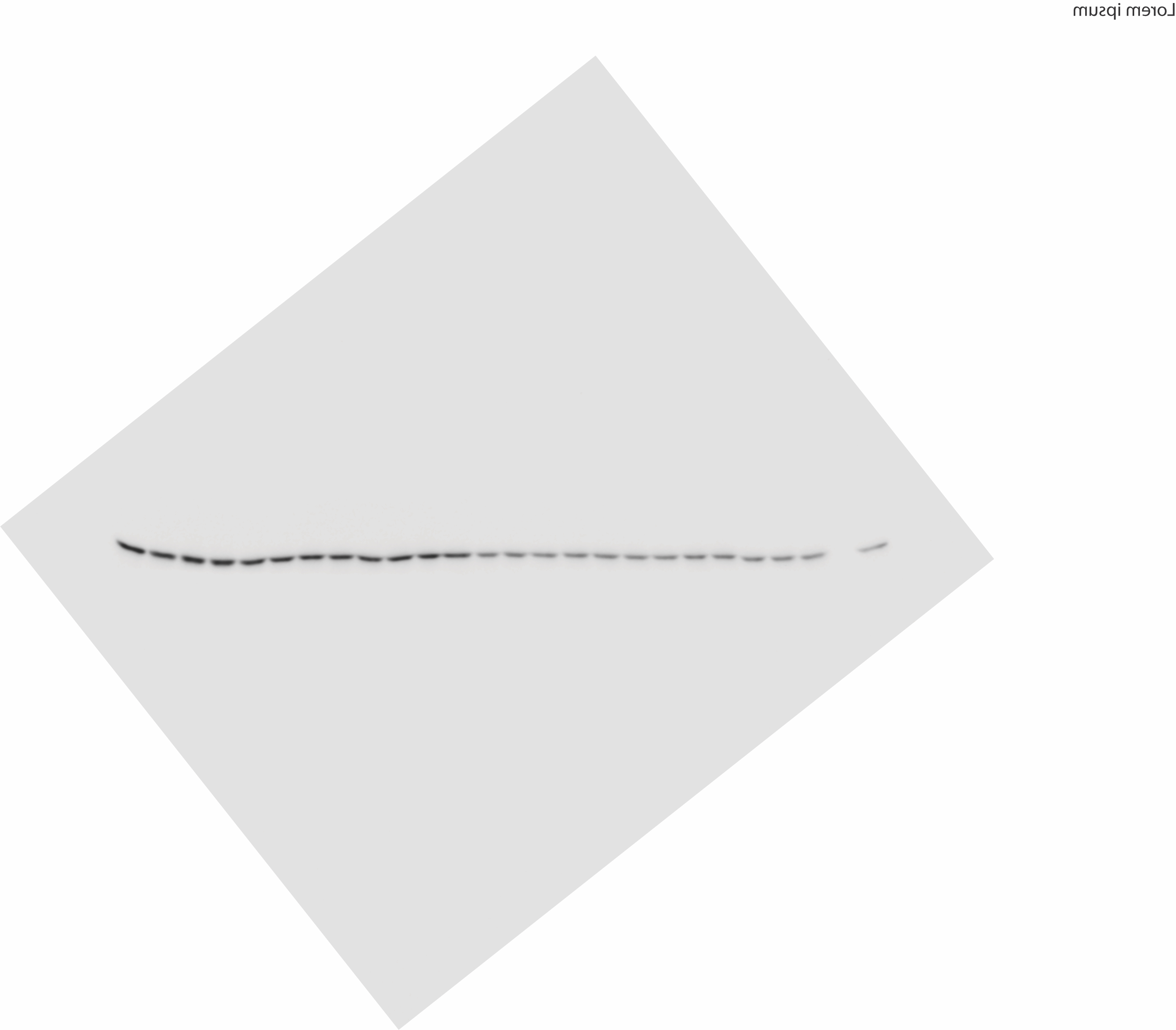

Supplement: Figure 6—figure supplement 3—source data 1. [file elife-97577-fig6-figsupp3-data1.zip › FigureS5E_SourceData1/FigS5E_CDK2.jpeg]

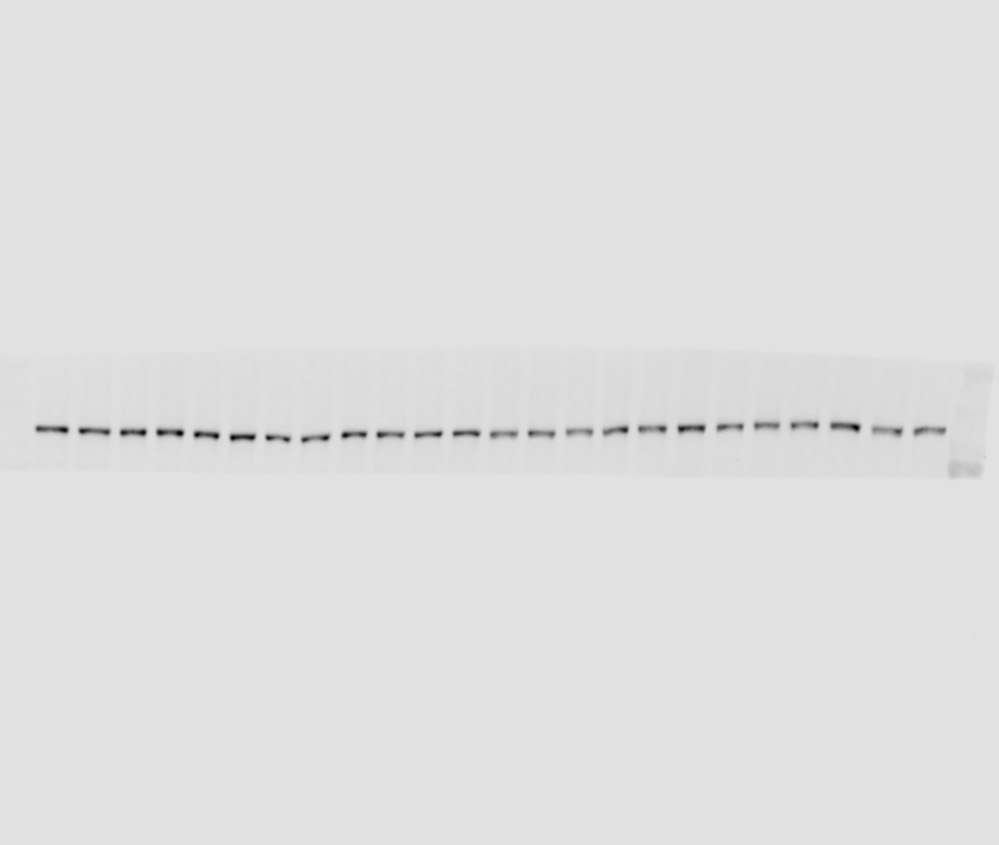

Supplement: Figure 6—figure supplement 3—source data 1. [file elife-97577-fig6-figsupp3-data1.zip › FigureS5E_SourceData1/FigS5E_RB1.jpeg]

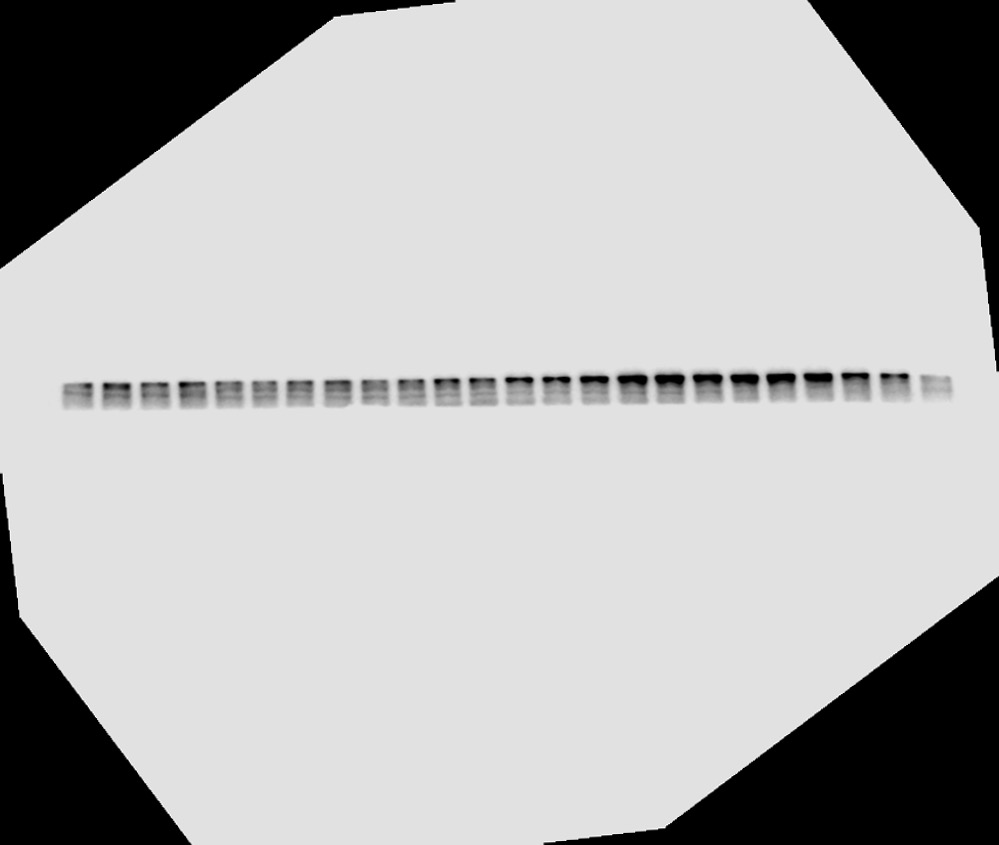

Supplement: Figure 6—figure supplement 3—source data 1. [file elife-97577-fig6-figsupp3-data1.zip › FigureS5E_SourceData1/FigS5E_RBL1.jpeg]

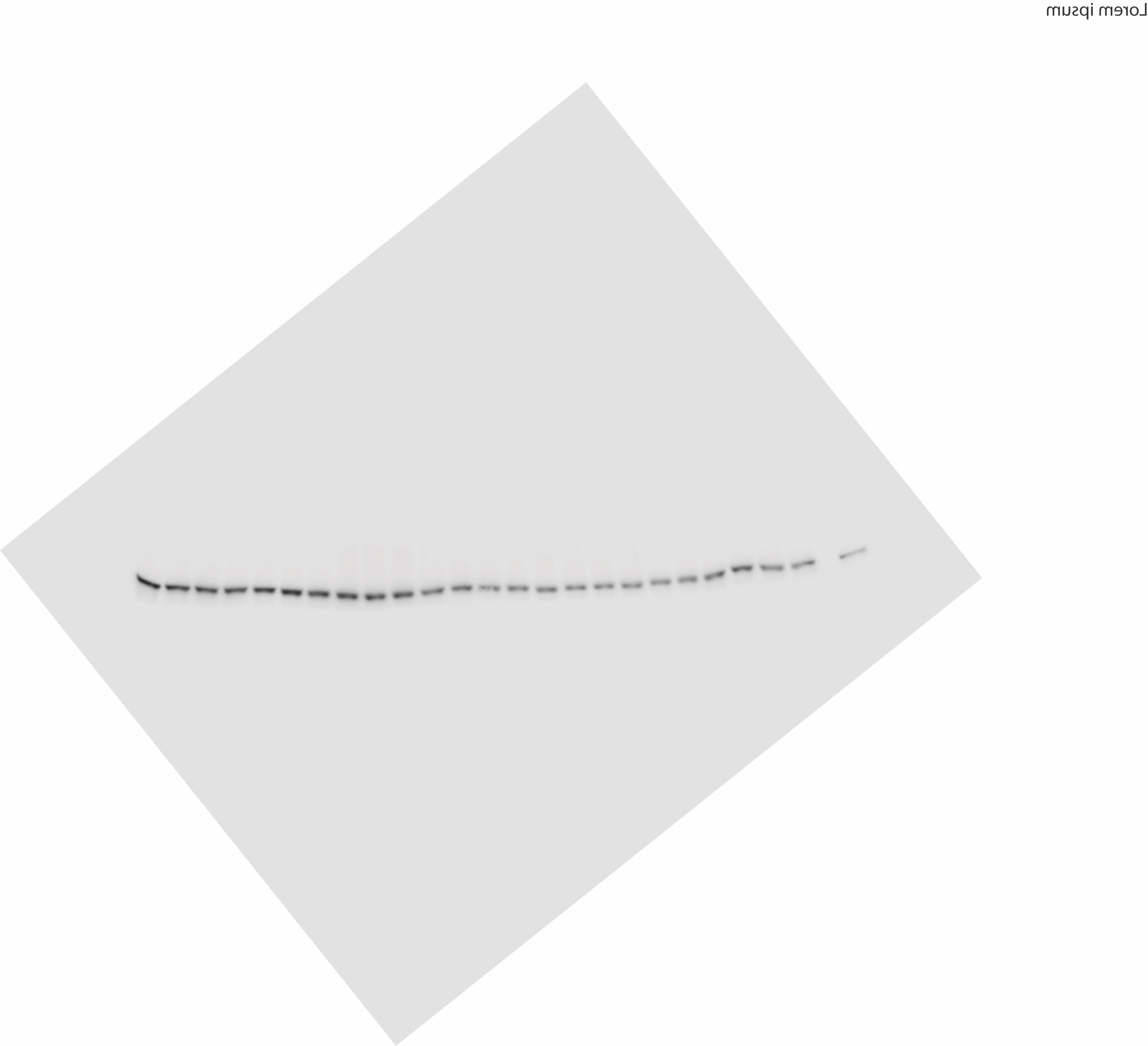

Supplement: Figure 6—figure supplement 3—source data 1. [file elife-97577-fig6-figsupp3-data1.zip › FigureS5E_SourceData1/FigS5E_p53.jpeg]

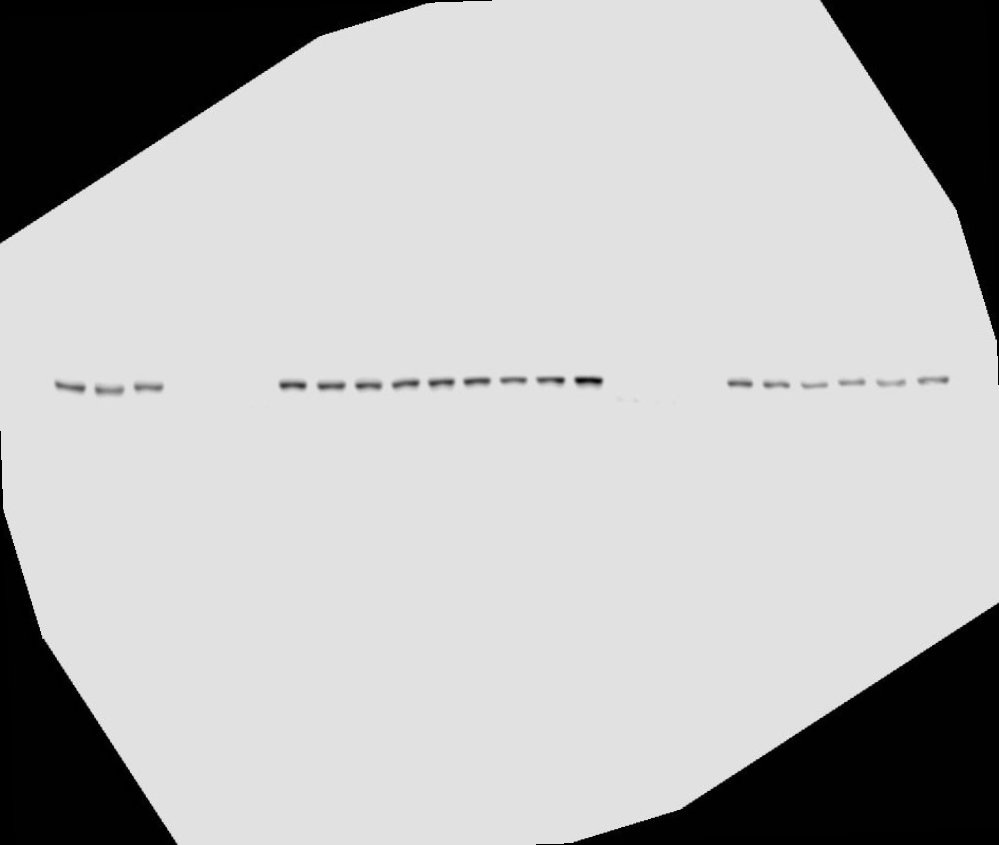

Supplement: Figure 6—figure supplement 3—source data 1. [file elife-97577-fig6-figsupp3-data1.zip › FigureS5E_SourceData1/FigS5E_SUZ12.jpeg]

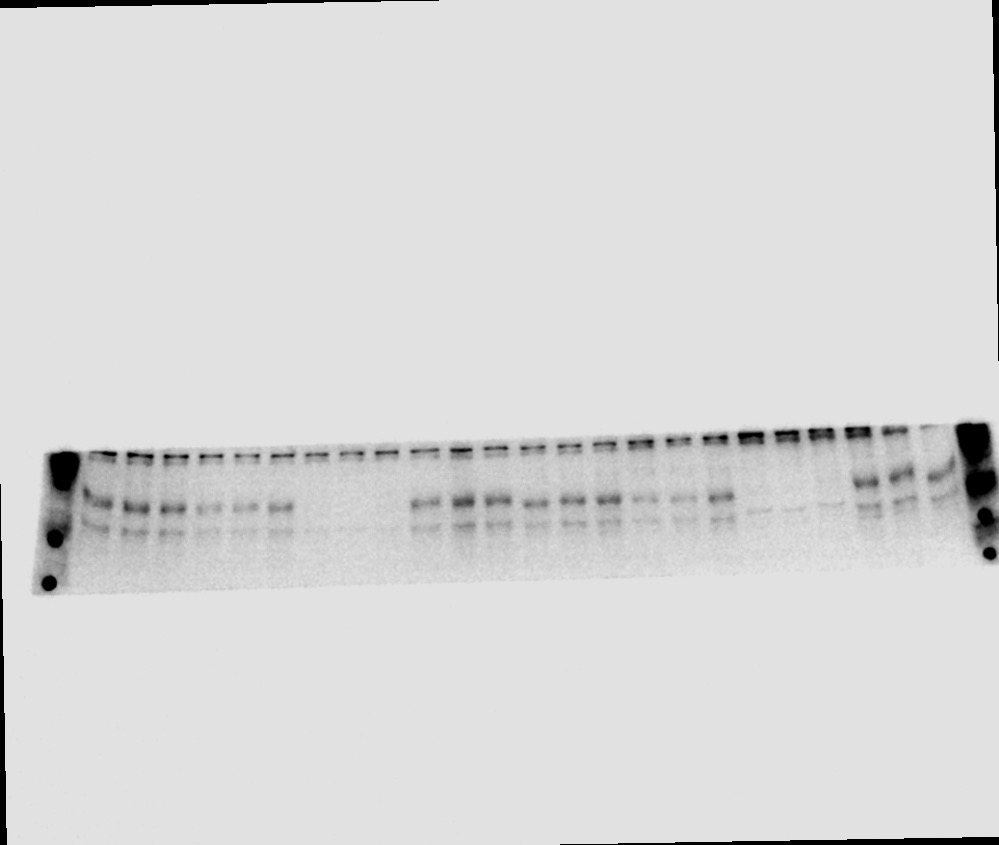

Supplement: Figure 6—figure supplement 3—source data 1. [file elife-97577-fig6-figsupp3-data1.zip › FigureS5E_SourceData1/FigS5E_MTF2.jpeg]

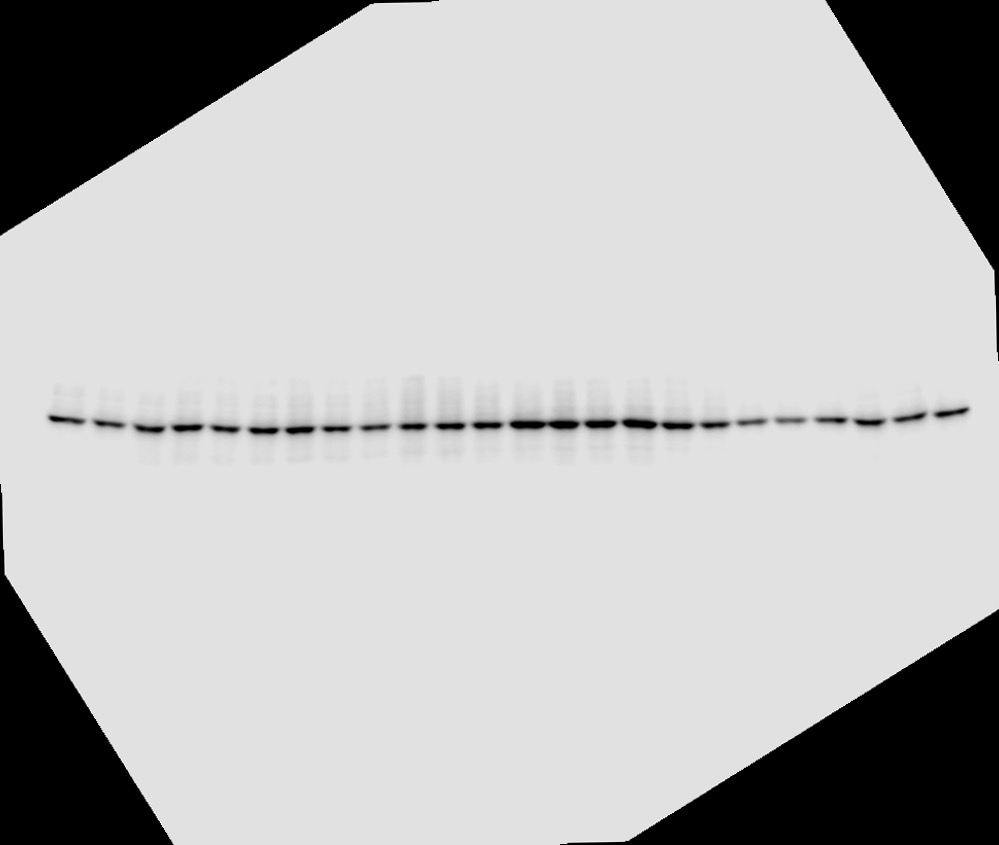

Supplement: Figure 6—figure supplement 3—source data 1. [file elife-97577-fig6-figsupp3-data1.zip › FigureS5E_SourceData1/FigS5E_CDK4.jpeg]

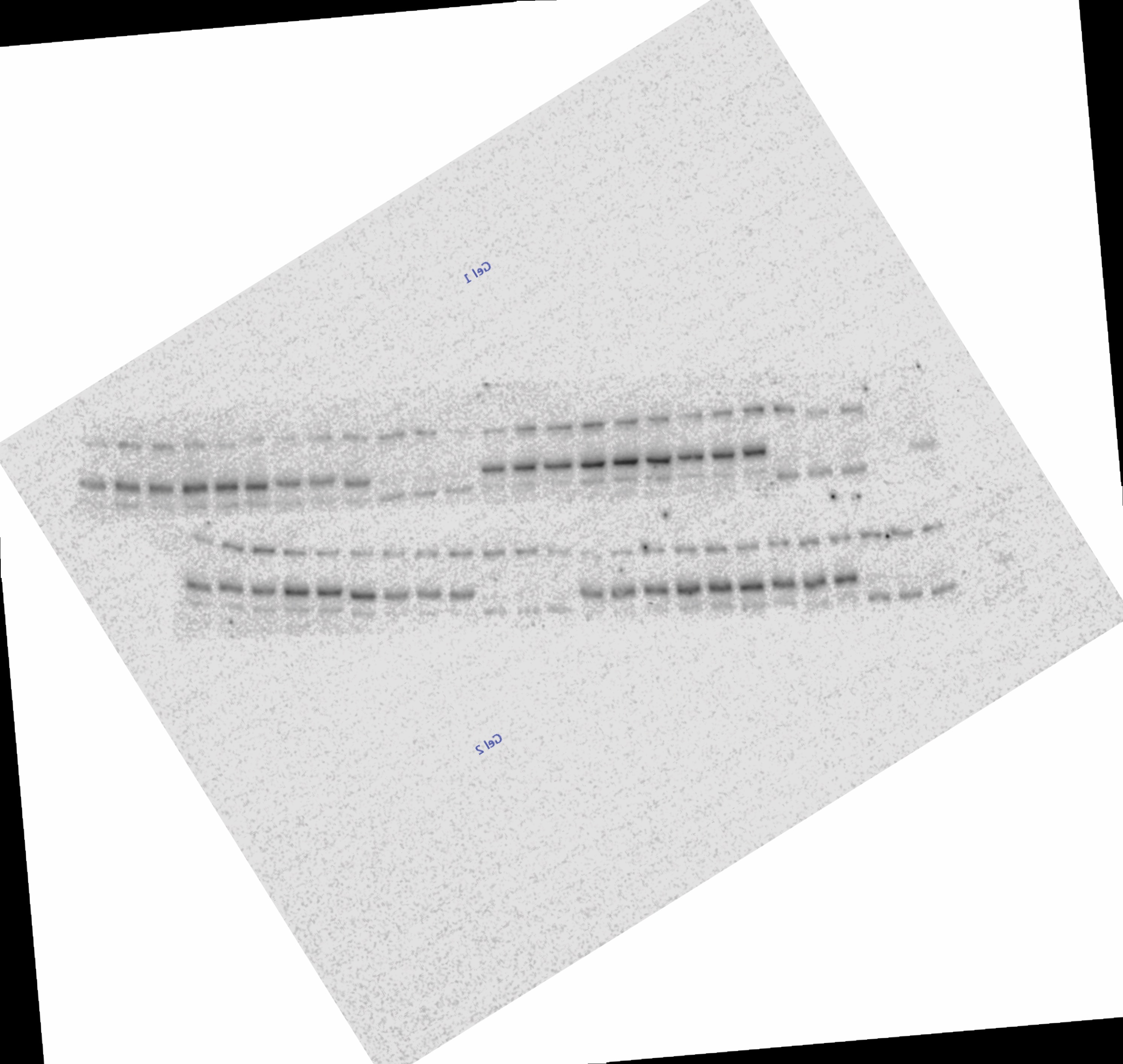

Supplement: Figure 6—figure supplement 3—source data 1. [file elife-97577-fig6-figsupp3-data1.zip › FigureS5E_SourceData1/FigS5E_JARID2.jpeg]

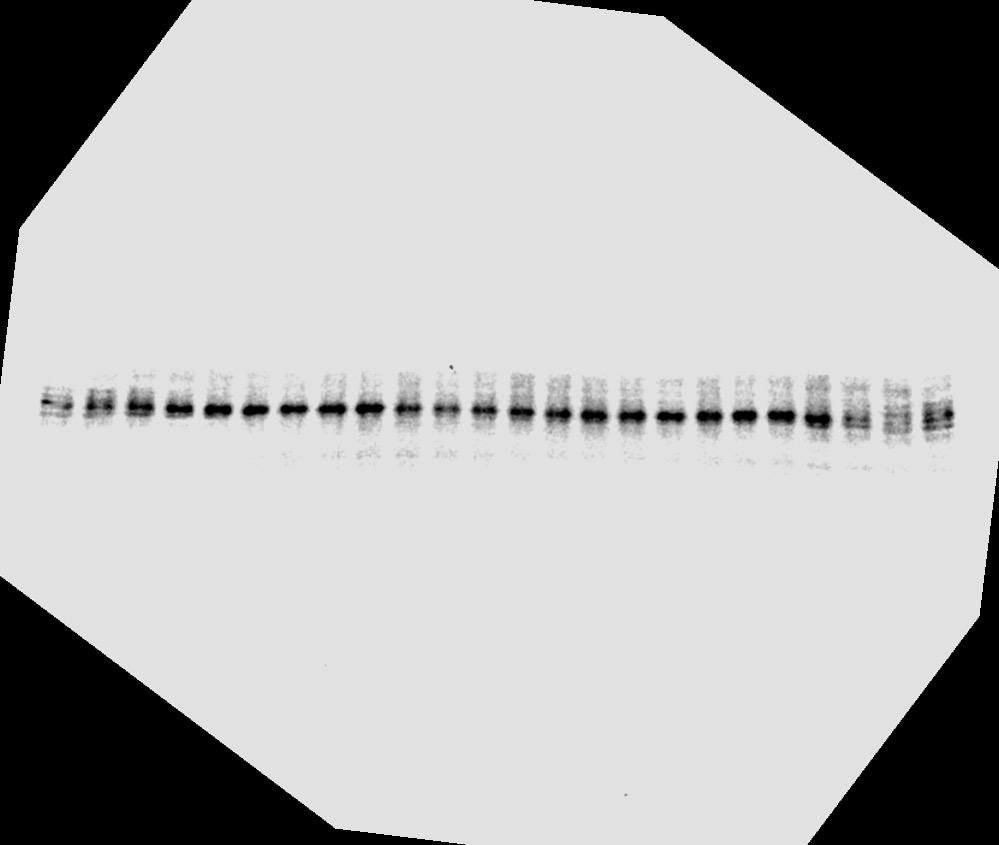

Supplement: Figure 6—figure supplement 3—source data 1. [file elife-97577-fig6-figsupp3-data1.zip › FigureS5E_SourceData1/FigS5E_CDK6.jpeg]

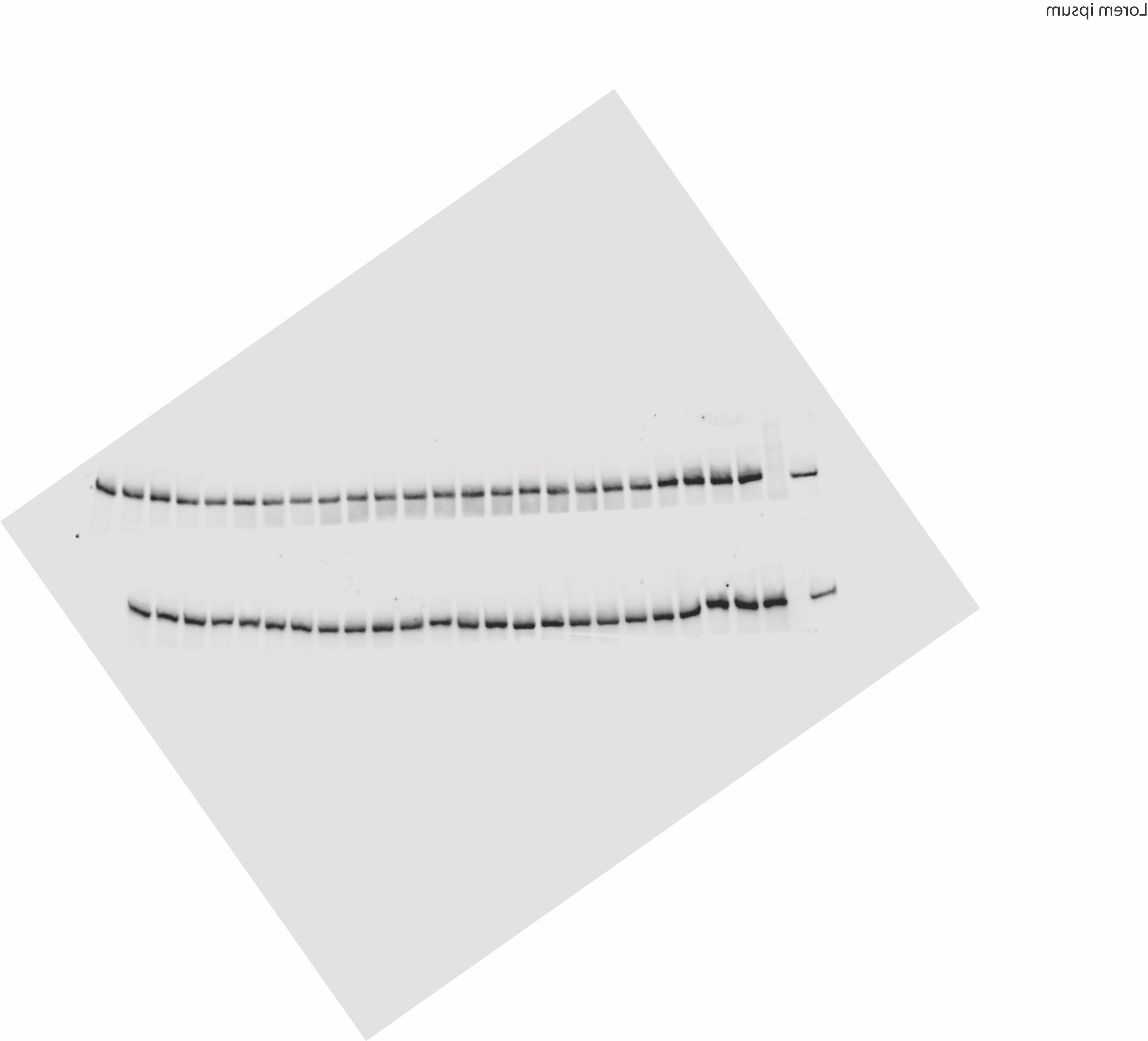

Supplement: Figure 6—figure supplement 3—source data 1. [file elife-97577-fig6-figsupp3-data1.zip › FigureS5E_SourceData1/FigS5E_Vinc.jpeg]

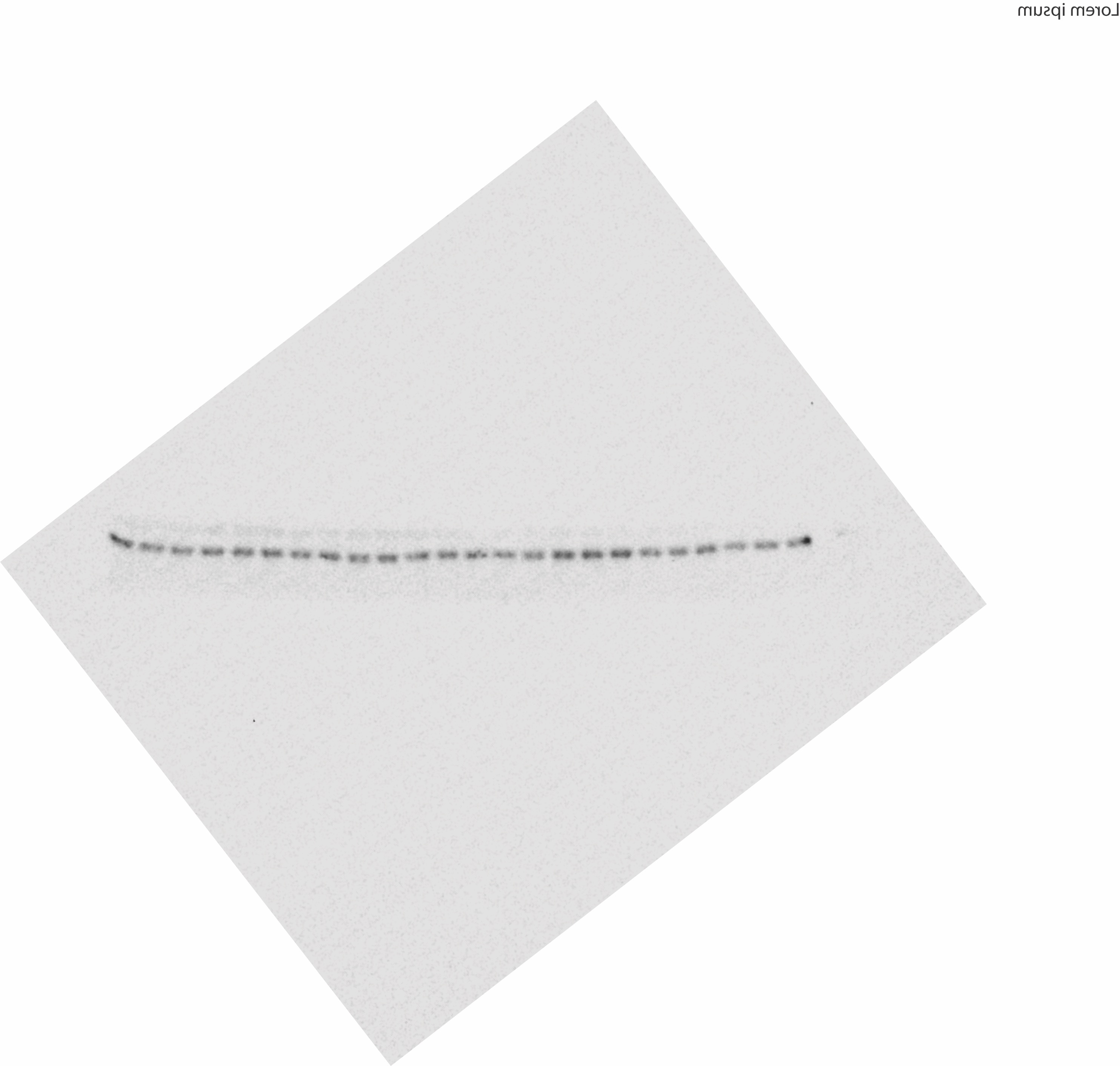

Supplement: Figure 6—figure supplement 3—source data 1. [file elife-97577-fig6-figsupp3-data1.zip › FigureS5E_SourceData1/FigS5E_p27.jpeg]

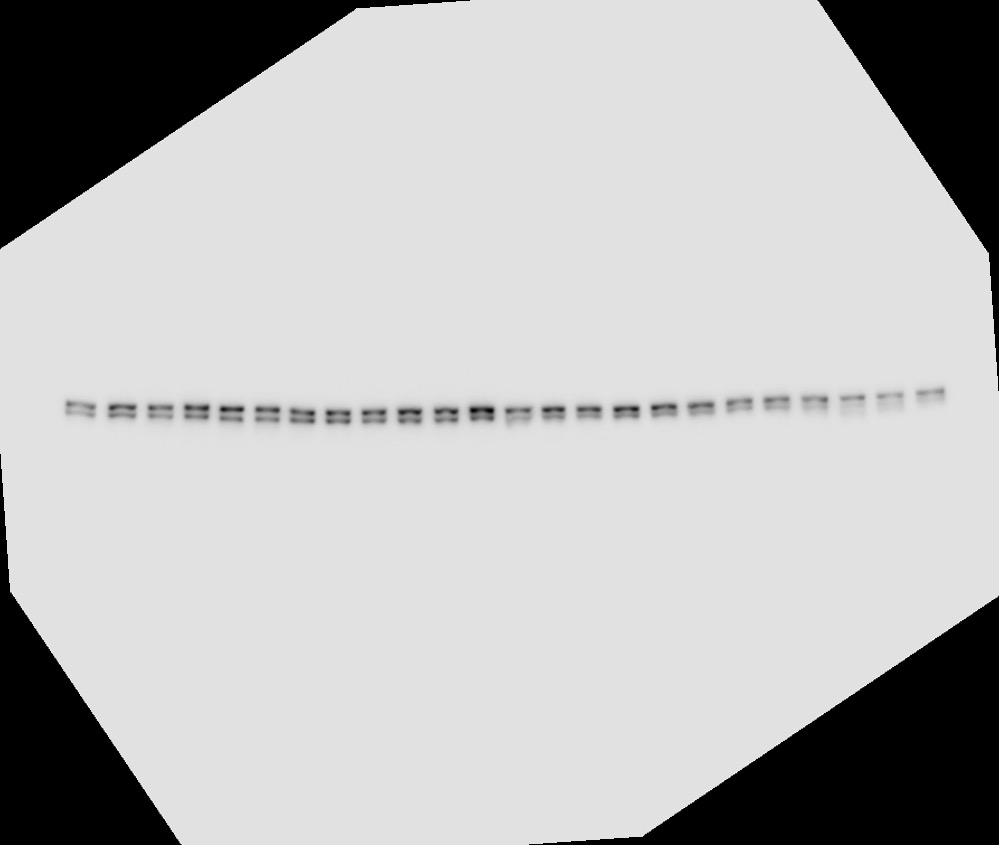

Supplement: Figure 6—figure supplement 3—source data 1. [file elife-97577-fig6-figsupp3-data1.zip › FigureS5E_SourceData1/FigS5E_RBL2.jpeg]

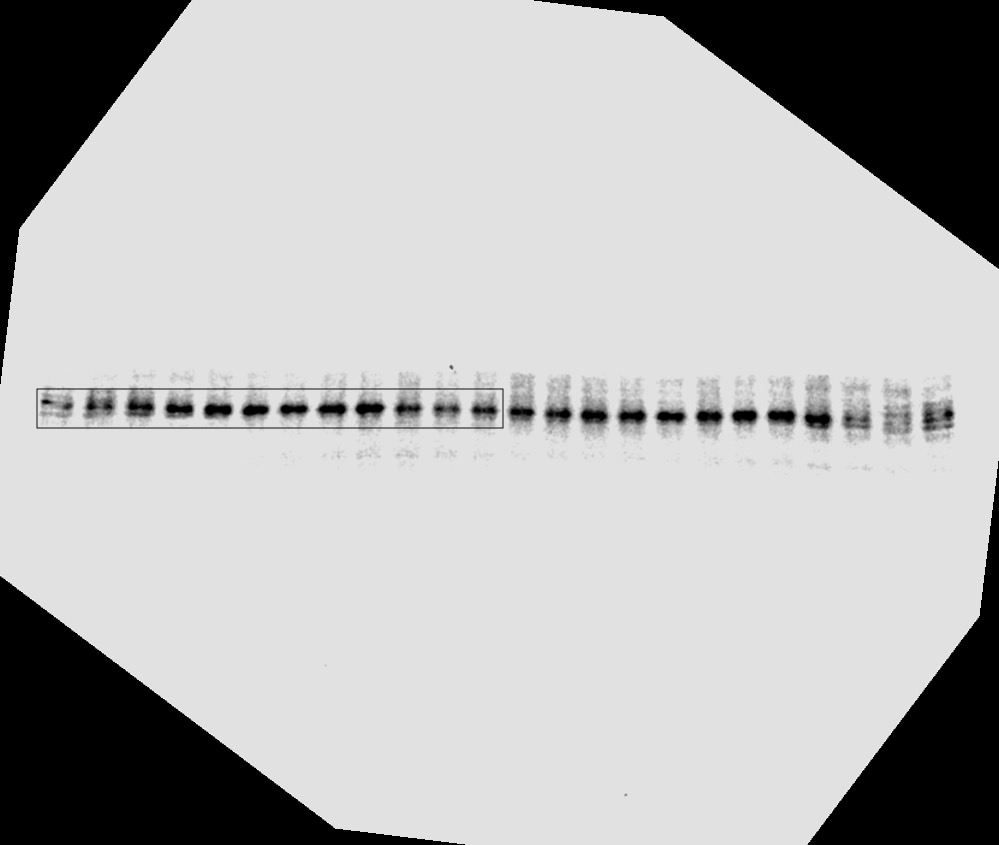

Supplement: Figure 6—figure supplement 3—source data 2. [file elife-97577-fig6-figsupp3-data2.zip › FigureS5E_SourceData2/FigS5E_Box_CDK6.jpeg]

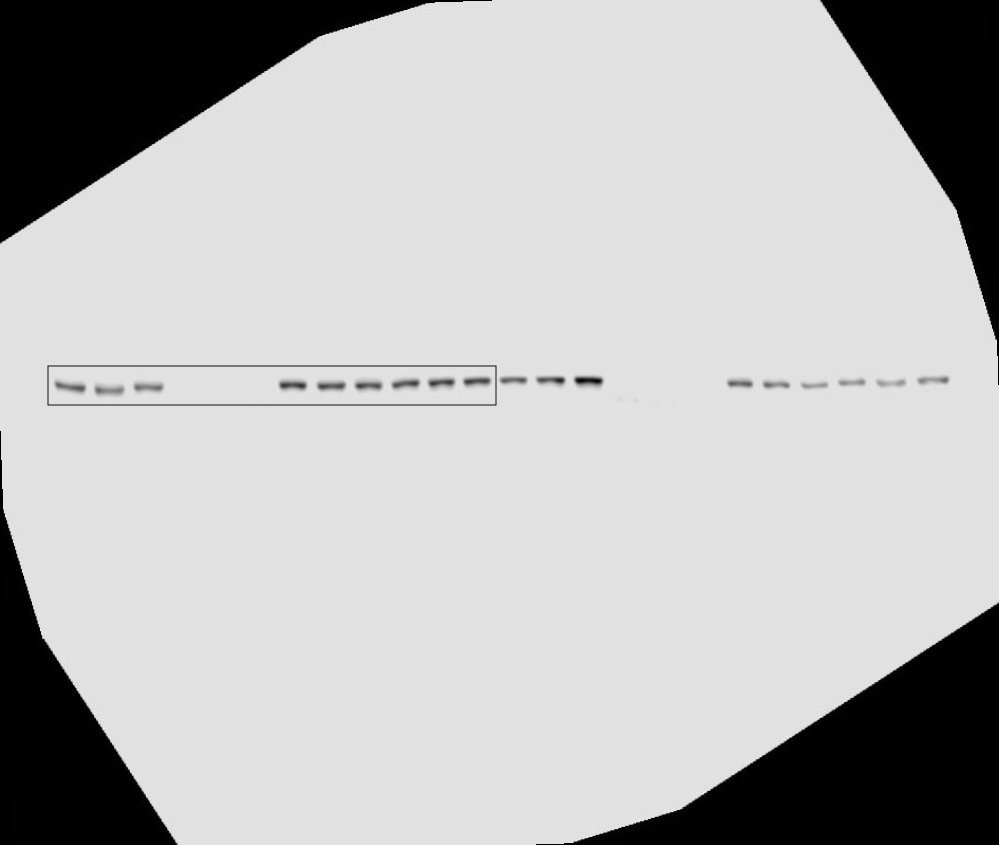

Supplement: Figure 6—figure supplement 3—source data 2. [file elife-97577-fig6-figsupp3-data2.zip › FigureS5E_SourceData2/FigS5E_Box_SUZ12.jpeg]

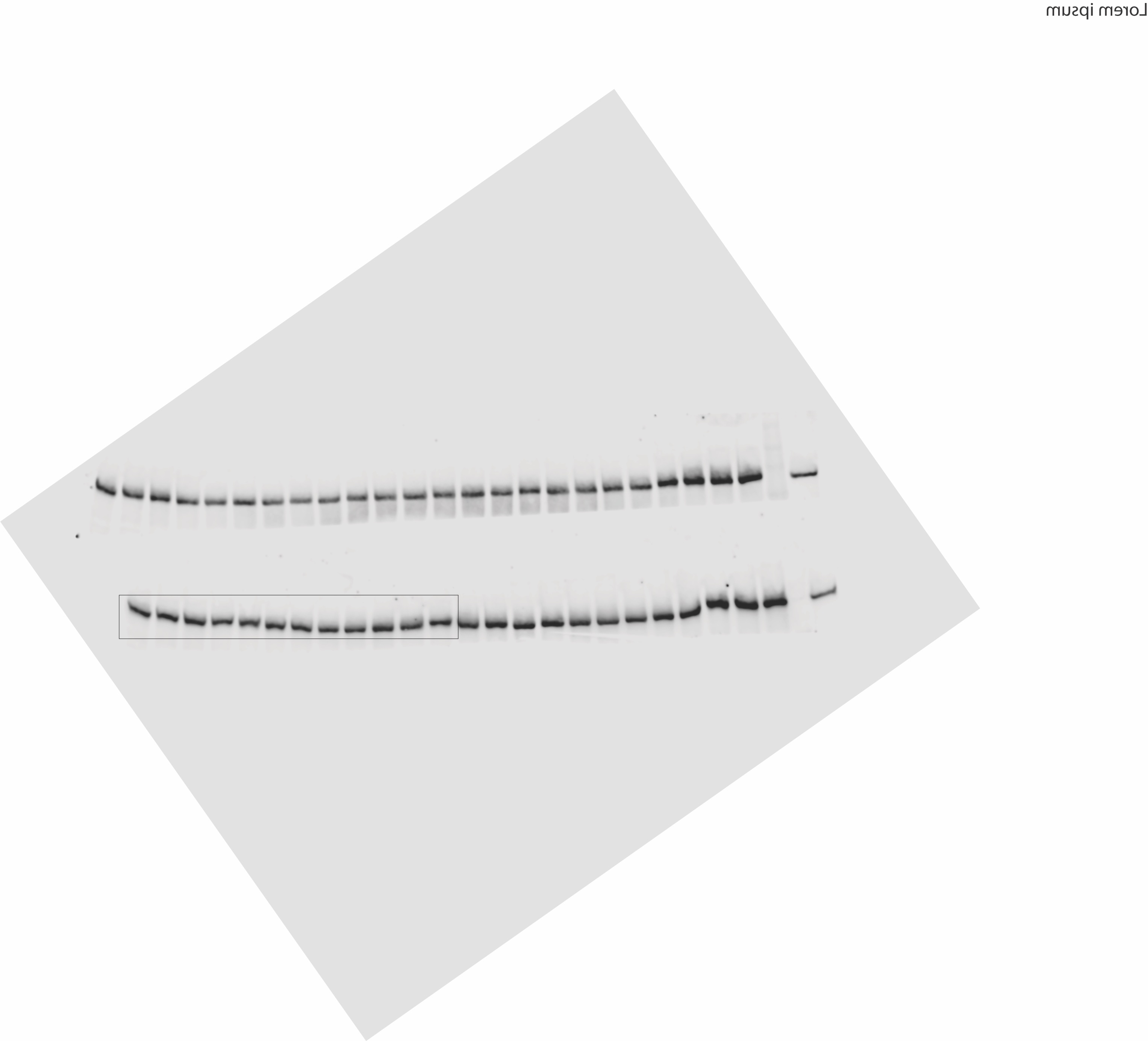

Supplement: Figure 6—figure supplement 3—source data 2. [file elife-97577-fig6-figsupp3-data2.zip › FigureS5E_SourceData2/FigS5E_Box_Vinc.jpeg]

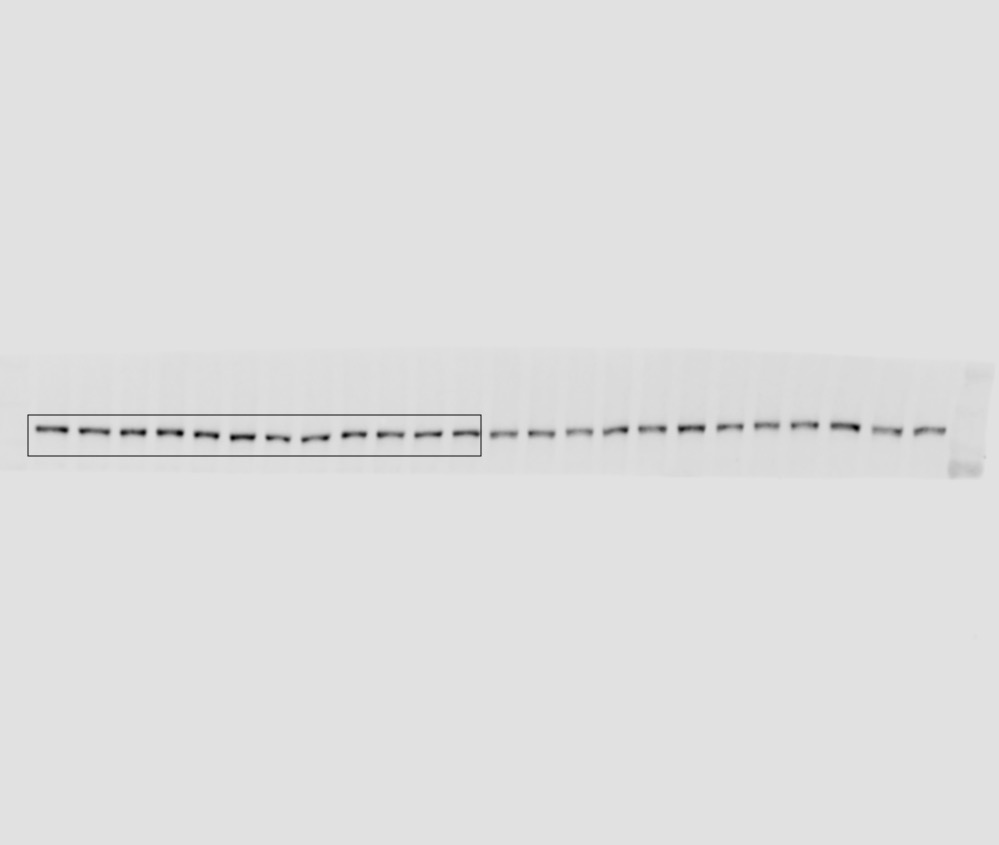

Supplement: Figure 6—figure supplement 3—source data 2. [file elife-97577-fig6-figsupp3-data2.zip › FigureS5E_SourceData2/FigS5E_Box_RB1.jpeg]

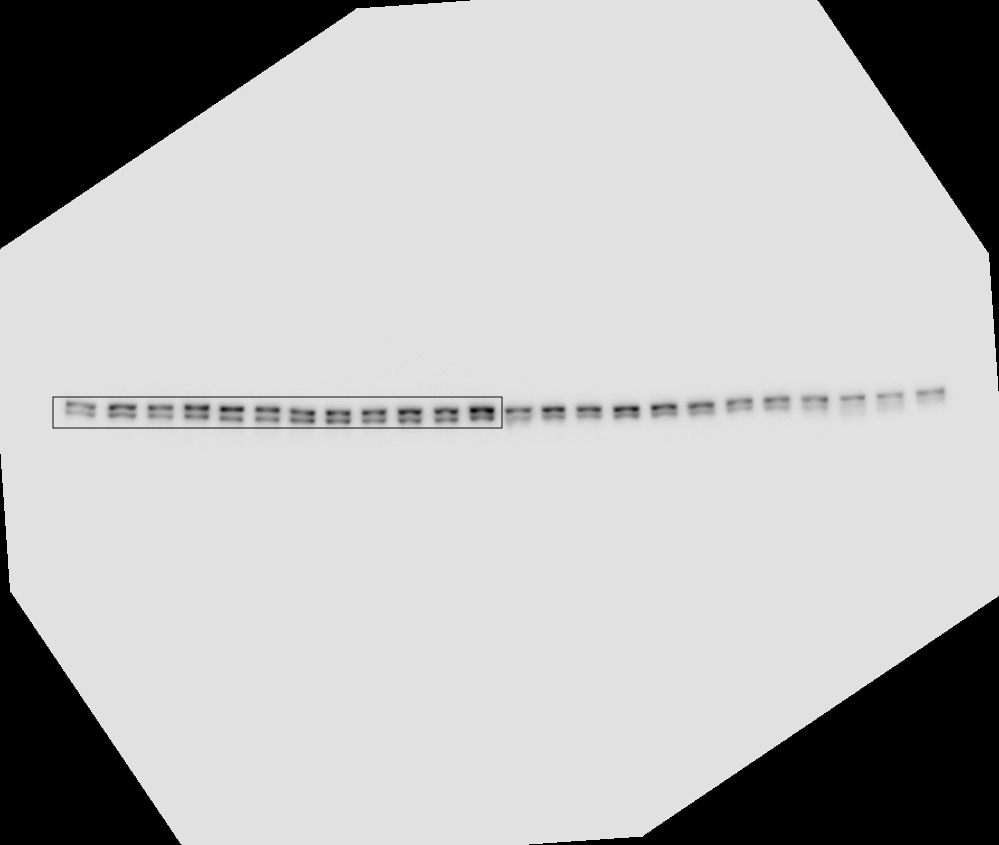

Supplement: Figure 6—figure supplement 3—source data 2. [file elife-97577-fig6-figsupp3-data2.zip › FigureS5E_SourceData2/FigS5E_Box_RBL2.jpeg]

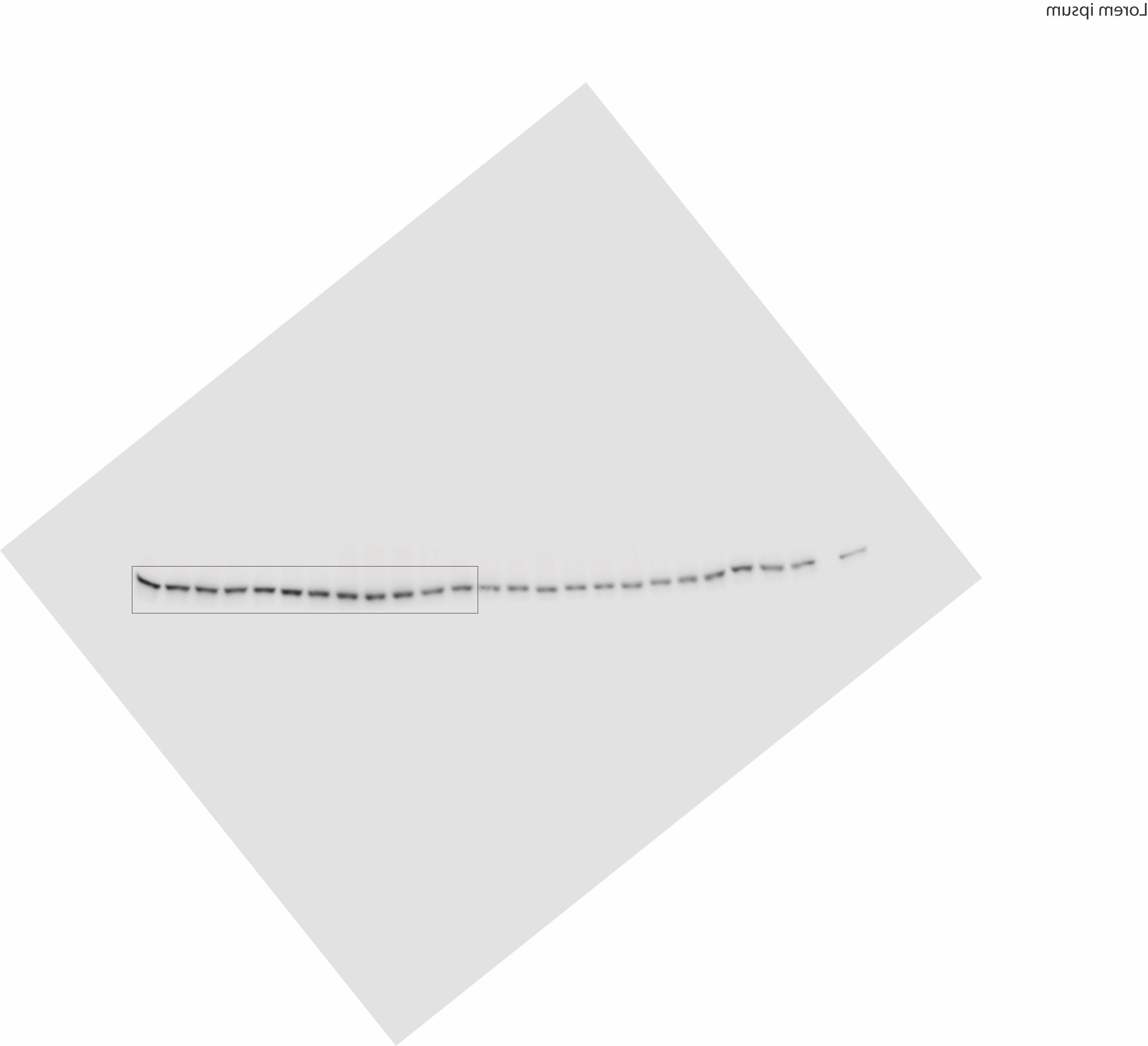

Supplement: Figure 6—figure supplement 3—source data 2. [file elife-97577-fig6-figsupp3-data2.zip › FigureS5E_SourceData2/FigS5E_Box_p53.jpeg]

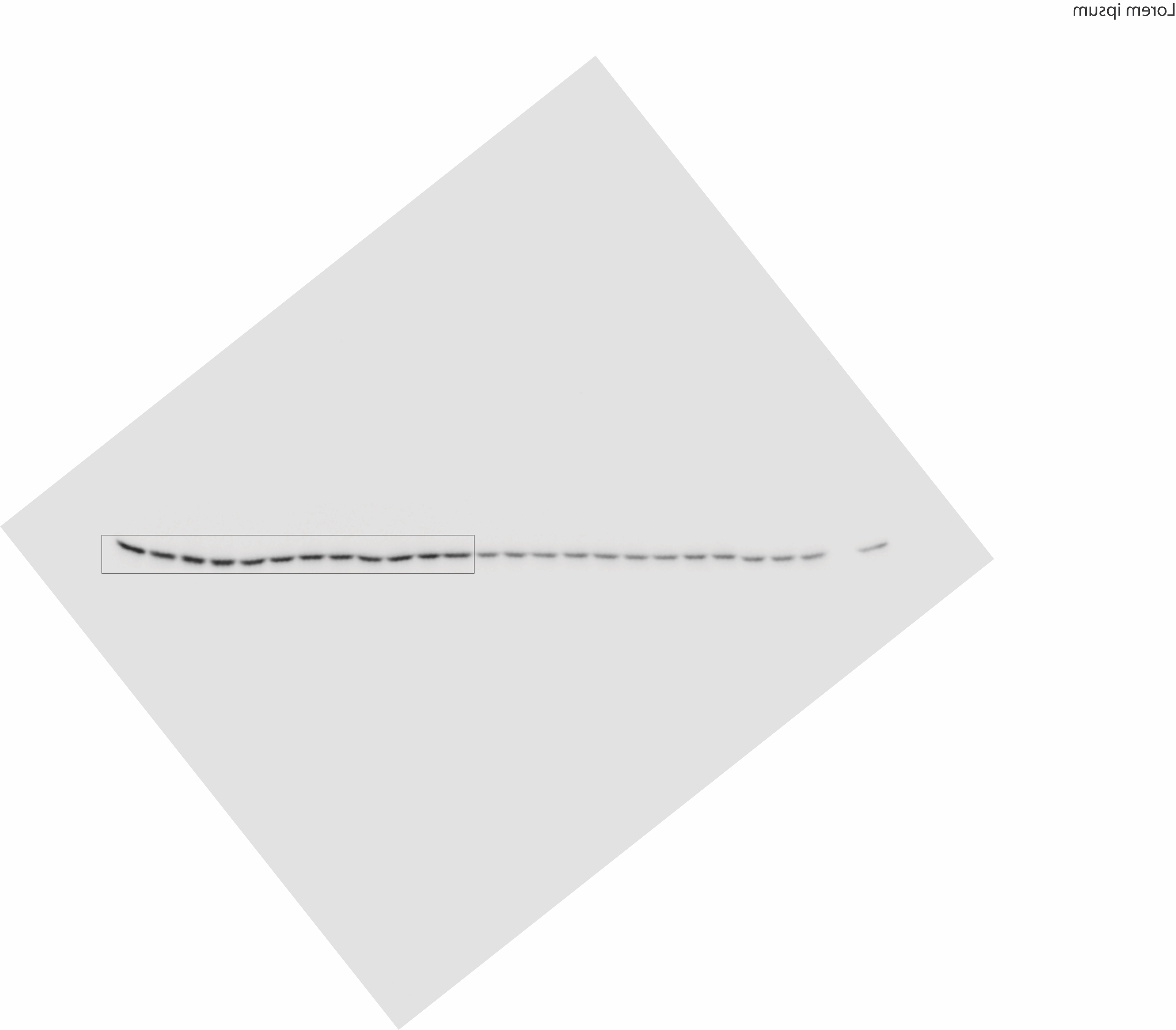

Supplement: Figure 6—figure supplement 3—source data 2. [file elife-97577-fig6-figsupp3-data2.zip › FigureS5E_SourceData2/FigS5E_Box_CDK2.jpeg]

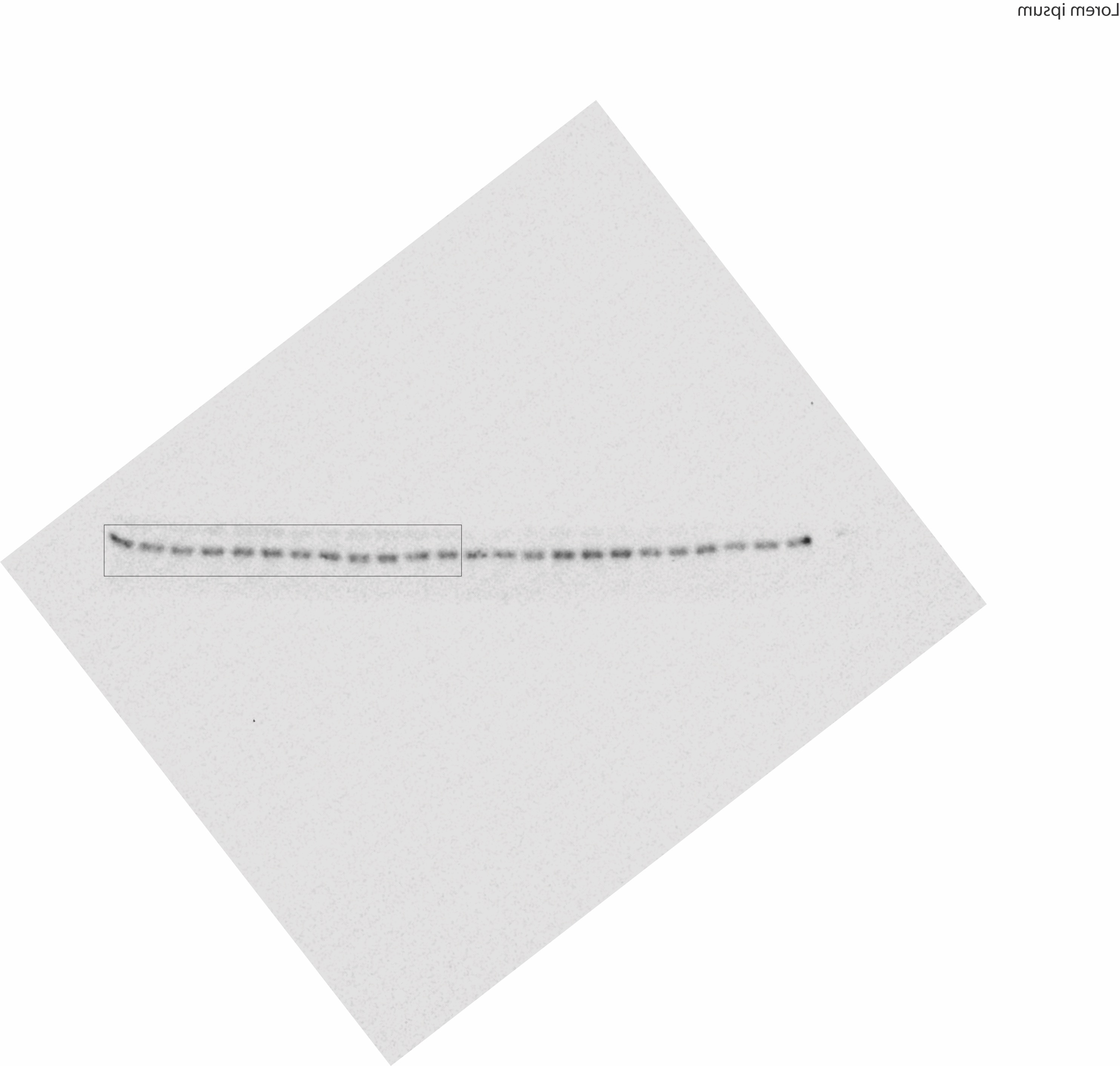

Supplement: Figure 6—figure supplement 3—source data 2. [file elife-97577-fig6-figsupp3-data2.zip › FigureS5E_SourceData2/FigS5E_Box_p27.jpeg]

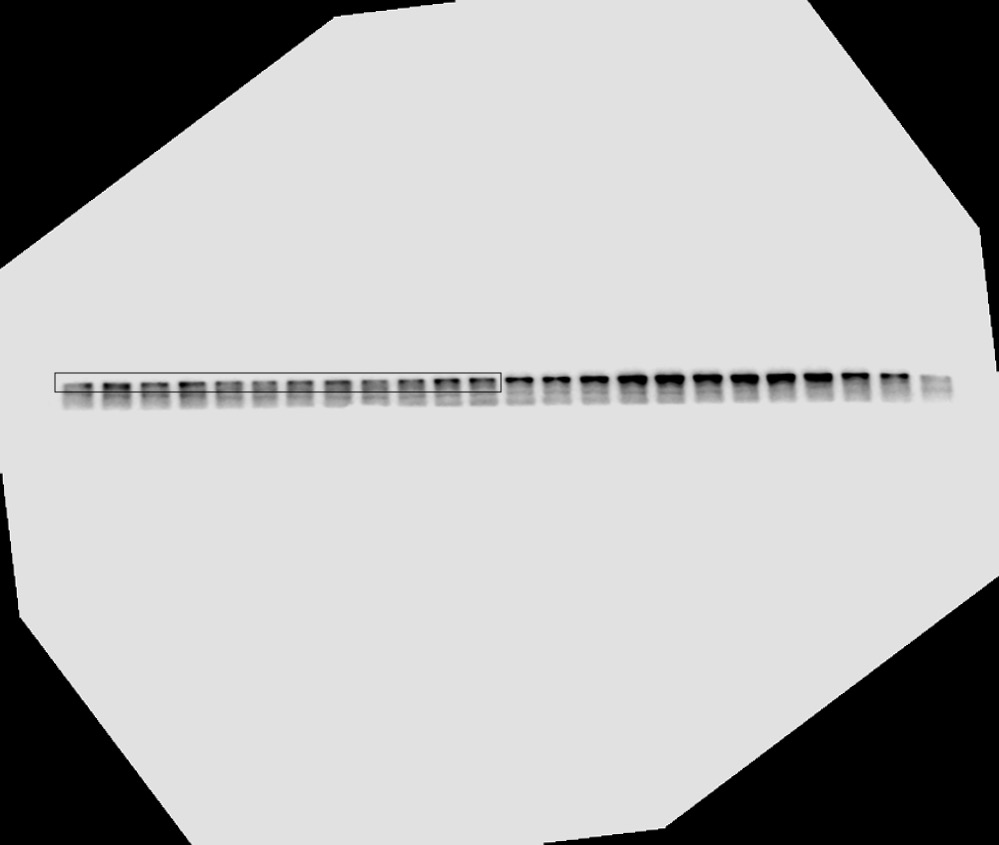

Supplement: Figure 6—figure supplement 3—source data 2. [file elife-97577-fig6-figsupp3-data2.zip › FigureS5E_SourceData2/FigS5E_Box_RBL1.jpeg]

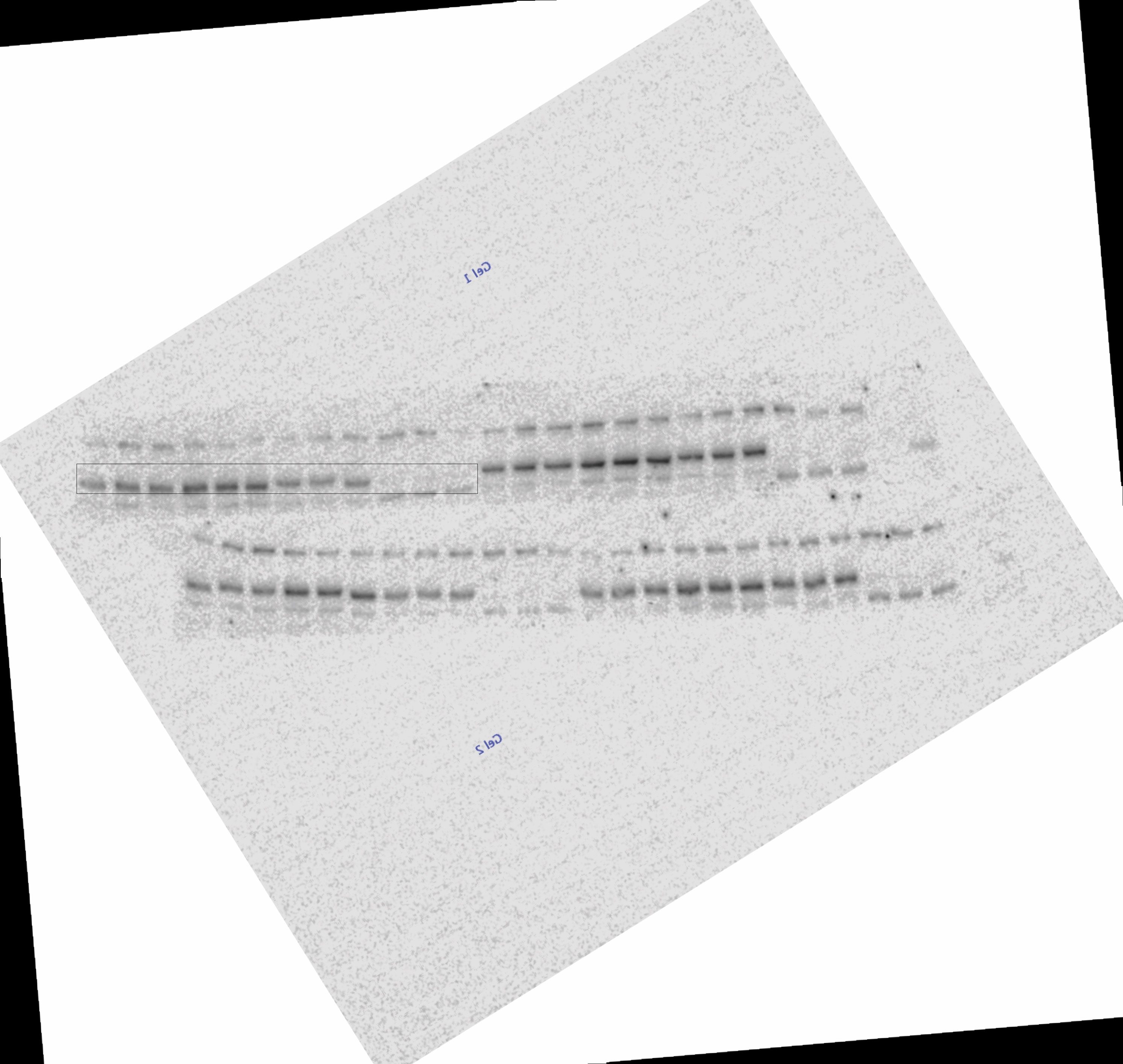

Supplement: Figure 6—figure supplement 3—source data 2. [file elife-97577-fig6-figsupp3-data2.zip › FigureS5E_SourceData2/FigS5E_Box_JARID2.jpeg]

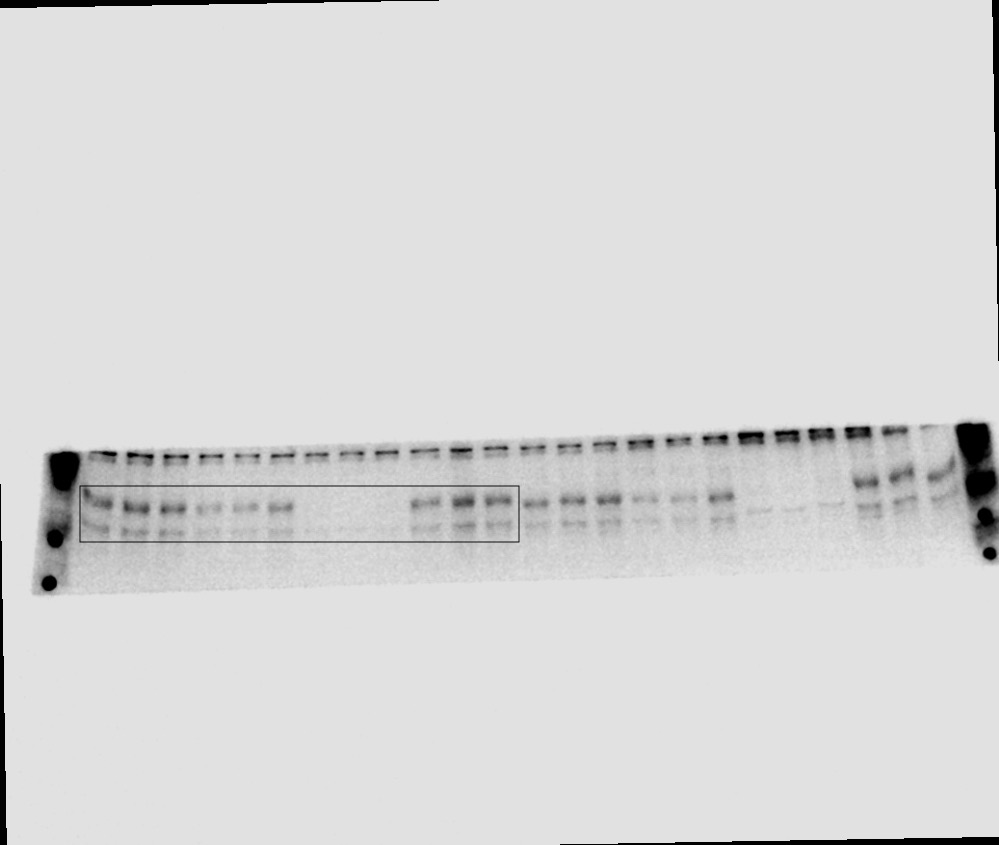

Supplement: Figure 6—figure supplement 3—source data 2. [file elife-97577-fig6-figsupp3-data2.zip › FigureS5E_SourceData2/FigS5E_Box_MTF2.jpeg]

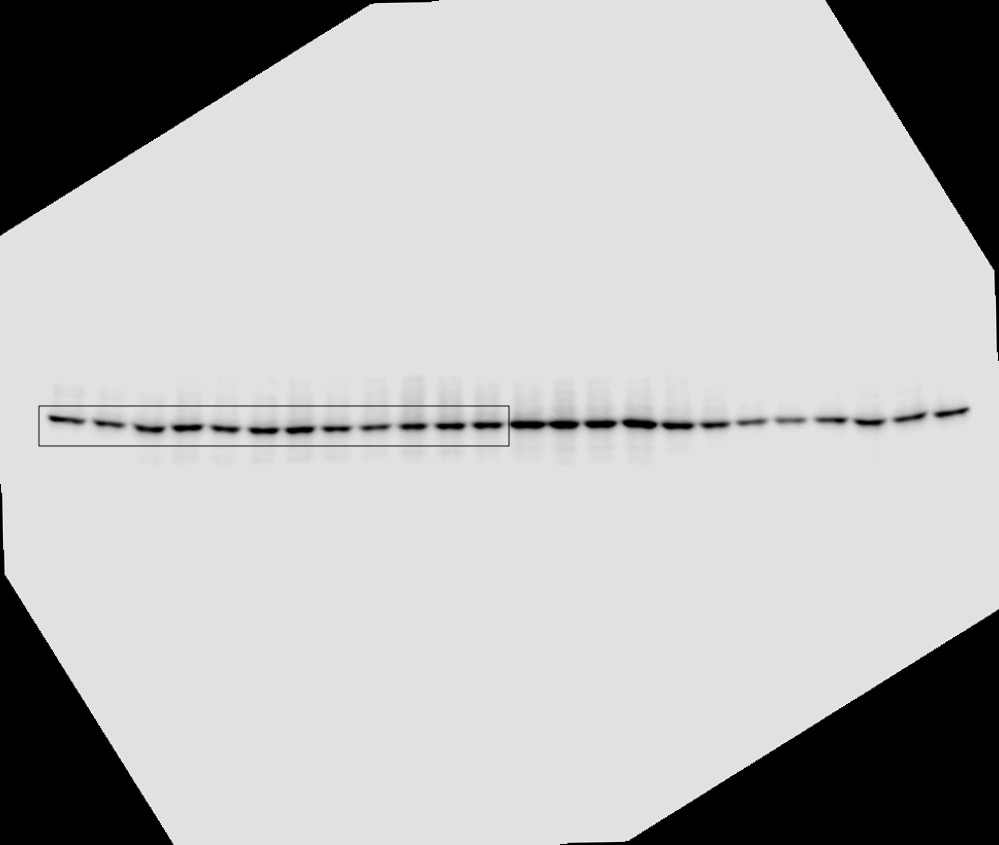

Supplement: Figure 6—figure supplement 3—source data 2. [file elife-97577-fig6-figsupp3-data2.zip › FigureS5E_SourceData2/FigS5E_Box_CDK4.jpeg]

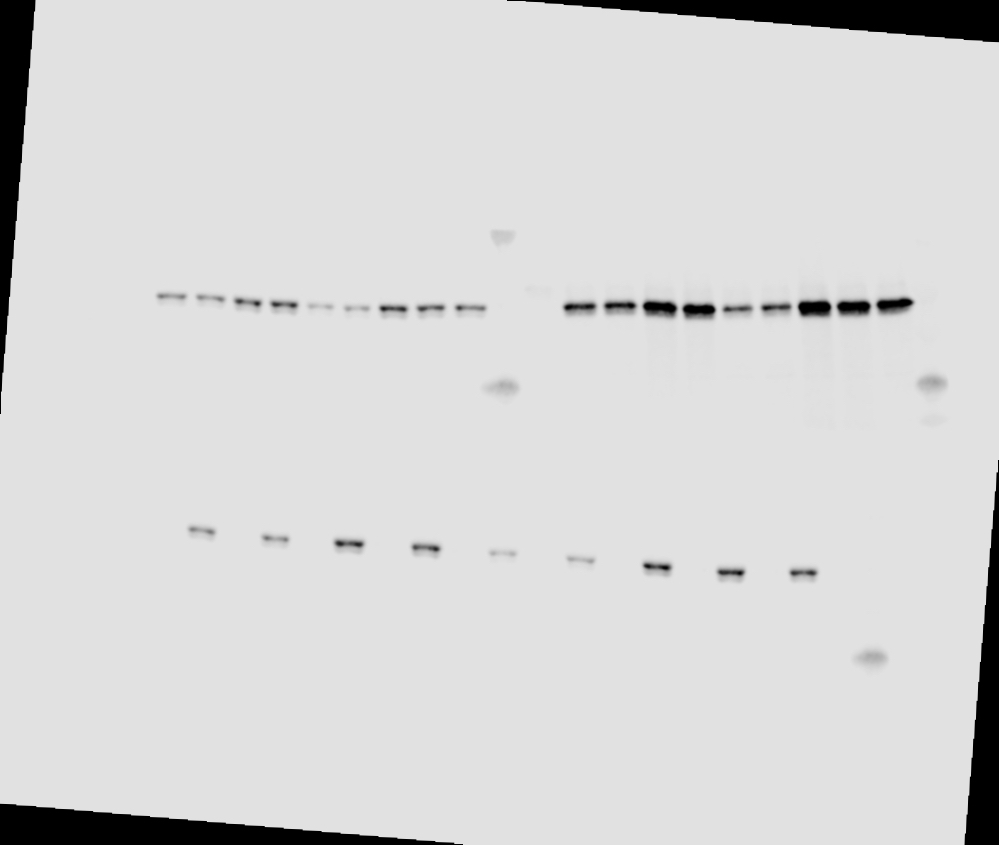

Supplement: Figure 6—figure supplement 4—source data 1. [file elife-97577-fig6-figsupp4-data1.zip › FigureS5G_SourceData1/FigS5G_2xFLAG-CDK6.jpeg]

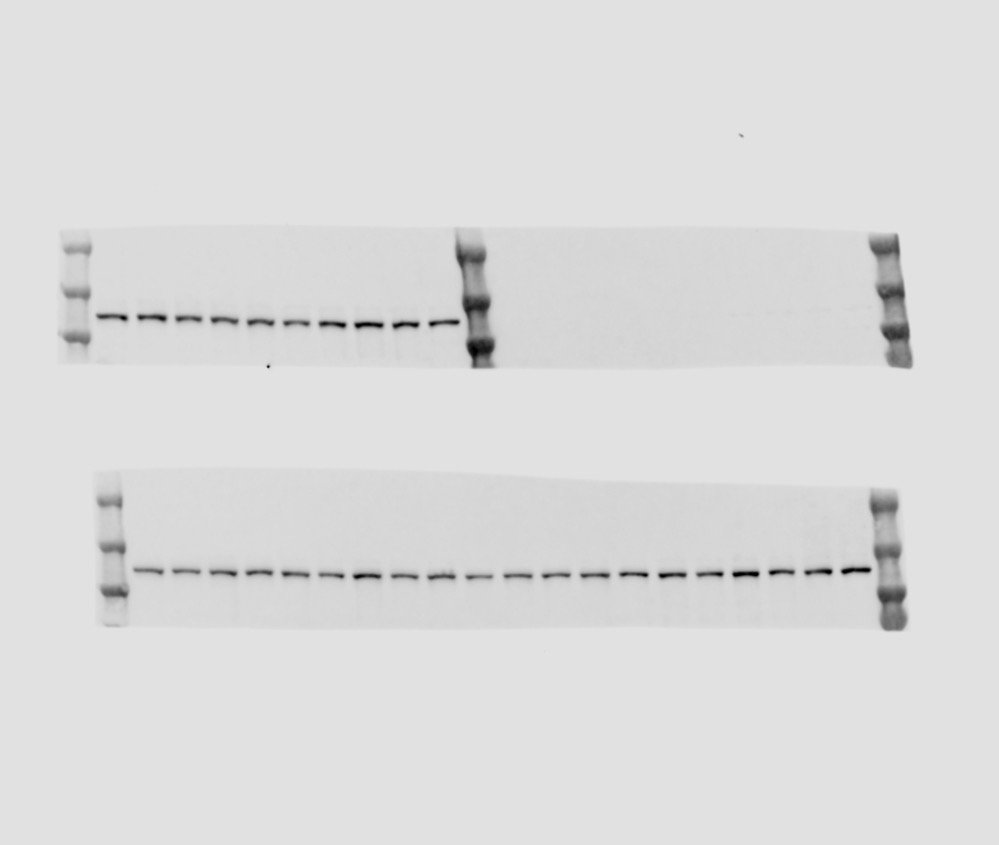

Supplement: Figure 6—figure supplement 4—source data 1. [file elife-97577-fig6-figsupp4-data1.zip › FigureS5G_SourceData1/FigS5G_Vinc.jpeg]

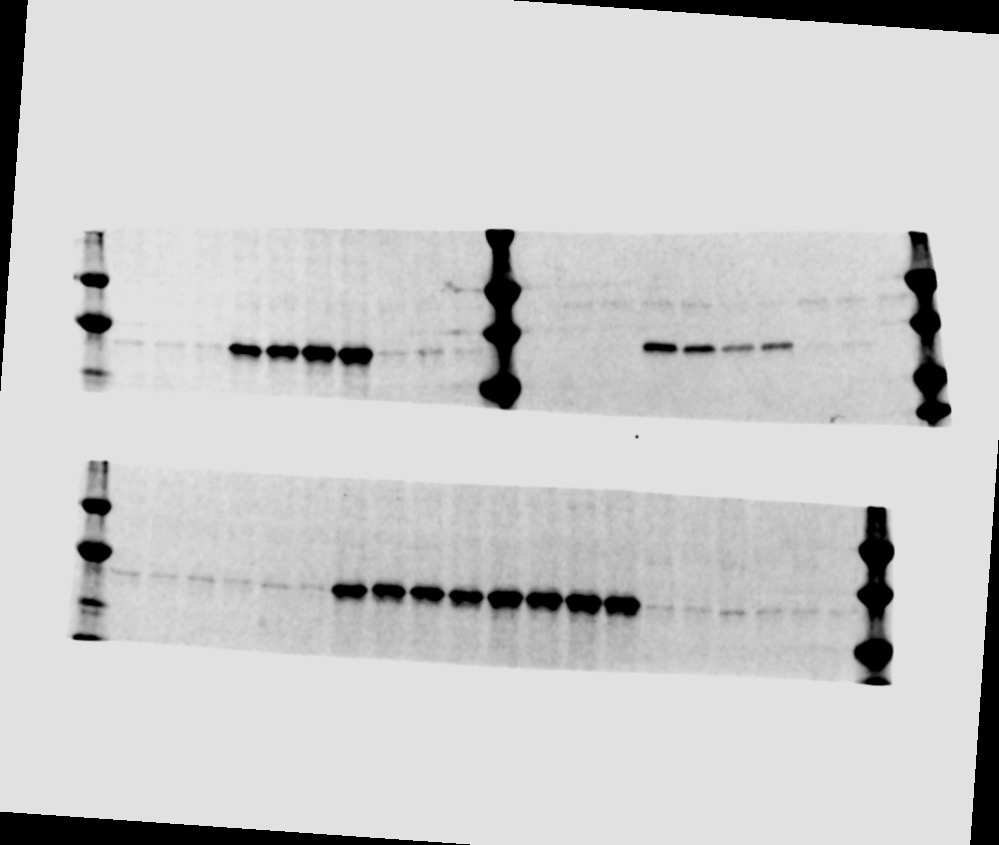

Supplement: Figure 6—figure supplement 4—source data 1. [file elife-97577-fig6-figsupp4-data1.zip › FigureS5G_SourceData1/FigS5G_CCND1.jpeg]

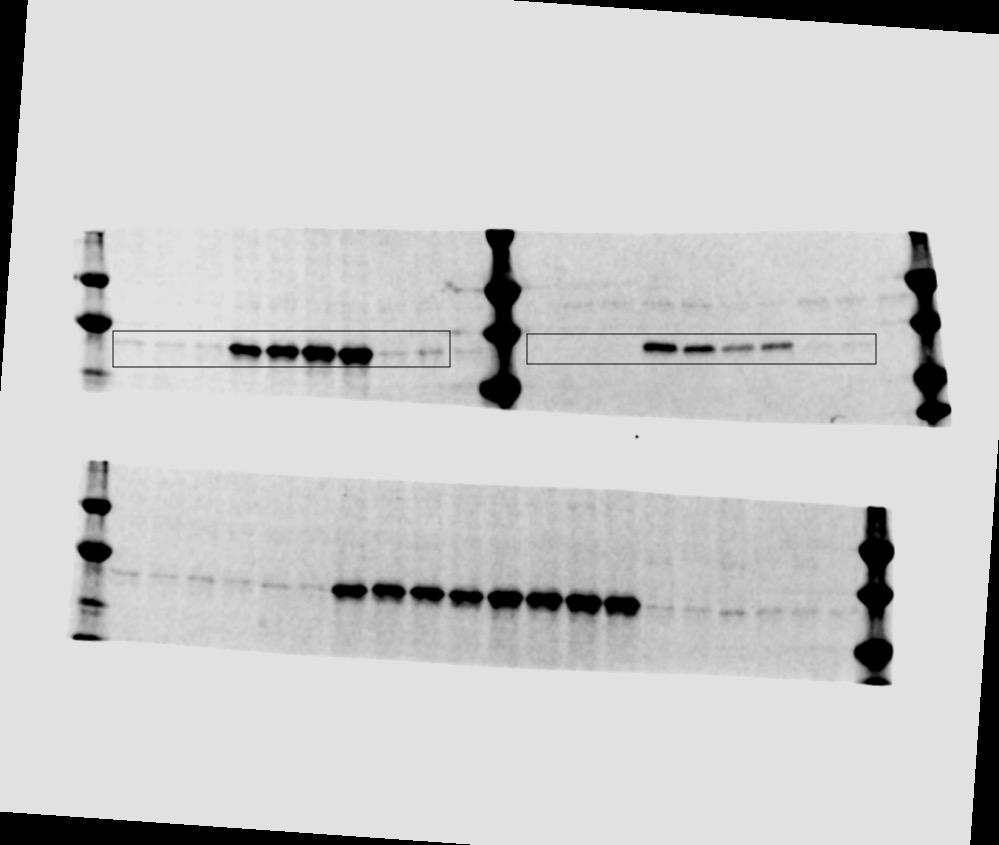

Supplement: Figure 6—figure supplement 4—source data 2. [file elife-97577-fig6-figsupp4-data2.zip › FigureS5G_SourceData2/FigS5G_Box_CCND1.jpeg]

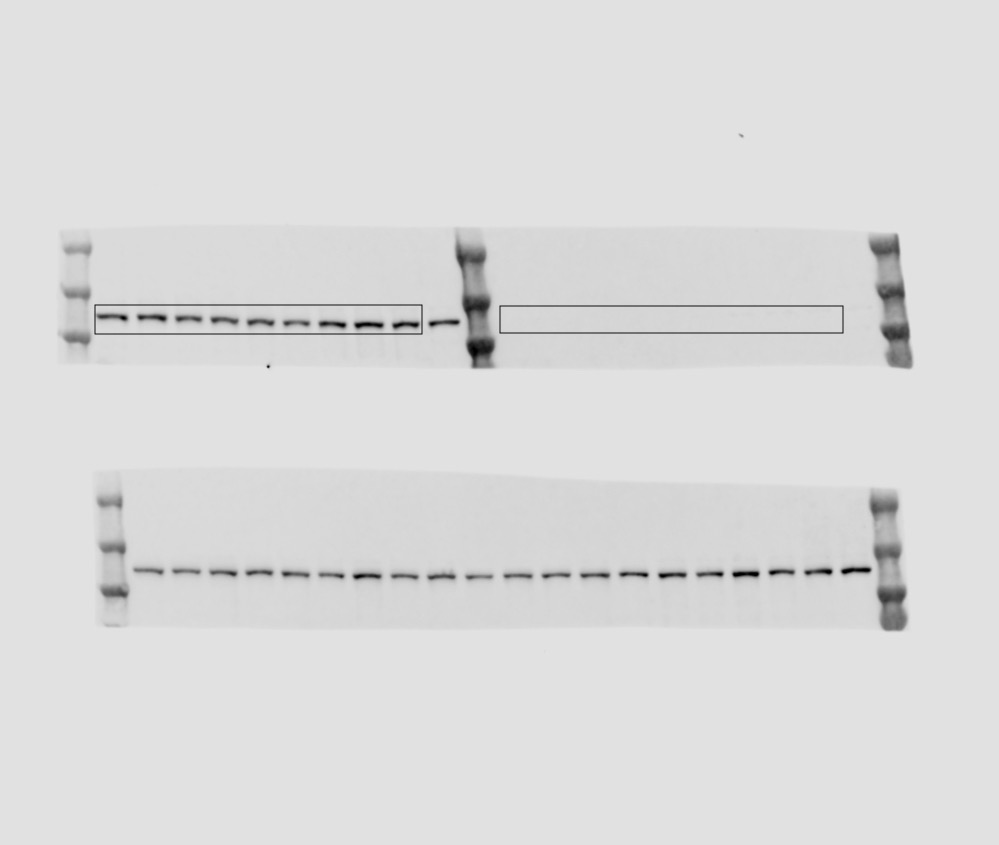

Supplement: Figure 6—figure supplement 4—source data 2. [file elife-97577-fig6-figsupp4-data2.zip › FigureS5G_SourceData2/FigS5G_Box_Vinc.jpeg]

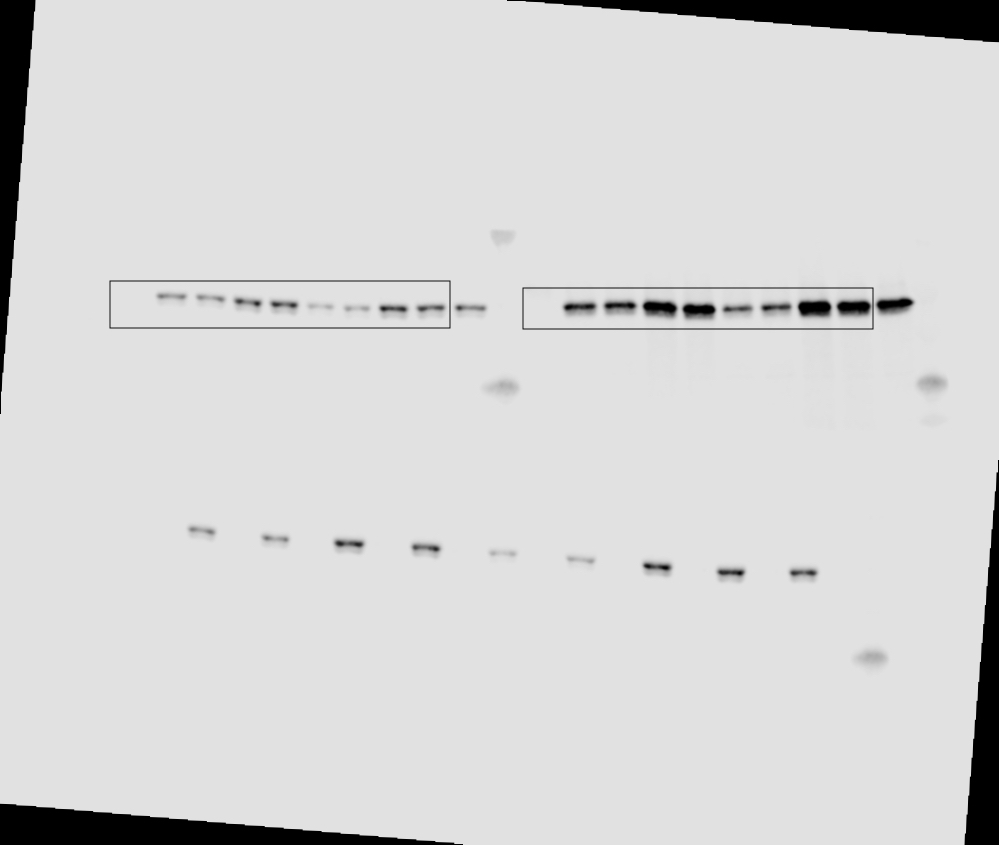

Supplement: Figure 6—figure supplement 4—source data 2. [file elife-97577-fig6-figsupp4-data2.zip › FigureS5G_SourceData2/FigS5G_Box_2xFLAG-CDK6.jpeg]
